# Scalable Predictive Analysis in Critically Ill Patients Using a Visual Open Data Analysis Platform

**DOI:** 10.1371/journal.pone.0145791

**Published:** 2016-01-05

**Authors:** Sven Van Poucke, Zhongheng Zhang, Martin Schmitz, Milan Vukicevic, Margot Vander Laenen, Leo Anthony Celi, Cathy De Deyne

**Affiliations:** 1 Department of Anesthesiology, Intensive Care, Emergency Medicine and Pain Therapy, Ziekenhuis Oost-Limburg, Genk, Belgium; 2 Department of Critical Care Medicine, Jinhua Hospital of Zhejiang University, Zhejiang, P.R. China; 3 RapidMiner GmbH, Dortmund, Germany; 4 Department of Organizational Sciences, University of Belgrade, Belgrade, Serbia; 5 MIT Institute for Medical Engineering and Science, Massachusetts Institute of Technology, Cambridge, Massachusetts, United States; 6 Limburg Clinical Research Program, Faculty of Medicine, University Hasselt UH, Hasselt, Belgium; Garvan Institute of Medical Research, AUSTRALIA

## Abstract

With the accumulation of large amounts of health related data, predictive analytics could stimulate the transformation of reactive medicine towards Predictive, Preventive and Personalized (PPPM) Medicine, ultimately affecting both cost and quality of care. However, high-dimensionality and high-complexity of the data involved, prevents data-driven methods from easy translation into clinically relevant models. Additionally, the application of cutting edge predictive methods and data manipulation require substantial programming skills, limiting its direct exploitation by medical domain experts. This leaves a gap between potential and actual data usage. In this study, the authors address this problem by focusing on open, visual environments, suited to be applied by the medical community. Moreover, we review code free applications of big data technologies. As a showcase, a framework was developed for the meaningful use of data from critical care patients by integrating the MIMIC-II database in a data mining environment (RapidMiner) supporting scalable predictive analytics using visual tools (RapidMiner’s Radoop extension). Guided by the CRoss-Industry Standard Process for Data Mining (CRISP-DM), the ETL process (Extract, Transform, Load) was initiated by retrieving data from the MIMIC-II tables of interest. As use case, correlation of platelet count and ICU survival was quantitatively assessed. Using visual tools for ETL on Hadoop and predictive modeling in RapidMiner, we developed robust processes for automatic building, parameter optimization and evaluation of various predictive models, under different feature selection schemes. Because these processes can be easily adopted in other projects, this environment is attractive for scalable predictive analytics in health research.

## Introduction

The critical care sector generates bountiful data around the clock, which can paradoxically complicate the quest for information, knowledge, and ‘wisdom’ [1]. The accumulation of clinical data has outpaced the capacity for effective aggregation and analysis aiming to support clinical quality, patient safety and integrated patient care. Intelligent data analysis promises a more efficient representation of the complex relations between symptoms, diseases and treatment [2]. Additionally intelligent data analysis hopes for a reduction of cost of care and faster design and implementation of clinical guidelines [3]. In this respect, the secondary use of clinical and operational data could support comparative effectiveness research, data mining, and predictive analytics. Commonly used data analysis platforms in clinical practice, frequently only provide support for data integration and monitoring, leaving all the analysis and decision taking to the clinical end-users. The clinical end-user is not in the position to constantly monitor and process the large amounts of data generated by patient monitoring and diagnostics. The potential of predictive analytics is to provide the clinical end-user with validated medical decision support and ultimately leading to more Predictive, Preventive and Personalized Medicine—PPPM [4]. PPPM is an integrative concept in health care that enables to predict individual predisposition before onset of the disease, to provide targeted preventive measures and create treatment algorithms tailored to the person. PPPM relies on the potential of large amounts of heterogeneous data collected in medical environments (electronic health records, medical texts and images, laboratory tests etc), but also from external data of increasingly popular wearable devices, social media etc. Data driven predictive algorithms often fail to provide self explanatory models due to high-dimensionality and high-complexity of the data structure leading to unreliable models. Also, successful predictive analytics and application of cutting edge machine learning algorithms often demands substantial programming skills in different languages (e.g. Python or R). This migrates modeling from the domain expert to the data scientist, often missing the necessary domain expertise, and vice versa, domain experts are not able to perform ad hoc data analyses without the help of experienced analysts. This leads to slow development, adoption and exploitation of highly accurate predictive models, in particular in medical practice, where errors have significant consequences (for both patients and costs). In this paper, we address this problem by exploring the potential of visual, code free tools for predictive analytics. We also review the potential of visual platforms (RapidMiner, Knime and Weka) for big data analytics. As a showcase, we integrated the MIMIC-II database in the RapidMiner data analytics platform. Data extraction and preparation was performed on a Hadoop cluster, using RapidMiner’s Radoop extension (S1 File). Further, we used RapidMiner Studio in order to develop several processes that allow automatic feature selection, parameter optimization and model evaluation (S2 File). The process compared several learning methods (Decision Stump, Decision Tree, Naive Bayes, Logistic Regression, Random Forest, AdaBoost, Bagging, Stacking, Support Vector Machine) in association with feature weighting and selection quantitatively assessed in terms of Correlation, Gini Selection, Information Gain and ReliefF.

### Scalable Predictive Analytics and Visual Open Platforms

The need for scalable and efficient frameworks, accessible to users with various levels of expertise, was recently emphasized by Koliopoulos et al. [5]. These frameworks allow data analysts and domain experts to focus on effective knowledge extraction and model tuning instead of learning new programming languages. There are multiple tools supporting open-source development with highly involved communities. This leads to faster implementation and deployment of cutting edge methods provided in the literature. The authors only address open-source, visual tools in this paper. Reviews covering open, visual tools for data analyses have been previously published, identifying RapidMiner, Knime and Weka as platforms with the highest potential for scalable big data analytics [6,7]. RapidMiner and Knime are also identified as leaders in advanced analytics platforms by Gartner [8]. In this paper, we briefly describe the available open technologies for predictive analysis of big data supported by visual tools, followed by an overview of the visual tools allowing code free big data analytics [9].

First, Google introduced **MapReduce** allowing big data processing on clusters with Mapping (parallel processing of dataset partitions) and Reducing (aggregation of the results), assuring fault-tolerance computation through replication [10].

Further, Yahoo developed **Hadoop** as an open source implementation of MapReduce [11]. The Hadoop Distributed File System (HDFS) is a disk-based file system that spans across the nodes of a distributed system. HDFS encapsulates distributed local storage into a single logical unit and allows automatic division of data into blocks and replication on local disks, allowing fault-tolerance computations. Map/reduce jobs on Hadoop can be developed on **Hive** [12], enabling querying and managing large data on distributed storage. It provides a mechanism to project structure on this data and query the data using an SQL-like language called HiveQL. It also allows definition and execution of map/reduce map reduce jobs in other languages when required. Vavilapalli et al. [13] noticed that the major disadvantage of Hadoop for general purpose analytics is the thight coupling of a specific programming model with the resource management infrastructure. In order to overcome this, a new architecture was developed, called **YARN** (Yet Another Resource Negotiator) that decouples the programming model from the resource management infrastructure and delegates many scheduling functions (e.g., task fault tolerance) to per-application components. Vavilapalli et al. provided experimental evidence of improved efficiency of running YARN on production environments and many projects in the Hadoop ecosystem support work on YARN with almost the same feature set as on Hadoop: e.g. **Hive** [12], or **Pig** [14].

In order to exploit the potentials of Hadoop for predictive analytics, **Mahout** was developed providing scalable data mining libraries [15]. Even though Mahout is widely used for scalable predictive analytics, it is also criticized, as its libraries do not provide a general framework for building algorithms, the quality of the provided solutions varies significantly being dependent on the contributor expertise [5, 16]. Mahout also focuses on implementing specific algorithms, rather than building execution models for algorithm methods.

Hadoop and its ecosystem gain high popularity over the years, but processing data from disk made it inefficient for many data mining applications that often require iteration, which is not easily performed in MapReduce [5, 9].

In order to tackle these insufficiencies, **Spark**, was developed allowing in-memory and iterative computation [17]. Spark also implements the MapReduce paradigm and is Java-based (as is Hadoop). These features enable users to deploy existing Hadoop application logic in Spark via its Scala API. It is based on abstractions called **Resilient Distributed Datasets** (RDD), which store data in-memory and provide fault tolerance without replication [17]. RDDs can be understood as read-only distributed shared memory [18]. Spark has been shown to outperform Hadoop by up to two orders of magnitude in many cases.

**MLlib** is the machine learning library for Spark covering the same range of learning categories as Mahout, but also adds regression models, which is lacking in Mahout [9, 19]. MLlib’s reliance on Spark, in-memory computations and iterative batch and streaming approaches, enable jobs to run significantly faster than those using Mahout [20]. However, the fact that it is tied to Spark may present a problem for performing machine learning on multiple platforms [21]. MLlib is still relatively young compared to Mahout.

**SparkR** [22] and **PySpark** [23] provide R and Python users a lightweight front-end to a Spark system, by compiling the declarative scripts to low-level MapReduce jobs which is considered valuable taking into account the popularity of R and Python in the data science community.

**RapidMiner** (previously: Rapid-I, YALE) [24] became very popular in recent years and is supported by a large community. Its visually appealing, user friendly GUI (graphical user interface) and wiki-based contextual help (with process examples for each operator), allow ease of use and a fast learning curve. This is also supported by the “Wisdom of crowds” which provides suggestions (which operator should be used next) based on the community experience. Additionally, there are multiple extensions providing data and pre-defined processes suited for specific application areas (e.g. marketing, finance etc.) and a community is very active in sharing processes on the RapidMiner “Marketplace”. One of the important strengths of RapidMiner is its flexibility in process design through “Process/Sub-process” structures and “Macros” that represent global variables of the environment. This enables a visual design of complex processes, and a high level of automation (as presented in our experiments) which is usually possible only by programing (e.g. in R or Python). Furthermore this allows seamless parameter optimization, being a necessary step for many cutting edge algorithms (e.g. SVMs). This technique allows simple maintenance of data flows (in comparison with pure coding environments). RapidMiner also provides a large number of machine learning algorithms, tools for pre-processing and visualization, including wrappers for most of the Weka operators and simple tools for incorporation of custom-built R and Python scripts. Considering all this makes RapidMiner a powerful predictive analytics environment for data analysts and/or domain experts with variable levels of expertise. On the down side, the support for deep learning methods and some of the more advanced specific machine learning algorithms (e.g. extremely randomized trees, various inductive logic programming algorithms) is currently limited [7] but can be solved by incorporation of R and Python scripts. Additionally, the current version of Rapidminer (6.5) has a free licence [7] with very few constraints compared to the commercial version (e.g. SQL database support) and provides some of the previous versions free of charge (currently, version 5.3 is available).

RapidMiner supports scalable predictive analytics on Big data through its Radoop extension. It allows code free, visual analytics on Hadoop, Spark, Hive, MapReduce, Pig and Mahout through series of specialized operators that can be used with standard RapidMiner operators within the used workflows. Additionally, Radoop enables incorporation of SparkR, PySpark, Pig and HiveQL scripts within predictive analytics workflows. This allows seamless combination of data preparation on Spark and Hadoop with predictive analytics using Spark’s machine learning library MLlib or Radoop’s Mahout.

**Weka** (Waikato Environment for Knowledge Analysis) is a very powerful and versatile data analytics tool, also largely supported by a community and very popular in the academic world [25]. The success of Weka is related to the availability of a wide range of well implemented machine learning algorithms and model evaluation procedures and metrics. Weka also provides an API (application programming interface) for integration of its algorithms and procedures resulting in a frequent adoption in other visual environments such as RapidMiner and Knime. Even more interesting, Weka’s algorithms are also used in coding environments like R and Python through API’s. As RapidMiner and Knime, Weka allows implementation of extensions independent of the used core system resulting in a fast increasing number of Weka features provided by their community. In contrast, Weka’s GUI is not as visually appealing and extendable as RapidMiner or Knime, it lacks many data survey and visualization methods and it has the tendency to be more oriented towards classification and regression problems and less towards descriptive statistics and clustering methods [7]. This probably explains why Weka didn’t make it to the leaders quadrant in Gartners report on advanced analytics platforms [8]. Still, Weka offers a completely free and very powerful environment for code-free predictive analytics. Weka also supported R scripting for a long time and recently a “wekaPython” extension provided Python scripting within data flows defined through GUI.

Regarding big data tools, Weka does not have an integral environment for both Hadoop and Spark, but it supports big data analytics through several extensions. It allows work with Hadoop through DistributedWekaBase and DistributedWekaHadoop packages [25]. These packages provide both base "map" and "reduce" tasks that are not tied to any specific distributed platform and Hadoop-specific wrappers and jobs for these base tasks. These packages allow different data pre-processing, modeling and evaluation tasks on big data (e.g. computing correlation and covariance matrices, PCA, training, scoring and evaluation of predictive models). In case of model learning, predictive algorithms are divided into “aggregatable” and “non-aggregatable”. Aggregatable, produce a single model, that is learned in parts on a cluster and aggregated in a Reduce job while models that cannot be aggregated in a Reduce job allow making of ensemble models (e.g. Bagging) built separately on a cluster.

Recently DistributedWekaSpark, a distributed framework for in-memory cluster computation in Weka is proposed [5] allowing similar functionalities as previously described for MapReduce and Hadoop. Weka extensions for big data do not offer wrappers for machine learning algorithms already developed in Mahout or MLlib, but rather adapt its own algorithms to work with MapReduce. This makes learning more difficult for entry level analysts [5].

**KNIME (Konstanz Information Miner)** is also considered a visual open-source tool, based on the Eclipse project, but also offers commercial licenses for companies requiring professional technical support [26]. It shares good features with RapidMiner allowing a fast learning curve for entry level analytics: visually appealing and intuitive GUI, good documentation and community support, ease of development through core system and extensions and a large repository of example workflows is available facilitating efficient learning of the tool. KNIME also integrates Weka, allows scripting for R and Python and provides commercial extensions for more specific functionalities.

Similar to RapidMiner, Knime provides support for big data analytics. The KNIME Big Data Extension, which provides a set of nodes for accessing Hadoop/HDFS via Hive from inside KNIME can be easily installed and used of the shelf. Still, it lacks the support for direct usage of Mahout and in-memory cluster analytics (like Spark), but this can be expected soon as an update of Big Data extension.

## Materials and Methods

### Data source and experimental environment

The MIMIC II (version 2.6) clinical database consists of 32,536 ICU patients (medical, surgical, coronary care and neonatal), admitted to Beth Israel Deaconess Medical Center (Boston, MA) from 2001 to 2008 [27, 28]. The establishment of the database was approved by the Institutional Review Boards of the Massachusetts Institute of Technology (Cambridge, MA) and Beth Israel Deaconess Medical Center (Boston, MA). The data in the MIMIC-II database is available to other researchers. The contents of the MIMIC-II Clinical Database are derived from original data that contained protected health information (PHI), as defined by HIPAA. The providers of the data have given scrupulous attention to the task of locating and removing all PHI, so that the remaining data can be considered de-identified and therefore not subject to the HIPAA Privacy Rule restrictions on sharing PHI. Because of the richness and detail of the database, the data is released only to legitimate researchers under the terms and conditions as described by Physionet.org [28]. All data and related metadata is available after successful completion of the NIH web-based training course named “Protecting Human Research Participants” enabling access to the entire MIMIC-II database. Accessing the database was approved for authors S.V.P & Z.Z. (certification number: 1712927 & 1132877). Informed consent was waived due to observational nature of the study.

The MIMIC-II clinical database includes data related to patient demographics, hospital admissions & discharge dates, room tracking, death dates (in or out of the hospital), ICD-9 codes, health care providers and types. All dates were surrogate dates but time intervals were preserved. Additionally, physiological data (hourly vital sign metrics, SAPS score, SOFA score, ventilator settings, etc.), medications consumption, laboratory investigations, fluid balance calculations and notes & reports (discharge summary, nursing progress notes, cardiac catheterization, ECG, radiology, and echo reports) were included. The SAPS-I score (Simplified Acute Physiology Score) was calculated using the method outlined earlier [29]. A New Simplified Acute Physiology Score (SAPS II) based on a European/North American Multicenter Study was later published [30].

The SOFA score (Sequential Organ Failure Assessment) was used to assess the incidence of organ dysfunction [31]. The MIMIC-II database contained patients from five ICU types: medical (MICU), surgical (SICU), cardiac (CCU), cardiac surgery recovery (CSRU) and neonatal (NICU).

The initial size of the dataset was identical to the size of the MIMIC-II Clinical Database. All data for a given patient were contained in a set of 33 flat files for each patient. The data archives contained the flat files for about 1000 subjects each. The decompressed flat files occupied about 31 GB in all. The process presented in this paper consisted of only a third of the number of patients from the MIMIC-II database.

**Fig 1:**
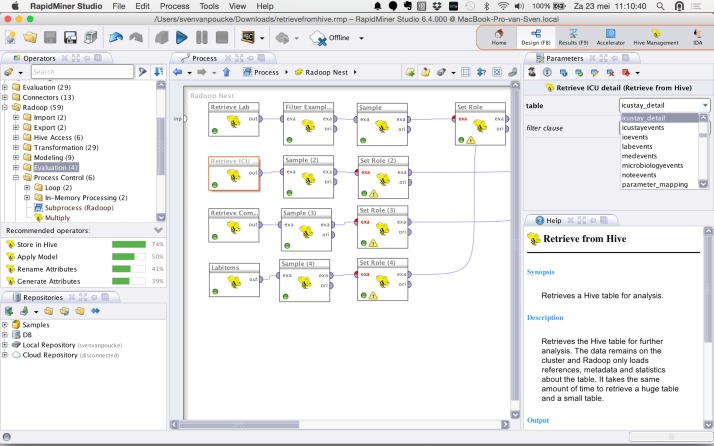
Illustration of integration of the MIMIC-II database in a Hadoop/RapidMiner computer cluster: data retrieval and preprocessing.

In order to enable a scalable environment for future research and to demonstrate seamless usage of big data technologies within a code free environment, the MIMIC-II Clinical Database flat files were integrated in a dedicated Hadoop cluster with Hive server [32, 33] (Fig 1).

RapidMiner 6.5 was installed following the instructions provided by RapidMiner. RapidMiner provided data mining and machine learning procedures visualization, predictive analytics and statistical modeling, evaluation, and deployment [34]. RapidMiner Radoop 2.3.0 was installed through the extension manager of RapidMiner and used to connect to the Hadoop cluster and to perform data loading and transformation (Extract, Transform, Load (ETL) and initial preprocessing. RapidMiner Radoop pushed down visually designed workflows for analytics into Hadoop environments for processing the workflows–integrating with core Hadoop technologies HDFS, MapReduce/YARN and Hive. The MIMIC-II database was initially imported in a PostgreSQL database and consequently converted to Hive. For the implementation of security, a 4-layer security model for Hadoop was used. The first level is responsible for authenticating a user (Perimeter Security). The second level is responsible for authorizing access to data (Data Access Security), i.e. granting access to users only to data, services and resources that they are specifically entitled to use. The common goal of the third security level is to foster accountability by allowing administrators to monitor and audit data access on Hadoop. The fourth level of security covers data-at-rest encryption, on-the-wire encryption, data masking, etc. The hardware used in this research consisted of a local machine (MacBookPro 11.1; Intel Core i5; 2,4 GHz; 1 processor, 2 cores; L2-cache (per core): 256 KB; L3-cache: 3 MB Memory 8 GB) and a Hadoop cluster (5 nodes; 8GB/node; version 2.4.1; configured capacity 7.1 TB)

In the following text we present the algorithms used for the analysis. First we focus on the classification algorithms, then feature selection algorithms are covered.

### Predictive algorithms

**Naive Bayes (NB)—**The Naive Bayesian learning uses Bayes theorem with “Naive” assumption of independence between predictors [35]. Examples are classified based on the posterior probability that an example should be assigned to class.

$$y=\underset{k\in \{1..K\}}{\text{arg max}}\;P(C_{k})\prod_{i=1}^{n}p(x_{i}|C_{i})$$

Even though, independence assumption is violated in most real world applications, Naive Bayes often demonstrated satisfactory performance in practice [36], and was classified as one of the top 10 algorithms in data mining [37]. Additionally, Naive Bayes are easy to construct, without any setting or adjusting of complex parameters, computational and time effectiveness. Naive Bayes have the ability to work with large datasets (big data) and provide good interpretability, which is a must in real world bio-medical applications [38].

**Decision trees (DT)** are predictive algorithms based on “greedy”, top-down recursively partitioning of data. DT algorithms perform an exhaustive search over all possible splits in every recursive step. The attribute (predictor) demonstrating the best split by some evaluation measure is selected for branching the tree. Regularly used are information theoretic measures (e.g. Information Gain, Gain Ratio, Gini etc.) or statistical tests quantifying significance of association between predictors and class. The procedure is recursively iterated, until a stop criterion is met. Greedy strategy for DT building is often criticized, but the ability to build strait forward and the highly interpretable rules on massive data, led to many successful applications in medical applications [39,40]. In this research we used J48 algorithms which is the Java implementation of the C4.5 algorithm [41].

**Logistic regression (LR)** is a widely used linear classifier modeling the probability of a dependent binary variable *y* given a vector of independent variables *X*. For the estimation of the probability the example belongs to the positive class, a logit model is used:

$$\text{log}(\frac{p}{1-p})=\theta_{0}+\theta_{1}x_{1}+\ldots +\theta_{n}x_{n}$$

where *p* presents probability that *y* = 1, θ_j_, where *j* = *1*,*…*,*n* present the weights of the corresponding dependent variable, while *p/(1-p)* is called the odds ratio, Parameters θ_j_, where *j* = *1*,*…*,*n* of the model can be interpreted as changes in log odds or the results can be interpreted in terms of probabilities [42]. Interpretability, scalability and good predictive performance made logistic regression a widely used classifier in the medical domain [43–45].

**Support Vector Machines (SVMs)** construct hyper-planes between the examples (represented as points in high-dimensional space), in such a way that examples of the separate categories are divided by a clear gap that is as wide as possible [46, 47]. New examples are subsequently mapped into the same space and predicted to belong to a category based on the side of the gap they fall in. SVMs are considered as one of the state-of-the-art classification models, with the ability to handle large feature spaces and to avoid over-fitting. Still, unlike NB, DTs and LR they are not widely used in medical research because of the lack of interpretability (examples represented in highly dimensional feature spaces), but also because of their model-fitting nature, where hyper-parameters have to be optimized without clear theoretical guidance.

In this research we used a radial basis function (rbf) and linear (lin) SVM models. In case of rbf- SVM, two parameters are crucial to quantify the performance of a classifier: the soft-margin penalty or cost (C) represents the amount of errors allowed during the training and evaluation steps and the γ (gamma) representing the width of the SVM radial function.

**Ensemble (meta-learning) methods** combine multiple models in order to provide more accurate or more stable predictions. Ensemble models can aggregate the same model that is built on different sub-samples of data, different models built on the same sample or a combination of the previous two techniques. Ensemble methods are often used to improve the individual performance of algorithms that constitute ensembles [48] by exploiting the diversity among the models produced. Next a short explanation of the ensemble methods used in this paper is provided: Random Forest [49], Boosting [50], Bootstrap Aggregating (Bagging) [51] and Stacking [52].

**Random Forest (RF)** is an ensemble classifier that evaluates multiple decision trees and aggregates their results, by majority voting, in order to classify an example [49]. There is a two level randomization in building these models. First, each tree is trained on a bootstrap sample of the training data and second, in each recursive iteration of building a DT (splitting data based on information potential of features), a subset of features for evaluation is randomly selected. This strategy allows efficient model building, and despite its random nature often provides highly accurate predictive models. In this research we grew and evaluated Random Forest (RF) with 10 trees (with the default parameters of Weka’s implementation of Random Forest).

**Boosting** is an ensemble meta-algorithm developed in order to improve supervised learning performance of weak learners (models whose predictive performance is only slightly better than random guessing). Boosting algorithms are built on a principle that subsequent classifiers are adapted to improve predictive performance on those instances that are misclassified by previous classifiers. In this study, the Adaptive Boosting (AdaBoost) algorithm was used [50]. The AdaBoost algorithm builds and applies multiple classifiers over a user defined number of iterations. In each iteration, the weights of each incorrectly classified example by the previous classifier are increased, and the weights of each correctly classified example are decreased in order the new classifier is adapted to the data that is misclassified by previous ones.

**Bagging** algorithm builds and applies a series of models that are built on different data sub-samples (with replacement) from the initial dataset, and apply, for example, a tree classifier (e.g., CHAID) to the successive samples [51]. In practice, this method is often used in order to address the instability of models often the case in small data sets. The final prediction of an ensemble could be derived by simple or weighted voting.

**Stacking (Stacked generalization)** builds several different models (in contrast to bagging that builds one type of model on different subsamples) [52]. Similar to Boosting, the final decision is made by simple or weighted voting. In this research we used the J48 algorithm and Naive Bayes as basis for building a stacked classifier.

### Feature weighting and selection

In this study we evaluated several Filter and Wrapper feature selection schemes. **Filter Selection (FS)** methods rely on the evaluation of the information potential of each input feature in relation to the label. Filter Selection methods are very fast and scalable, but since they are not relating a feature subset selection with the algorithm performance, they can underperform when applied for specific predictive algorithms. Additionally, most of these techniques are based on weighting (providing a list of weights on output). Consequently, a threshold search and selection of the “right” number of features is needed. In this study, several schemes for filter feature weighting and selection were implemented. The first is based upon **Correlation** returning the absolute or squared value of the correlation as attribute weight. Furthermore we applied **Information Gain** and **Gini index**, two weighting schemes that are based on information theoretic measures, frequently used with decision trees for evaluation of potential splits [41]. The **T-test** calculated, for each attribute, a p-value for 2-sided, 2-sample t-Test. Finally, the **ReliefF** evaluated the impact of an attribute by repeatedly sampling an instance and considering the value of the given attribute for the nearest instance of the same and different class [53].

Instead of relying on the evaluation of each feature independently (as is the case in filter feature selection methods), **Wrapper** methods evaluate the usefulness of feature sets based on the predictive performance of the algorithms. This means that they provide a better estimation of the final model performance [54]. Wrappers are much more computationally expensive than filter techniques (since they build and evaluate the entire predictive model in each iteration). This is the reason wrappers are usually not used in association with computationally demanding models such as SVMs.

In this study we used two popular and diverse strategies from this class **Forward Selection and Backward elimination** [55]. The Forward Selection operator starts with an empty selection of attributes and, in each round, it adds each unused attribute and evaluates it based on algorithm performance. Only the attribute giving the highest increase of performance is added to the selection. Then a new round is started with the modified selection. **Backward elimination** is based on the same strategy, but in opposite direction. It starts with the full set of attributes and, in each round, it removes each remaining attribute of the given ExampleSet.

**Evolutionary Search (ES) for parameter optimization:** is a generalization of a genetic algorithm inspired by the adaptation of many species to new-come problems. This adaptation searches for adequate solutions over a huge number of genotypes [56]. It is considered population-based, meta-heuristic using mechanisms inspired by biological evolution, such as reproduction, mutation, recombination, and selection. Solutions of the optimization problem, called candidates, represents individuals of a population, and the fitness function determines the quality of the solutions. Evolutionary algorithms often perform well in various types of optimization problems because they do not make any assumption about the underlying search space. Because of prior mentioned advantages and successful applications in many areas, Evolutionary Search (ES) is often used for SVM parameter optimization [57]. Basic parameters are: number of units in population, number of generations, crossover and selection scheme. As mentioned before, for the purpose of this study, ES for SVM parameter optimization was used (with default values provided by RapidMiner).

## Experiments and Results

In this section, the process of data extraction, pre-processing and exploratory analysis are described. Furthermore, RapidMiner processes for automatic building of multiple predictive models, parameter optimization and feature selection are illustrated. Finally the results of the feature selection and classification models are evaluated.

### Data extraction, pre-processing and exploratory analyses

Guided by the CRoss-Industry Standard Process for Data Mining (CRISP-DM), the ETL process started by retrieving data from the MIMIC-II tables of interest [34]. The database was accessed within RapidMiner by a running connection to the Hive server. The relational database of MIMIC-II consisted of of 38 tables. In this pilot, data was extracted from the following tables: LABEVENTS,D_LABITEMS, COMORBIDITY_SCORES, ICUSTAY_DETAIL resulting in 3 data sets (Platelet Count, ICUdetail, Comorbidity) (Fig 1). Considering the entire database as baseline, the initial query reduced the data to a selection of 11944 admissions. For this purpose, RapidMiner Radoop was used for extracting the data from the cluster.

Attribute roles were defined (id, regular, label, etc) and example sets were joined using id attributes as key. The dataset used for modeling and feature weighting consisted of 70 attributes with icustay_expire_flg as label (Table 1).

**Table 1:** Attributes selected for modeling and feature selection (weighting).

Attributes (alphabetical order)
aids
alcohol_abuse
blood_loss_anemia
cardiac_arrhythmias
chronic_pulmonary
coagulopathy
congestive_heart_failure
deficiency_anemias
depression
diabetes_complicated
diabetes_uncomplicated
drug_abuse
fluid_electrolyte
gender = F
gender = M
height
hypertension
hypothyroidism
icustay_first_careunit = CCU
icustay_first_careunit = CSRU
icustay_first_careunit = FICU
icustay_first_careunit = MICU
icustay_first_careunit = NICU
icustay_first_careunit = SICU
icustay_first_service = CCU
icustay_first_service = CSRU
icustay_first_service = FICU
icustay_first_service = MICU
icustay_first_service = NICU
icustay_first_service = SICU
icustay_last_careunit = CCU
icustay_last_careunit = CSRU
icustay_last_careunit = FICU
icustay_last_careunit = MICU
icustay_last_careunit = NICU
icustay_last_careunit = SICU
icustay_last_service = CCU
icustay_last_service = CSRU
icustay_last_service = FICU
icustay_last_service = MICU
icustay_last_service = NICU
icustay_last_service = SICU
liver_disease
lymphoma
metastatic_cancer
obesity
other_neurological
paralysis
peptic_ulcer
peripheral_vascular
PLT0
PLTmax
PLTmean
PLTmin
psychoses
pulmonary_circulation
renal_failure
rheumatoid_arthritis
sapsi_first
sapsi_max
sapsi_min
sofa_first
sofa_max
sofa_min
solid_tumor
valvular_disease
weight_first
weight_loss
weight_max
weight_min

All data was filtered returning a data set including rows that fulfilled a predefined condition (ICU admission age = adult; laboratory test: platelet count (itemid = 50428)) (Fig 1). Platelet count values were mapped to classes according to the following thresholds: normal platelet count 150–450 x10^9^/L; mild, moderate, severe and extreme thrombocythemia, respectively for platelet count values: 450–700 x10^9^/L, 700–900 x10^9^/L, 900–1000 x10^9^/L, >1000 x10^9^/L; grade 1–4 thrombocytopenia 150–75 x10^9^/L, 75–50 x10^9^/L, 50–25 x10^9^/L and <25 x10^9^/L.

A total of 11944 ICU admissions satisfied our inclusion criteria. From the 11944 patients (age >15 years) admitted to ICU, ICU mortality was 11.5% (n = 1378). Patients who survived ICU stay were significantly younger (63.9±18.4 years vs 70.3±16.2 years; p<0.001) and and significantly more male patients survived ICU stay (57.0% vs 50.7%; p<0.0001). SAPS-1 and SOFA scores were significantly higher in the non-survivor group, respectively (13.9± 4.7 vs 19.3±5.6 and 5.4±3.4 vs 9.6±4.5) (Table 2).

**Table 2:** Characteristics of intensive care units survivors and non-survivors.

Characteristics	Population (n = 11944)	Survivors (n = 10566)	Non-survivors (n = 1378)	p
Age (years)	63.2±18.6	63.9±18.4	70.3±16.2	<0.0001
Sex (male, %)		6025 (57.0%)	578 (50.7%)	<0.0001
SAPS-1 on admission	14.7±4.5	13.9±4.7	19.3±5.6	<0.0001
SOFA on admission	6.0±3.6	5.4±3.4	9.6±4.5	<0.0001
**Comorbidity (n, %)**				
Congestive heart failure		3263 (30.9%)	420 (30.5%)	= 0.766
Paralysis		115 (1.1%)	13 (0.9%)	= 0.672
Renal failure		1738 (16.4%)	131 (9.5%)	<0.0001
Uncomplicated diabetes		2258 (21.4%)	258 (13.7%)	= 0.015
Complicated diabetes		2658 (25.2%)	59 (4,2%)	<0.0001
Coagulopathy		765 (7.2%)	144 (10.4%)	<0.0001
AIDS		80 (0.8%)	9 (0.7%)	= 0.667
Chronic pulmonary disease		2437 (23.1%)	262 (19,0%)	= 0.008
Obesity		92 (0.9%)	8 (0.6%)	= 0.269
Liver disease		471 (4.5%)	103 (7.5%)	<0.0001
**Types of care unit (n, %)**				
CCU		2054(19.4%)	304 (22.1%)	= 0.751
CSRU		2804 (26.5%)	306 (22.2%)	= 0.02
MICU		4660 (44.1%)	528 (38.3%)	<0.0001
SICU		181 (1.7%)	56 (4.1%)	<0.0001
**Platelet count (x 10E9/L)**				
PLT0	255.2±127.2	257.4±126.4	238,2±132.4	<0.0001
PLTmax	454.9±228.6	469.3±227.4	344.1±206.5	<0.0001
PLTmin	122.9±84.8	122.4±83.9	126.9±91.7	= 0.666
PLTmean	245.5±112.9	249.8±111.6	212.4±117.0	<0.0001

In the non-survivor group, significantly more patients suffered from renal failure, complicated diabetes, coagulopathy and liver disease. Mortality was higher for patients admitted on MICU and SICU. The prevalence of normal platelet count on ICU admission was 73.8%. Low platelet count was observed in 12.5%, 2.3%, 1.2%, 0.6% of the cases on admission, respectively for grade 1, 2, 3 and 4 thrombocytopenia. High platelet count was observed in 9.0%, 0.3%, 0.0%, 0.3% of the cases on admission respectively for mild, moderate, severe and extreme thrombocythemia. Mean platelet count on admission was 255.2±127.2 x10^9^/L. PLT_0_ was significantly lower in non-survivors than in survivors (238,2±132.4x10^9^/L; 257.4±126.4 x10^9^/L, p<0.001). Maximum and mean platelet count during ICU stay were significantly lower in non-survivors vs survivors, respectively 344.1±206.5x10^9^/L vs 469.3±227.4x10^9^/L and 212.4±117.0x10^9^/L vs 249.8±111.6x10^9^/L. In the survivors, 74.4% of the patients had a normal platelet count. Low platelet count was observed in 11.9%, 2.2%, 1.0%, 0.5% of the cases on admission, respectively for grade 1, 2, 3 and 4 thrombocytopenia. High platelet count was observed in 9.4%, 0.3%, 0.0%, 0.3% of the cases on admission respectively for mild, moderate, severe and extreme thrombocythemia. The non-survivors, 68.6% of the patients resulted in a normal platelet count. Low platelet count was observed in 16.6%, 3.5%, 3.0%, 1.6% of the cases on admission, respectively for grade 1, 2, 3 and 4 thrombocytopenia. High platelet count was observed in 6.4%, 0.6%, 0.0%, 0.0% of the cases on admission respectively for mild, moderate, severe and extreme thrombocythemia.

### Automatic model building, parameter optimization and evaluation

**Fig 2:**
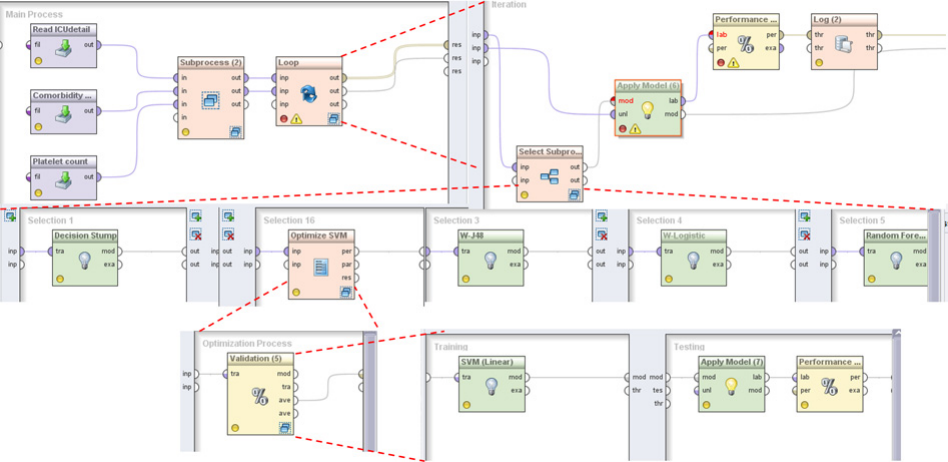
Basic process for automatic building, parameter optimization and evaluation of multiple predictive models as displayed in RapidMiner.

The process for automatic building, parameter optimization and evaluation of multiple predictive models is illustrated in Fig 2.

The upper left part of the figure illustrates the main process. In the main process, the data (Fig 1) was extracted from the 3 data sets (Platelet count, Comorbidity, ICUdetail) and basic pre-processing was performed in a *Sub-process* operator. The final operator in the main process (*Loop*) was defined by a global variable (macro) iterating over a user defined interval. This allowed looping through multiple algorithms resulting in model building and evaluation in a single process execution. The inner operators of the *Loop* consisted of a *Select Subprocess*, *Performance* and *Log*. The *Apply model* took as an input the model provided from the *Select Sub-process* and holdout data that were forwarded from a previous process level.

When the model was applied, AUPRC (Area Under the Precision Recall Curve) values were calculated. Since currently RapidMiner does not provide AUPRC calculation within its *Performance* operator, we used the RapidMiner-R extension allowing incorporation of R scripts within the Execute-R operator, based on the PRROC R package [58]. In order to enable building and evaluation of multiple models in one process execution, the *Select Sub-process* operator, consisted of an inner operator structure that could be iteratively executed for user defined number of times. For the purpose of this paper, we defined 17 subprocesses, containing one predictive algorithm (middle layer of Fig 2). The execution order is controlled by the iteration macro, provided from the Loop operator.

Additionally, this process structure allowed parameter optimization if needed (e.g. for SVMs). As described before we implemented an Evolutionary algorithm for parameter optimization of SVMs (Fig 2. 2nd sub-process from left, layer 2).

**Fig 3:**
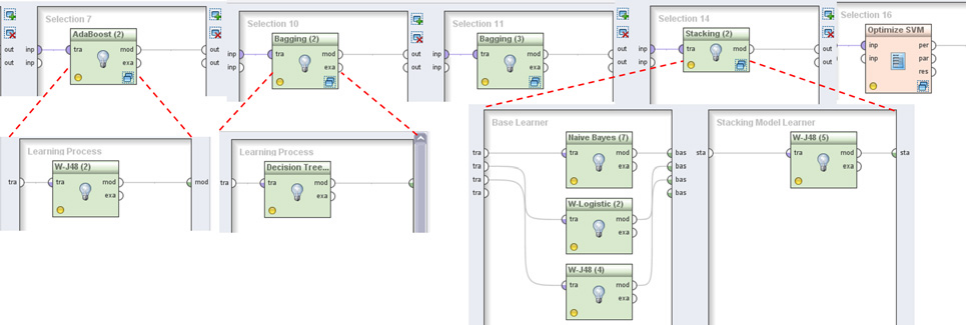
Illustration of the ensemble learning methods as displayed in RapidMiner (Decision Stump, AdaBoost, Random Forest, Bagging, W-J48, Decision Tree, Naive Bayes, Stacking, Logistic Regression, Support Vector Machine).

Additionally to the first process, described in Fig 2, we developed two processes as an extension allowing automatic feature selection, model building and evaluation under various feature selection schemes (described in the Materials and Methods section) (Fig 3).

In case of Wrapper techniques, the only difference with the previously described process is that we used *ForwardSelection* and *BackwardElimination* and embeded the part for model learning and evaluation (*Select Sub-process*, *ApplyModel and Execute R*) into them. On the output this resulted in one “optimal” feature set for each algorithm, which is further evaluated on the test-set.

In case of Filter techniques we wrapped the previously described process in an additional *Loop* operator (allowing automatic learning of each algorithm over each selected feature set). As mentioned before, the problem with Filter selection is the determination of the right (most adequate) threshold of feature weights and selection of number of features. So, for each algorithm and feature selection technique, we evaluated AUPRC performance based on thresholds that select from 5 to 45 attributes (variables) with step of 5 [59, 60]. This was achieved by usage of the *OptimizeParameters* operator with Grid strategy, that evaluated all algorithms in the described range and returned the optimal number of parameters. This process demonstrated the robustness and ease of implementation of a relatively complex experimental setups in RapidMiner (this process automatically executes 672 experiments: 14 algorithms X 6 feature selection schemes X 8 thresholds). Since optimization of parameters for SVM model fitting is computationally very demanding, we excluded them from the experiments with different feature selection techniques (they are evaluated only on the complete set of features). In case rbf-SVM we optimized γ (kernel width) in range between 0.001 to 2 and C between 10^−9^ to 10^5^ as suggested in [61]. In case of linear SVMs, only C is optimized in the same parameter range. As an optimization technique, we used Evolutionary Search for parameter optimization (part of standard RapidMiner package) with 10 generations and 10 population size. All other parameters were fixed to their default values.

### Evaluation

All evaluations reported in this paper are based on holdout (test) set created by stratified sampling meaning that the initial distribution of positive and negative classes of the target attribute are preserved. Experiments including some type of optimization (parameters or feature sets), sensitive for over-fitting and reduced generalization of predictive models, are cross-validated on training sets (70% of initial data). Because of the unbalanced nature of data we calculated AUPRC (Area Under the Precision Recall Curve) values as evaluation parameter for model comparison. Other parameters, frequently published in this respect, such as AUC (Area Under the ROC Curve) and Accuracy are independent of class size ratios, but often provide misleading results in unbalanced data scenarios [59, 60]. AUPRC measured the fraction of negatives misclassified as positives and resulting in a plot representing precision (TP/(TP+FP)) vs. recall ratio (this is TPR, sometimes referred to as sensitivity)(TP: True Positive, FP: False Positive, TPR: True Positive Ratio). By varying the threshold, the precision can be calculated at the threshold that achieves that recall ratio. AUPRC is a less forgiving measure, and a high value indicates that a classification model makes very few mistakes.

### Performance and Feature Selection

All experimental results are summarized in Table 3.

**Table 3:** AUPRC (Area Under the Precision Recall Curve) performance and feature selection.

Algorithm/Feature selection	No Feature selection	Info gain	ReliefF	MRMR	Correlation	Gini	Ttest	Forward selection	Backward elimination
RM—Decision stump	0.275	0.282 (5)	0.282 (5)	0.142 (15)	0.282 (5)	0.282 (5)	0.115 (30)	**0.358 (1)**	0.275 (69)
J4.8	0.448	0.537 (5)	0.484 (5)	0.115 (15)	**0.593 (5)**	**0.593 (5)**	0.115 (15)	0.575 (8)	0.454 (67)
Naïve Bayes	0.435	0.593 (5)	0.553 (10)	0.119 (5)	0.573 (15)	0.569 (5)	0.125 (35)	0.588 (20)	**0.623 (26)**
Logistic regression	0.687	0.607 (5)	0.664 (15)	0.215 (45)	0.629 (5)	0.629 (5)	0.19 (45)	0.664 (32)	**0.692 (57)**
RF-weka	0.743	0.707 (5)	0.732 (15)	0.124 (5)	0.734 (5)	0.734 (5)	0.341 (45)	0.744 (8)	**0.764 (69)**
AdaBoost-J48	**0.668**	0.628 (5)	0.5 (5)	0.129 (5)	0.654 (5)	0.654 (5)	0.127 (5)	0.637 (6)	0.661 (69)
AdaBoost—NB	**0.555**	0.515 (10)	0.519 (20)	0.155 (45)	0.499 (20)	0.475 (10)	0.142 (40)	0.433 (4)	0.534 (68)
AdaBoost—LR	0.432	**0.575 (20)**	0.571 (25)	0.169 (45)	0.565 (15)	0.555 (15)	0.174 (45)	0.384 (16)	0.436 (67)
Bagging-J48	0.494	0.501 (5)	0.511 (10)	0.115 (15)	**0.53 (5)**	**0.530 (5)**	0.115 (10)	0.525 (5)	0.478 (69)
Bagging—NB	0.460	0.592 (5)	0.554 (10)	0.189 (45)	**0.569 (5)**	0.568 (5)	0.146 (45)	0.594 (12)	0.483 (68)
Bagging (LR)	0.681	0.605 (5)	0.66 (15)	0.205 (45)	0.628 (5)	0.628 (5)	0.187 (45)	0.659 (34)	**0.686 (58)**
Stacking (DS, J4.8, NB)	**0.570**	0.486 (30)	0.511 (30)	0.115 (45)	0.51 (20)	0.496 (25)	0.157 (45)	0.327 (3)	0.397 (68)
SVM—linear	0.465								
SVM—rbf	0.588								

AUPRC (Area Under the Precision Recall Curve) performance and the number of features involved in model building (in brackets) are showed for each model (rows) and feature selection technique (columns). In bold, the best AUPRC performances for each row are illustrated, revealing the best feature selection method for each model.

**Fig 4:**
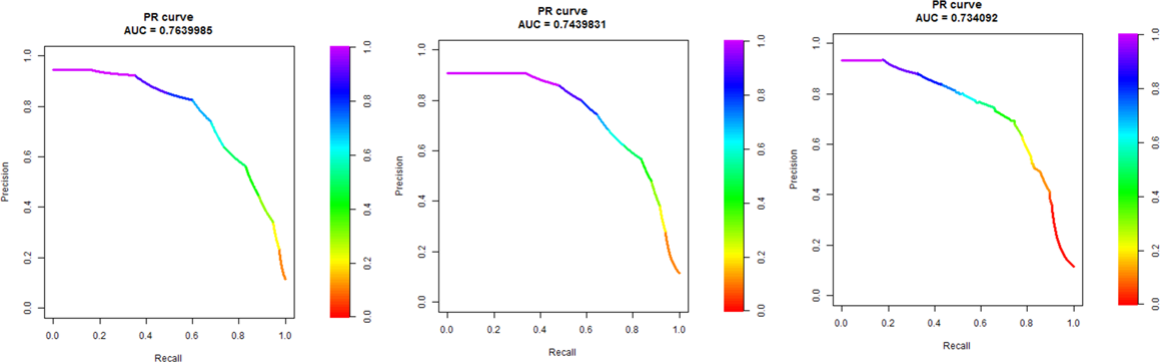
AUPRC curves for the 3 best models. Random Forest (RF) in association with Backward Selection (BS) and 69 features (left), with Forward Selection (FS) and 8 features (middle) and Gini Selection (GS) and 5 features.

AUPRC performance and the number of features involved in model building (in brackets) are showed for each model (rows) and feature selection technique (columns). In bold, the best AUPRC performances for each row are provided, revealing the best feature selection method for each model. Based on these results, the Random Forest (RF) was the dominant modeling technique (provided by the best performance for each feature selection schema and for the entire feature set). The best result in terms of AUPRC (AUPRC = 0.764) was achieved associating Random Forest (RF) and Backward Elimination (BE) technique (Fig 4).

It is interesting to observe that BE removed only one feature (*icustay_first_service = CSRU*) resulting in an increased model performance from 0.743 to 0.764. Further inspection of the results revealed that all other feature selection techniques put this feature above the 30th rank (in a total of 70 features). Furthermore, it should be noticed that by using Random Forest, there are three other, valuable feature selection techniques with respect to AUPRC values and the number of features. Namely, in association with Forward selection (FS), Random Forest (RF) resulted in AUPRC values of 0.744 (with 8 features). Random Forest in association with Correlation and Gini Selection resulted in AUPRC values of 0.734 with only 5 features. (*sofa_max*, *sofa_min*, *sapsi_max*, *sofa_first*, *sapsi_min*). From a medical perspective, the selection of these 5 features is no surprise because SOFA and SAPS values, as severity of disease classification, are intrinsic related to other parameters [30, 31]. Forward Selection resulted in similar features (except for *sapsi_max*) but added *diabetes_uncomplicated*, *icustay_first_careunit = CSRU*, *weight_min* and *PLTmean* to its selection.

Precision (the number of selected attributes that are relevant) and recall (the number of relevant attributes that are selected) have an inverse relationship providing the option to increase one at the cost of reducing the other (Fig 4).

In terms of feature selection, all methods provided similar performance over the models, except for the t-test resulting in a maximum AUPRC in association with Random Forest (RF) of 0.341 with all others values below 0.19.

Closer inspection of the 5 most important features based on the filter selection methods, the *sofa_max score* was **ranked first** (3 times) and **ranked second** (once). Only the t-test method provided a very low ranking for the sofa_max score (63rd rank). As already mentioned, this test resulted in an overall poor performance. *Congestive_heart_failure* and *diabetes_complicated* were also first ranked once (*congestive_heart_failure* was also ranked on a 4th place once). On **second** place, popularly ranked were *sofa min* (3 times), *sofa_max*, *hypertension*, on the **third** place: *hypertension* (once), *sapsi_max*, *icustay_last_service = FICU*, *sapsi_max* (3 times), on the **fourth** place: *icu_stay_lastservice = CCU*, *sofa first* (twice), *weight_first* and finally on **fifth** place: *chronic_pulmonary*, *reumathoid_arthritis*, *sapsi_min* (twice) and *weight max*.

Since this paper identified patients based on survival, it is preferable for the determination of thresholds, to keep recall high (Fig 4), taking in to account not to make most of the predictions false alarms. Based on our results, with recall values on > = 0.8 the model providing the best precision values for that recall value could be recommended.

## Discussion

This pilot study, is the first report presenting the integration of the MIMIC-II database in RapidMiner data mining technology (Fig 1) and demonstrated the modalities of a code free environment for building complex, automated processes in scalable environment.

Data selection from the MIMIC-II database resulted in 70 attributes which covered basically the features previously validated as relevant in relation to outcome survival on ICU. Moreover, platelet counts were added to this selection. Based on the modeling and feature selection processes, the associating of Random Forest (RF) and Backward Elimination (BE) resulted in the best AUPRC values. Additionally, the combination of Random Forest (RF) with Correlation, Gini Selection and Forward Selection resulted in higher AUPRC values. It was no coincidence finding SAPS and SOFA scores highly ranked as they were already validated as disease severity scores. Interestingly, Random Forest (RF) and Forward Selection also retained PLT_mean_ in its selection. In the future, a similar modeling and feature selection procedure could be used implementing features with no, unknown or minimal interdependency. This technique could then provide medical and financial liability for the use of existing and the implementation of new features resulting from laboratory tests or monitoring devices. Additionally, this approach could provide more understanding how important certain features are in relation to the clinical context they are used in.

The presented deployment offered an access to all MIMIC-II data, also for the non-coding scientist community. Our results demonstrated the predictive value of platelet count in the survival of ICU patients. Additionally, changing data input by selecting data from tables of interest and changing modeling and feature selection operators result in other survival predictions.

Although there are other clinical databases available for research in critical care medicine, the MIMIC-II and recently the MIMIC-III database, is currently one of the largest clinical databases able to provide high resolution clinical information [61]. Recently, the MIMIC-III database is introduced with an update of patient data and a more efficient database architecture.

The advantage of using this type of databases is the representation of “real world” setting in which no strict study protocol has been performed in collecting data. Indeed, many interventional trials have been criticized for its strict inclusion and exclusion criteria [61]. Although a web-based QueryBuilder and Virtual Machine image of the MIMIC-II database are available, with clinical researchers rarely achieving the required expertise in SQL [62], the database is underemployed. The RapidMiner platform has a code-free UI and is available both in the cloud and as an open-source client/server platform. With a platform including more than 1,500 methods across all stages of the predictive analytics life cycle, RapidMiner has the breadth and flexibility adapted to the researchers’ need to consume data. RapidMiner helps reduce time-to-insights and guide best practices for data analysts, analyzing the behavior of their users to create “wisdom of the crowds” guidance. A report by Gartner, reviewed 16 analytics and data science firms over 10 criteria, based on completeness of vision and ability to execute data [8]. SAS, IBM, KNIME, and RapidMiner lead in Gartner 2015 Magic Quadrant for Advanced Analytics Platforms.

Data mining on ICU-patient data opens opportunities enabling modeling with classification, evaluating predictive accuracy of models, visualizing the performance of models using lift charts and ROC charts, and finally ranking patients by the predicted confidence for a clinical decision in order to select the best candidates for a therapy.

Several limitations need to be acknowledged. First, the study is retrospective and bears potential limitations of such design. For instance, patients without platelet count measured during ICU stay were excluded from the analysis, this may be responsible for bias because the included cohort does not represent the whole study population. However, included and excluded cohorts are similar in many clinical characteristics (data not shown), making our cohort representative of the target population. Second, ICU patients were heterogeneous including medical, surgical and cardiac surgical patients, whether narrowing study population will improve the prognostic value of platelet count for survival requires further investigations. Third, although every effort has been made to adjust for the confounding factors by using multivariate analysis, other unknown factors may still exist to confound the prognostic value of platelet count. The authors used ICU mortality instead of the more commonly used ones such as 28-day and 90-day mortality as the study endpoint. This is because data after ICU discharge are not directly available in the MIMIC-II database [63].

## Conclusion

This paper reports the integration of the MIMIC-II database in RapidMiner, enabling scalable predictive analysis of clinical data from critically ill patients in a visual, code free environment. The major benefit from the proposed integration is seamless manipulation, data extraction, preprocessing and predictive analytics of huge amounts of data, using visual tools without the need for writing a single line of code. As such this system has the potential to put cutting edge analytical tools into the hands of medical domain experts, eventually bridging the gap between potential and actual usage of medical data. This approach enables the development of Digital Research Environments (DRE) including data lakes (large object-based storage repositories that holds data in its native format until it is needed) becoming attractive platforms in research facilities around the world. As a showcase of the proposed environment we demonstrated a prognostic value of platelet count in critically ill patients, by defining several processes for automatic building of multiple models, parameter optimization, feature selection and model evaluation. These processes are robust and flexible enough to provide fast and effective research in a variety of clinical research questions with minimal adoptions. The authors did not have the intention to provide any clinical relevance of the value of platelet counts on admission in relation to survival on ICU.

## Supporting Information

S1 File Extensible Markup Language (XML) file of the process (data retrieval and preprocessing) as presented in this paper. Copy and paste this code into the RapidMiner XML view (remove the previous xml code), click the green check symbol and switch back to the normal Process (diagram) view.
(DOCX)

S2 File Extensible Markup Language (XML) file of the process (modeling and feature selection) as presented in this paper. Copy and paste this code into the RapidMiner XML view (remove the previous xml code), click the green check symbol and switch back to the normal Process (diagram) view.
(DOCX)
