# Supplementary material for: Scalable Predictive Analysis in Critically Ill Patients Using a Visual Open Data Analysis Platform
Source: PLoS One. 2016 Jan 5;11(1):e0145791. doi: 10.1371/journal.pone.0145791 (PMC4701479; doi:10.1371/journal.pone.0145791)
Supplement: S1 File — Copy and paste this code into the RapidMiner XML view (remove the previous xml code), click the green check symbol and switch back to the normal Process (diagram) view. (DOCX) [file pone.0145791.s001.docx]

**Supporting Information:**

**S1 File:**

Extensible Markup Language (XML) file of the process (data retrieval and preprocessing) as presented in this paper. Copy and paste this code into the RapidMiner XML view (remove the previous xml code), click the green check symbol and switch back to the normal Process (diagram) view.

<?xml version="1.0" encoding="UTF-8" standalone="no"?>

<process version="6.5.002">

<context>

<input/>

<output/>

<macros>

<macro>

<key>basepath</key>

<value>C:\Users\Martin\.RapidMiner\repositories\MIMIC II\</value>

</macro>

</macros>

</context>

<operator activated="true" class="process" compatibility="6.0.002" expanded="true" name="Process">

<parameter key="resultfile" value="/Users/svenvanpoucke/Documents/BigData2015/ArtikelMaud/MIMICplateletsMartin.res"/>

<parameter key="random_seed" value="2001"/>

<process expanded="true">

<operator activated="true" class="read_csv" compatibility="6.5.002" expanded="true" height="60" name="Read ICUdetail" width="90" x="45" y="120">

<parameter key="csv_file" value="/Users/svenvanpoucke/Documents/BigData2015/ArtikelMaud/icudetail"/>

<parameter key="first_row_as_names" value="false"/>

<list key="annotations">

<parameter key="0" value="Name"/>

</list>

<parameter key="encoding" value="US-ASCII"/>

<list key="data_set_meta_data_information">

<parameter key="0" value="icustay_id.true.integer.attribute"/>

<parameter key="1" value="subject_id.true.integer.id"/>

<parameter key="2" value="gender.true.polynominal.attribute"/>

<parameter key="3" value="dob.true.polynominal.attribute"/>

<parameter key="4" value="dod.true.polynominal.attribute"/>

<parameter key="5" value="expire_flg.true.polynominal.attribute"/>

<parameter key="6" value="subject_icustay_total_num.true.integer.attribute"/>

<parameter key="7" value="subject_icustay_seq.true.integer.attribute"/>

<parameter key="8" value="hadm_id.true.integer.attribute"/>

<parameter key="9" value="hospital_total_num.true.integer.attribute"/>

<parameter key="10" value="hospital_seq.true.integer.attribute"/>

<parameter key="11" value="hospital_first_flg.true.polynominal.attribute"/>

<parameter key="12" value="hospital_last_flg.true.polynominal.attribute"/>

<parameter key="13" value="hospital_admit_dt.true.polynominal.attribute"/>

<parameter key="14" value="hospital_disch_dt.true.polynominal.attribute"/>

<parameter key="15" value="hospital_los.true.integer.attribute"/>

<parameter key="16" value="hospital_expire_flg.true.polynominal.attribute"/>

<parameter key="17" value="icustay_total_num.true.integer.attribute"/>

<parameter key="18" value="icustay_seq.true.integer.attribute"/>

<parameter key="19" value="icustay_first_flg.true.polynominal.attribute"/>

<parameter key="20" value="icustay_last_flg.true.polynominal.attribute"/>

<parameter key="21" value="icustay_intime.true.polynominal.attribute"/>

<parameter key="22" value="icustay_outtime.true.polynominal.attribute"/>

<parameter key="23" value="icustay_admit_age.true.real.attribute"/>

<parameter key="24" value="icustay_age_group.true.polynominal.attribute"/>

<parameter key="25" value="icustay_los.true.integer.attribute"/>

<parameter key="26" value="icustay_expire_flg.true.polynominal.label"/>

<parameter key="27" value="icustay_first_careunit.true.polynominal.attribute"/>

<parameter key="28" value="icustay_last_careunit.true.polynominal.attribute"/>

<parameter key="29" value="icustay_first_service.true.polynominal.attribute"/>

<parameter key="30" value="icustay_last_service.true.polynominal.attribute"/>

<parameter key="31" value="height.true.real.attribute"/>

<parameter key="32" value="weight_first.true.real.attribute"/>

<parameter key="33" value="weight_min.true.real.attribute"/>

<parameter key="34" value="weight_max.true.real.attribute"/>

<parameter key="35" value="sapsi_first.true.integer.attribute"/>

<parameter key="36" value="sapsi_min.true.integer.attribute"/>

<parameter key="37" value="sapsi_max.true.integer.attribute"/>

<parameter key="38" value="sofa_first.true.integer.attribute"/>

<parameter key="39" value="sofa_min.true.integer.attribute"/>

<parameter key="40" value="sofa_max.true.integer.attribute"/>

</list>

</operator>

<operator activated="true" class="read_csv" compatibility="6.5.002" expanded="true" height="60" name="Platelet count" width="90" x="45" y="300">

<parameter key="csv_file" value="/Users/svenvanpoucke/Documents/BigData2015/ArtikelMaud/Plateletmeanmaxmin0.csv"/>

<parameter key="date_format" value="YYYY-MM-DD hh:mm"/>

<parameter key="first_row_as_names" value="false"/>

<list key="annotations">

<parameter key="0" value="Name"/>

</list>

<parameter key="encoding" value="US-ASCII"/>

<list key="data_set_meta_data_information">

<parameter key="0" value="Subject_ID.true.integer.id"/>

<parameter key="1" value="PLTmean.true.integer.attribute"/>

<parameter key="2" value="PLTmin.true.integer.attribute"/>

<parameter key="3" value="PLTmax.true.integer.attribute"/>

<parameter key="4" value="PLT0.true.integer.attribute"/>

</list>

</operator>

<operator activated="true" class="read_csv" compatibility="6.5.002" expanded="true" height="60" name="Comorbidity CSV " width="90" x="45" y="210">

<parameter key="csv_file" value="/Users/svenvanpoucke/Documents/BigData2015/ArtikelMaud/comorbidity"/>

<parameter key="first_row_as_names" value="false"/>

<list key="annotations">

<parameter key="0" value="Name"/>

</list>

<parameter key="encoding" value="US-ASCII"/>

<list key="data_set_meta_data_information">

<parameter key="0" value="subject_id.true.integer.id"/>

<parameter key="1" value="hadm_id.true.integer.attribute"/>

<parameter key="2" value="category.true.polynominal.attribute"/>

<parameter key="3" value="congestive_heart_failure.true.binominal.attribute"/>

<parameter key="4" value="cardiac_arrhythmias.true.binominal.attribute"/>

<parameter key="5" value="valvular_disease.true.binominal.attribute"/>

<parameter key="6" value="pulmonary_circulation.true.binominal.attribute"/>

<parameter key="7" value="peripheral_vascular.true.binominal.attribute"/>

<parameter key="8" value="hypertension.true.binominal.attribute"/>

<parameter key="9" value="paralysis.true.binominal.attribute"/>

<parameter key="10" value="other_neurological.true.binominal.attribute"/>

<parameter key="11" value="chronic_pulmonary.true.binominal.attribute"/>

<parameter key="12" value="diabetes_uncomplicated.true.binominal.attribute"/>

<parameter key="13" value="diabetes_complicated.true.binominal.attribute"/>

<parameter key="14" value="hypothyroidism.true.binominal.attribute"/>

<parameter key="15" value="renal_failure.true.binominal.attribute"/>

<parameter key="16" value="liver_disease.true.binominal.attribute"/>

<parameter key="17" value="peptic_ulcer.true.binominal.attribute"/>

<parameter key="18" value="aids.true.binominal.attribute"/>

<parameter key="19" value="lymphoma.true.binominal.attribute"/>

<parameter key="20" value="metastatic_cancer.true.binominal.attribute"/>

<parameter key="21" value="solid_tumor.true.binominal.attribute"/>

<parameter key="22" value="rheumatoid_arthritis.true.binominal.attribute"/>

<parameter key="23" value="coagulopathy.true.binominal.attribute"/>

<parameter key="24" value="obesity.true.binominal.attribute"/>

<parameter key="25" value="weight_loss.true.binominal.attribute"/>

<parameter key="26" value="fluid_electrolyte.true.binominal.attribute"/>

<parameter key="27" value="blood_loss_anemia.true.binominal.attribute"/>

<parameter key="28" value="deficiency_anemias.true.binominal.attribute"/>

<parameter key="29" value="alcohol_abuse.true.binominal.attribute"/>

<parameter key="30" value="drug_abuse.true.binominal.attribute"/>

<parameter key="31" value="psychoses.true.binominal.attribute"/>

<parameter key="32" value="depression.true.binominal.attribute"/>

</list>

</operator>

<operator activated="true" class="join" compatibility="6.5.002" expanded="true" height="76" name="Join" width="90" x="179" y="165">

<list key="key_attributes"/>

</operator>

<operator activated="true" class="subprocess" compatibility="6.5.002" expanded="true" height="76" name="Age (Dod-Dob)" width="90" x="312" y="165">

<process expanded="true">

<operator activated="true" class="set_role" compatibility="6.5.002" expanded="true" height="76" name="Set Role" width="90" x="45" y="120">

<parameter key="attribute_name" value="icustay_id"/>

<parameter key="target_role" value="id"/>

<list key="set_additional_roles">

<parameter key="icustay_expire_flg" value="label"/>

</list>

</operator>

<operator activated="true" class="guess_types" compatibility="6.5.002" expanded="true" height="76" name="Guess Types" width="90" x="179" y="120">

<parameter key="attribute_filter_type" value="subset"/>

<parameter key="attributes" value="dob|dod|hospital_disch_dt|hospital_admit_dt|icustay_intime|icustay_outtime"/>

</operator>

<operator activated="true" class="nominal_to_date" compatibility="6.5.002" expanded="true" height="76" name="Nominal to Date" width="90" x="313" y="120">

<parameter key="attribute_name" value="dob"/>

<parameter key="date_format" value="YYYY-MM-DD HH:MM:SS"/>

</operator>

<operator activated="true" class="nominal_to_date" compatibility="6.5.002" expanded="true" height="76" name="Nominal to Date (2)" width="90" x="447" y="120">

<parameter key="attribute_name" value="dod"/>

<parameter key="date_format" value="YYYY-MM-DD HH:MM:SS"/>

</operator>

<operator activated="true" class="generate_attributes" compatibility="6.4.000" expanded="true" height="76" name="Generate Attributes" width="90" x="581" y="120">

<list key="function_descriptions">

<parameter key="Age" value="date_diff(dob,dod)/31556952000"/>

</list>

</operator>

<connect from_port="in 1" to_op="Set Role" to_port="example set input"/>

<connect from_op="Set Role" from_port="example set output" to_op="Guess Types" to_port="example set input"/>

<connect from_op="Guess Types" from_port="example set output" to_op="Nominal to Date" to_port="example set input"/>

<connect from_op="Nominal to Date" from_port="example set output" to_op="Nominal to Date (2)" to_port="example set input"/>

<connect from_op="Nominal to Date (2)" from_port="example set output" to_op="Generate Attributes" to_port="example set input"/>

<connect from_op="Generate Attributes" from_port="example set output" to_port="out 1"/>

<portSpacing port="source_in 1" spacing="0"/>

<portSpacing port="source_in 2" spacing="0"/>

<portSpacing port="sink_out 1" spacing="0"/>

<portSpacing port="sink_out 2" spacing="0"/>

</process>

</operator>

<operator activated="true" class="filter_examples" compatibility="6.4.000" expanded="true" height="94" name="Filter Adults" width="90" x="452" y="165">

<list key="filters_list">

<parameter key="filters_entry_key" value="icustay_age_group.equals.adult"/>

</list>

</operator>

<operator activated="true" class="guess_types" compatibility="6.5.002" expanded="true" height="76" name="Guess Types (2)" width="90" x="178" y="300">

<parameter key="include_special_attributes" value="true"/>

</operator>

<operator activated="true" class="join" compatibility="6.5.002" expanded="true" height="76" name="Join All" width="90" x="581" y="255">

<parameter key="join_type" value="left"/>

<parameter key="use_id_attribute_as_key" value="false"/>

<list key="key_attributes">

<parameter key="subject_id" value="Subject_ID"/>

</list>

</operator>

<operator activated="true" class="select_attributes" compatibility="6.5.002" expanded="true" height="76" name="Select Attributes" width="90" x="710" y="257">

<parameter key="attribute_filter_type" value="subset"/>

<parameter key="attributes" value="category|dob|dod|expire_flg|hadm_id|height|hospital_admit_dt|hospital_disch_dt|hospital_expire_flg|hospital_first_flg|hospital_last_flg|hospital_los|hospital_seq|hospital_total_num|icustay_age_group|icustay_first_careunit|icustay_first_flg|icustay_intime|icustay_last_careunit|icustay_last_flg|icustay_last_service|icustay_los|icustay_outtime|icustay_seq|icustay_total_num|subject_icustay_seq|subject_icustay_total_num|subject_id|weight_first|weight_loss|weight_max|weight_min"/>

<parameter key="invert_selection" value="true"/>

<parameter key="include_special_attributes" value="true"/>

</operator>

<operator activated="false" class="nominal_to_numerical" compatibility="6.5.002" expanded="true" height="94" name="Nominal to Numerical" width="90" x="45" y="480">

<parameter key="create_view" value="true"/>

<parameter key="value_type" value="polynominal"/>

<list key="comparison_groups"/>

</operator>

<operator activated="false" class="parse_numbers" compatibility="6.5.002" expanded="true" height="76" name="Parse Numbers" width="90" x="179" y="480"/>

<operator activated="true" class="replace_missing_values" compatibility="6.5.002" expanded="true" height="94" name="Replace Missing Values" width="90" x="845" y="255">

<parameter key="include_special_attributes" value="true"/>

<list key="columns"/>

</operator>

<operator activated="true" class="select_attributes" compatibility="6.5.002" expanded="true" height="76" name="Select Attributes (2)" width="90" x="983" y="255">

<parameter key="attribute_filter_type" value="subset"/>

<parameter key="invert_selection" value="true"/>

<parameter key="include_special_attributes" value="true"/>

</operator>

<operator activated="true" class="generate_attributes" compatibility="6.4.000" expanded="true" height="76" name="Add weights" width="90" x="1117" y="255">

<list key="function_descriptions">

<parameter key="weight" value="if(icustay_expire_flg==&quot;Y&quot;,9,1)"/>

</list>

</operator>

<operator activated="true" class="set_role" compatibility="6.5.002" expanded="true" height="76" name="Set Role (2)" width="90" x="1251" y="255">

<parameter key="attribute_name" value="weight"/>

<parameter key="target_role" value="weight"/>

<list key="set_additional_roles"/>

</operator>

<operator activated="false" class="optimize_parameters_grid" compatibility="6.5.002" expanded="true" height="94" name="Optimize Parameters (Grid)" width="90" x="313" y="480">

<list key="parameters">

<parameter key="SVM.kernel_gamma" value="[1e-3;1e3;6;logarithmic]"/>

<parameter key="SVM.C" value="[1e-3;1e3;6;logarithmic]"/>

</list>

<process expanded="true">

<portSpacing port="source_input 1" spacing="0"/>

<portSpacing port="sink_performance" spacing="0"/>

<portSpacing port="sink_result 1" spacing="0"/>

</process>

</operator>

<operator activated="true" class="multiply" compatibility="6.5.002" expanded="true" height="130" name="Multiply (2)" width="90" x="1385" y="120"/>

<operator activated="true" class="filter_examples" compatibility="6.4.000" expanded="true" height="94" name="Filter Examples (2)" width="90" x="1376" y="452">

<list key="filters_list">

<parameter key="filters_entry_key" value="icustay_expire_flg.equals.Y"/>

</list>

</operator>

<operator activated="true" class="discretize_by_user_specification" compatibility="6.5.002" expanded="true" height="94" name="Discretize by User Specification (2)" width="90" x="1499" y="452">

<parameter key="create_view" value="true"/>

<parameter key="attribute_filter_type" value="subset"/>

<parameter key="attribute" value="PLT0"/>

<parameter key="attributes" value="PLT0|PLTmin|PLTmean|PLTmax"/>

<list key="classes">

<parameter key="grade 4 thrombocytopenia" value="25.0"/>

<parameter key="grade 3 thrombocytopenia" value="50.0"/>

<parameter key="grade 2 thrombocytopenia" value="75.0"/>

<parameter key="grade 1 thrombocytopenia" value="150.0"/>

<parameter key="normal platelet count" value="450.0"/>

<parameter key="mild thrombocythemia" value="700.0"/>

<parameter key="moderate thrombocythemia" value="900.0"/>

<parameter key="severe thrombocythemia" value="1000.0"/>

<parameter key="extreme thrombocythemia" value="2000.0"/>

</list>

</operator>

<operator activated="true" class="filter_examples" compatibility="6.4.000" expanded="true" height="94" name="Filter Examples" width="90" x="1376" y="338">

<list key="filters_list">

<parameter key="filters_entry_key" value="icustay_expire_flg.equals.N"/>

</list>

</operator>

<operator activated="true" class="discretize_by_user_specification" compatibility="6.5.002" expanded="true" height="94" name="Discretize by User Specification" width="90" x="1498" y="338">

<parameter key="create_view" value="true"/>

<parameter key="attribute_filter_type" value="subset"/>

<parameter key="attribute" value="PLT0"/>

<parameter key="attributes" value="PLT0|PLTmin|PLTmean|PLTmax"/>

<list key="classes">

<parameter key="grade 4 thrombocytopenia" value="25.0"/>

<parameter key="grade 3 thrombocytopenia" value="50.0"/>

<parameter key="grade 2 thrombocytopenia" value="75.0"/>

<parameter key="grade 1 thrombocytopenia" value="150.0"/>

<parameter key="normal platelet count" value="450.0"/>

<parameter key="mild thrombocythemia" value="700.0"/>

<parameter key="moderate thrombocythemia" value="900.0"/>

<parameter key="severe thrombocythemia" value="1000.0"/>

<parameter key="extreme thrombocythemia" value="2000.0"/>

</list>

</operator>

<operator activated="true" class="subprocess" compatibility="6.5.002" expanded="true" height="130" name="NB (2)" width="90" x="1529" y="177">

<process expanded="true">

<operator activated="true" class="split_data" compatibility="6.5.002" expanded="true" height="94" name="Split Data" width="90" x="45" y="30">

<enumeration key="partitions">

<parameter key="ratio" value="0.5"/>

<parameter key="ratio" value="0.5"/>

</enumeration>

</operator>

<operator activated="true" class="optimize_selection_forward" compatibility="6.5.002" expanded="true" height="94" name="Forward Selection NB" width="90" x="179" y="210">

<process expanded="true">

<operator activated="true" class="x_validation" compatibility="5.1.002" expanded="true" height="112" name="Validation (3)" width="90" x="45" y="30">

<parameter key="sampling_type" value="stratified sampling"/>

<process expanded="true">

<operator activated="false" class="parallel_decision_tree" compatibility="6.5.002" expanded="true" height="76" name="Decision Tree (2)" width="90" x="112" y="300">

<parameter key="criterion" value="gini_index"/>

</operator>

<operator activated="true" class="naive_bayes" compatibility="6.5.002" expanded="true" height="76" name="Naive Bayes (3)" width="90" x="112" y="30"/>

<connect from_port="training" to_op="Naive Bayes (3)" to_port="training set"/>

<connect from_op="Naive Bayes (3)" from_port="model" to_port="model"/>

<portSpacing port="source_training" spacing="0"/>

<portSpacing port="sink_model" spacing="0"/>

<portSpacing port="sink_through 1" spacing="0"/>

</process>

<process expanded="true">

<operator activated="true" class="apply_model" compatibility="6.5.002" expanded="true" height="76" name="Apply Model (4)" width="90" x="45" y="30">

<list key="application_parameters"/>

</operator>

<operator activated="true" class="performance_binominal_classification" compatibility="6.5.002" expanded="true" height="76" name="Performance (4)" width="90" x="179" y="30">

<parameter key="main_criterion" value="AUC"/>

<parameter key="AUC" value="true"/>

</operator>

<connect from_port="model" to_op="Apply Model (4)" to_port="model"/>

<connect from_port="test set" to_op="Apply Model (4)" to_port="unlabelled data"/>

<connect from_op="Apply Model (4)" from_port="labelled data" to_op="Performance (4)" to_port="labelled data"/>

<connect from_op="Performance (4)" from_port="performance" to_port="averagable 1"/>

<portSpacing port="source_model" spacing="0"/>

<portSpacing port="source_test set" spacing="0"/>

<portSpacing port="source_through 1" spacing="0"/>

<portSpacing port="sink_averagable 1" spacing="0"/>

<portSpacing port="sink_averagable 2" spacing="0"/>

</process>

<description align="center" color="transparent" colored="false" width="126">A cross-validation evaluating a decision tree model.</description>

</operator>

<connect from_port="example set" to_op="Validation (3)" to_port="training"/>

<connect from_op="Validation (3)" from_port="averagable 1" to_port="performance"/>

<portSpacing port="source_example set" spacing="0"/>

<portSpacing port="sink_performance" spacing="0"/>

</process>

<description align="center" color="transparent" colored="false" width="126">Standard Forward Selection with a Decision-Tree</description>

</operator>

<operator activated="true" class="select_by_weights" compatibility="6.5.002" expanded="true" height="94" name="Select by Weights" width="90" x="313" y="75"/>

<operator activated="true" class="x_validation" compatibility="5.1.002" expanded="true" height="112" name="NB" width="90" x="451" y="170">

<parameter key="sampling_type" value="stratified sampling"/>

<process expanded="true">

<operator activated="false" class="apply_model" compatibility="6.5.002" expanded="true" height="76" name="Apply Model (2)" width="90" x="313" y="120">

<list key="application_parameters"/>

</operator>

<operator activated="true" class="naive_bayes" compatibility="6.5.002" expanded="true" height="76" name="Naive Bayes (2)" width="90" x="179" y="30"/>

<operator activated="false" class="support_vector_machine" compatibility="6.5.002" expanded="true" height="112" name="SVM" width="90" x="179" y="255">

<parameter key="kernel_type" value="radial"/>

<parameter key="kernel_gamma" value="0.001"/>

<parameter key="C" value="0.001"/>

</operator>

<connect from_port="training" to_op="Naive Bayes (2)" to_port="training set"/>

<connect from_op="Naive Bayes (2)" from_port="model" to_port="model"/>

<portSpacing port="source_training" spacing="0"/>

<portSpacing port="sink_model" spacing="0"/>

<portSpacing port="sink_through 1" spacing="0"/>

</process>

<process expanded="true">

<operator activated="true" class="apply_model" compatibility="6.5.002" expanded="true" height="76" name="Apply Model (5)" width="90" x="45" y="30">

<list key="application_parameters"/>

</operator>

<operator activated="true" class="performance_binominal_classification" compatibility="6.5.002" expanded="true" height="76" name="Performance" width="90" x="313" y="30">

<parameter key="main_criterion" value="AUC"/>

<parameter key="AUC" value="true"/>

</operator>

<connect from_port="model" to_op="Apply Model (5)" to_port="model"/>

<connect from_port="test set" to_op="Apply Model (5)" to_port="unlabelled data"/>

<connect from_op="Apply Model (5)" from_port="labelled data" to_op="Performance" to_port="labelled data"/>

<connect from_op="Performance" from_port="performance" to_port="averagable 1"/>

<portSpacing port="source_model" spacing="0"/>

<portSpacing port="source_test set" spacing="0"/>

<portSpacing port="source_through 1" spacing="0"/>

<portSpacing port="sink_averagable 1" spacing="0"/>

<portSpacing port="sink_averagable 2" spacing="0"/>

</process>

<description align="center" color="transparent" colored="false" width="126">Learning</description>

</operator>

<connect from_port="in 1" to_op="Split Data" to_port="example set"/>

<connect from_op="Split Data" from_port="partition 1" to_op="Select by Weights" to_port="example set input"/>

<connect from_op="Split Data" from_port="partition 2" to_op="Forward Selection NB" to_port="example set"/>

<connect from_op="Forward Selection NB" from_port="attribute weights" to_op="Select by Weights" to_port="weights"/>

<connect from_op="Select by Weights" from_port="example set output" to_op="NB" to_port="training"/>

<connect from_op="Select by Weights" from_port="weights" to_port="out 3"/>

<connect from_op="NB" from_port="model" to_port="out 1"/>

<connect from_op="NB" from_port="averagable 1" to_port="out 2"/>

<portSpacing port="source_in 1" spacing="0"/>

<portSpacing port="source_in 2" spacing="0"/>

<portSpacing port="sink_out 1" spacing="0"/>

<portSpacing port="sink_out 2" spacing="0"/>

<portSpacing port="sink_out 3" spacing="0"/>

<portSpacing port="sink_out 4" spacing="0"/>

<portSpacing port="sink_out 5" spacing="0"/>

</process>

</operator>

<operator activated="true" class="subprocess" compatibility="6.5.002" expanded="true" height="130" name="SVM Process" width="90" x="1528" y="30">

<process expanded="true">

<operator activated="true" class="nominal_to_numerical" compatibility="6.5.002" expanded="true" height="94" name="Nominal to Numerical (2)" width="90" x="45" y="30">

<list key="comparison_groups"/>

</operator>

<operator activated="true" class="split_data" compatibility="6.5.002" expanded="true" height="94" name="Split Data (2)" width="90" x="179" y="30">

<enumeration key="partitions">

<parameter key="ratio" value="0.5"/>

<parameter key="ratio" value="0.5"/>

</enumeration>

</operator>

<operator activated="true" class="optimize_selection_forward" compatibility="6.5.002" expanded="true" height="94" name="Forward Selection SVM" width="90" x="179" y="210">

<process expanded="true">

<operator activated="true" class="x_validation" compatibility="5.1.002" expanded="true" height="112" name="Validation (2)" width="90" x="45" y="30">

<parameter key="sampling_type" value="stratified sampling"/>

<process expanded="true">

<operator activated="false" class="parallel_decision_tree" compatibility="6.5.002" expanded="true" height="76" name="Decision Tree (3)" width="90" x="112" y="300">

<parameter key="criterion" value="gini_index"/>

</operator>

<operator activated="true" class="naive_bayes" compatibility="6.5.002" expanded="true" height="76" name="Naive Bayes (4)" width="90" x="112" y="30"/>

<connect from_port="training" to_op="Naive Bayes (4)" to_port="training set"/>

<connect from_op="Naive Bayes (4)" from_port="model" to_port="model"/>

<portSpacing port="source_training" spacing="0"/>

<portSpacing port="sink_model" spacing="0"/>

<portSpacing port="sink_through 1" spacing="0"/>

</process>

<process expanded="true">

<operator activated="true" class="apply_model" compatibility="6.5.002" expanded="true" height="76" name="Apply Model (3)" width="90" x="45" y="30">

<list key="application_parameters"/>

</operator>

<operator activated="true" class="performance_binominal_classification" compatibility="6.5.002" expanded="true" height="76" name="Performance (2)" width="90" x="179" y="30">

<parameter key="main_criterion" value="AUC"/>

<parameter key="AUC" value="true"/>

</operator>

<connect from_port="model" to_op="Apply Model (3)" to_port="model"/>

<connect from_port="test set" to_op="Apply Model (3)" to_port="unlabelled data"/>

<connect from_op="Apply Model (3)" from_port="labelled data" to_op="Performance (2)" to_port="labelled data"/>

<connect from_op="Performance (2)" from_port="performance" to_port="averagable 1"/>

<portSpacing port="source_model" spacing="0"/>

<portSpacing port="source_test set" spacing="0"/>

<portSpacing port="source_through 1" spacing="0"/>

<portSpacing port="sink_averagable 1" spacing="0"/>

<portSpacing port="sink_averagable 2" spacing="0"/>

</process>

<description align="center" color="transparent" colored="false" width="126">A cross-validation evaluating a decision tree model.</description>

</operator>

<connect from_port="example set" to_op="Validation (2)" to_port="training"/>

<connect from_op="Validation (2)" from_port="averagable 1" to_port="performance"/>

<portSpacing port="source_example set" spacing="0"/>

<portSpacing port="sink_performance" spacing="0"/>

</process>

<description align="center" color="transparent" colored="false" width="126">Standard Forward Selection with a Naive Bayes&lt;br/&gt;</description>

</operator>

<operator activated="true" class="select_by_weights" compatibility="6.5.002" expanded="true" height="94" name="Select by Weights (2)" width="90" x="447" y="210"/>

<operator activated="true" class="optimize_parameters_grid" compatibility="6.5.002" expanded="true" height="94" name="Optimize Parameters (2)" width="90" x="581" y="30">

<list key="parameters">

<parameter key="SVM (3).C" value="[1e-3;1e3;6;logarithmic]"/>

<parameter key="SVM (3).gamma" value="[1e-3;1e3;6;logarithmic]"/>

</list>

<process expanded="true">

<operator activated="true" class="x_validation" compatibility="5.1.002" expanded="true" height="112" name="Validation" width="90" x="45" y="30">

<parameter key="number_of_validations" value="5"/>

<parameter key="sampling_type" value="stratified sampling"/>

<process expanded="true">

<operator activated="true" class="support_vector_machine_libsvm" compatibility="6.5.002" expanded="true" height="76" name="SVM (3)" width="90" x="112" y="30">

<parameter key="gamma" value="100.00000000000006"/>

<parameter key="C" value="1.0"/>

<list key="class_weights"/>

</operator>

<connect from_port="training" to_op="SVM (3)" to_port="training set"/>

<connect from_op="SVM (3)" from_port="model" to_port="model"/>

<portSpacing port="source_training" spacing="0"/>

<portSpacing port="sink_model" spacing="0"/>

<portSpacing port="sink_through 1" spacing="0"/>

</process>

<process expanded="true">

<operator activated="true" class="apply_model" compatibility="6.5.002" expanded="true" height="76" name="Apply Model" width="90" x="45" y="30">

<list key="application_parameters"/>

</operator>

<operator activated="true" class="performance_binominal_classification" compatibility="6.5.002" expanded="true" height="76" name="SVM Per" width="90" x="179" y="30">

<parameter key="main_criterion" value="AUC"/>

<parameter key="AUC" value="true"/>

</operator>

<operator activated="false" class="performance" compatibility="6.5.002" expanded="true" height="76" name="Performance (5)" width="90" x="179" y="210"/>

<connect from_port="model" to_op="Apply Model" to_port="model"/>

<connect from_port="test set" to_op="Apply Model" to_port="unlabelled data"/>

<connect from_op="Apply Model" from_port="labelled data" to_op="SVM Per" to_port="labelled data"/>

<connect from_op="SVM Per" from_port="performance" to_port="averagable 1"/>

<portSpacing port="source_model" spacing="0"/>

<portSpacing port="source_test set" spacing="0"/>

<portSpacing port="source_through 1" spacing="0"/>

<portSpacing port="sink_averagable 1" spacing="0"/>

<portSpacing port="sink_averagable 2" spacing="0"/>

</process>

<description align="center" color="transparent" colored="false" width="126">A cross-validation evaluating SVM</description>

</operator>

<operator activated="true" class="log" compatibility="6.5.002" expanded="true" height="76" name="Log" width="90" x="186" y="66">

<list key="log">

<parameter key="C" value="operator.SVM (3).parameter.C"/>

<parameter key="gamma" value="operator.SVM (3).parameter.gamma"/>

<parameter key="Performance" value="operator.Validation.value.performance"/>

</list>

</operator>

<operator activated="true" class="multiply" compatibility="6.5.002" expanded="true" height="76" name="Multiply" width="90" x="343" y="66"/>

<connect from_port="input 1" to_op="Validation" to_port="training"/>

<connect from_op="Validation" from_port="averagable 1" to_op="Log" to_port="through 1"/>

<connect from_op="Log" from_port="through 1" to_op="Multiply" to_port="input"/>

<connect from_op="Multiply" from_port="output 1" to_port="performance"/>

<portSpacing port="source_input 1" spacing="0"/>

<portSpacing port="source_input 2" spacing="0"/>

<portSpacing port="sink_performance" spacing="0"/>

<portSpacing port="sink_result 1" spacing="0"/>

</process>

<description align="center" color="transparent" colored="false" width="126">Optimize C and Gamma of a radial SVM using optimize by Grid</description>

</operator>

<operator activated="true" class="multiply" compatibility="6.5.002" expanded="true" height="94" name="Multiply (3)" width="90" x="711" y="101"/>

<operator activated="true" class="log_to_data" compatibility="6.5.002" expanded="true" height="94" name="Log to Data (2)" width="90" x="858" y="98">

<parameter key="log_name" value="Log"/>

</operator>

<operator activated="true" class="multiply" compatibility="6.5.002" expanded="true" height="112" name="Multiply (4)" width="90" x="1007" y="84"/>

<operator activated="true" class="aggregate" compatibility="6.5.002" expanded="true" height="76" name="Aggregate (2)" width="90" x="1207" y="132">

<list key="aggregation_attributes">

<parameter key="gamma" value="average"/>

</list>

<parameter key="group_by_attributes" value="gamma"/>

</operator>

<operator activated="true" class="extract_macro" compatibility="6.5.002" expanded="true" height="60" name="Extract Macro (2)" width="90" x="1343" y="128">

<parameter key="macro" value="xbins"/>

<list key="additional_macros"/>

</operator>

<operator activated="true" class="aggregate" compatibility="6.5.002" expanded="true" height="76" name="Aggregate" width="90" x="1207" y="36">

<list key="aggregation_attributes">

<parameter key="C" value="average"/>

</list>

<parameter key="group_by_attributes" value="C"/>

</operator>

<operator activated="true" class="extract_macro" compatibility="6.5.002" expanded="true" height="60" name="Extract Macro" width="90" x="1340" y="30">

<parameter key="macro" value="ybins"/>

<list key="additional_macros"/>

</operator>

<operator activated="true" class="python_scripting:execute_python" compatibility="6.5.000" expanded="true" height="76" name="Execute Python (2)" width="90" x="1517" y="73">

<parameter key="script" value="import pandas as pd&#10;import matplotlib.pyplot as plt&#10;import numpy as np&#10;&#10;def rm_main(data):&#10;&#10;&#10; y = np.log10(data.iloc[:][&quot;C&quot;])&#10; x = np.log10(data.iloc[:][&quot;gamma&quot;])&#10; z = data.iloc[:][&quot;Performance&quot;]&#10; xbins = %{xbins} # From process&#10; ybins = %{ybins} # From process&#10;&#10; plt.title(&quot;Radial SVM Performance&quot;,fontsize=25)&#10; print x,y,z&#10; hist, xbins, ybins = np.histogram2d(x,y,weights=z,bins=[xbins,ybins])&#10;&#10; #choose either none or gaussian for interpolation&#10; plt.imshow(hist.T,interpolation=&quot;gaussian&quot;, origin='lower')&#10; plt.colorbar()&#10; plt.xlabel(&quot;log10(C)&quot;)&#10; plt.ylabel(&quot;log10(gamma)&quot;)&#10; plt.show()"/>

</operator>

<connect from_port="in 1" to_op="Nominal to Numerical (2)" to_port="example set input"/>

<connect from_op="Nominal to Numerical (2)" from_port="example set output" to_op="Split Data (2)" to_port="example set"/>

<connect from_op="Split Data (2)" from_port="partition 1" to_op="Select by Weights (2)" to_port="example set input"/>

<connect from_op="Split Data (2)" from_port="partition 2" to_op="Forward Selection SVM" to_port="example set"/>

<connect from_op="Forward Selection SVM" from_port="attribute weights" to_op="Select by Weights (2)" to_port="weights"/>

<connect from_op="Select by Weights (2)" from_port="example set output" to_op="Optimize Parameters (2)" to_port="input 1"/>

<connect from_op="Select by Weights (2)" from_port="weights" to_port="out 2"/>

<connect from_op="Optimize Parameters (2)" from_port="performance" to_op="Multiply (3)" to_port="input"/>

<connect from_op="Optimize Parameters (2)" from_port="parameter" to_port="out 1"/>

<connect from_op="Multiply (3)" from_port="output 1" to_op="Log to Data (2)" to_port="through 1"/>

<connect from_op="Multiply (3)" from_port="output 2" to_port="out 4"/>

<connect from_op="Log to Data (2)" from_port="exampleSet" to_op="Multiply (4)" to_port="input"/>

<connect from_op="Multiply (4)" from_port="output 1" to_op="Execute Python (2)" to_port="input 1"/>

<connect from_op="Multiply (4)" from_port="output 2" to_op="Aggregate" to_port="example set input"/>

<connect from_op="Multiply (4)" from_port="output 3" to_op="Aggregate (2)" to_port="example set input"/>

<connect from_op="Aggregate (2)" from_port="example set output" to_op="Extract Macro (2)" to_port="example set"/>

<connect from_op="Aggregate" from_port="example set output" to_op="Extract Macro" to_port="example set"/>

<connect from_op="Execute Python (2)" from_port="output 1" to_port="out 3"/>

<portSpacing port="source_in 1" spacing="0"/>

<portSpacing port="source_in 2" spacing="0"/>

<portSpacing port="sink_out 1" spacing="0"/>

<portSpacing port="sink_out 2" spacing="144"/>

<portSpacing port="sink_out 3" spacing="0"/>

<portSpacing port="sink_out 4" spacing="0"/>

<portSpacing port="sink_out 5" spacing="0"/>

</process>

</operator>

<connect from_op="Read ICUdetail" from_port="output" to_op="Join" to_port="left"/>

<connect from_op="Platelet count" from_port="output" to_op="Guess Types (2)" to_port="example set input"/>

<connect from_op="Comorbidity CSV " from_port="output" to_op="Join" to_port="right"/>

<connect from_op="Join" from_port="join" to_op="Age (Dod-Dob)" to_port="in 1"/>

<connect from_op="Age (Dod-Dob)" from_port="out 1" to_op="Filter Adults" to_port="example set input"/>

<connect from_op="Filter Adults" from_port="example set output" to_op="Join All" to_port="left"/>

<connect from_op="Guess Types (2)" from_port="example set output" to_op="Join All" to_port="right"/>

<connect from_op="Join All" from_port="join" to_op="Select Attributes" to_port="example set input"/>

<connect from_op="Select Attributes" from_port="example set output" to_op="Replace Missing Values" to_port="example set input"/>

<connect from_op="Replace Missing Values" from_port="example set output" to_op="Select Attributes (2)" to_port="example set input"/>

<connect from_op="Select Attributes (2)" from_port="example set output" to_op="Add weights" to_port="example set input"/>

<connect from_op="Add weights" from_port="example set output" to_op="Set Role (2)" to_port="example set input"/>

<connect from_op="Set Role (2)" from_port="example set output" to_op="Multiply (2)" to_port="input"/>

<connect from_op="Multiply (2)" from_port="output 1" to_op="SVM Process" to_port="in 1"/>

<connect from_op="Multiply (2)" from_port="output 2" to_op="NB (2)" to_port="in 1"/>

<connect from_op="Multiply (2)" from_port="output 3" to_op="Filter Examples" to_port="example set input"/>

<connect from_op="Multiply (2)" from_port="output 4" to_op="Filter Examples (2)" to_port="example set input"/>

<connect from_op="Filter Examples (2)" from_port="example set output" to_op="Discretize by User Specification (2)" to_port="example set input"/>

<connect from_op="Discretize by User Specification (2)" from_port="example set output" to_port="result 9"/>

<connect from_op="Filter Examples" from_port="example set output" to_op="Discretize by User Specification" to_port="example set input"/>

<connect from_op="Discretize by User Specification" from_port="example set output" to_port="result 6"/>

<connect from_op="NB (2)" from_port="out 2" to_port="result 4"/>

<connect from_op="NB (2)" from_port="out 3" to_port="result 5"/>

<connect from_op="NB (2)" from_port="out 4" to_port="result 8"/>

<connect from_op="SVM Process" from_port="out 1" to_port="result 2"/>

<connect from_op="SVM Process" from_port="out 2" to_port="result 3"/>

<connect from_op="SVM Process" from_port="out 3" to_port="result 1"/>

<connect from_op="SVM Process" from_port="out 4" to_port="result 7"/>

<portSpacing port="source_input 1" spacing="0"/>

<portSpacing port="sink_result 1" spacing="0"/>

<portSpacing port="sink_result 2" spacing="0"/>

<portSpacing port="sink_result 3" spacing="0"/>

<portSpacing port="sink_result 4" spacing="0"/>

<portSpacing port="sink_result 5" spacing="0"/>

<portSpacing port="sink_result 6" spacing="0"/>

<portSpacing port="sink_result 7" spacing="0"/>

<portSpacing port="sink_result 8" spacing="0"/>

<portSpacing port="sink_result 9" spacing="0"/>

<portSpacing port="sink_result 10" spacing="0"/>

</process>

</operator>

</process>
