# Supplementary material for: Scalable Predictive Analysis in Critically Ill Patients Using a Visual Open Data Analysis Platform
Source: PLoS One. 2016 Jan 5;11(1):e0145791. doi: 10.1371/journal.pone.0145791 (PMC4701479; doi:10.1371/journal.pone.0145791)
Supplement: S2 File — Copy and paste this code into the RapidMiner XML view (remove the previous xml code), click the green check symbol and switch back to the normal Process (diagram) view. (DOCX) [file pone.0145791.s002.docx]

**S2 File:**

Extensible Markup Language (XML) file of the process (modeling and feature selection) as presented in this paper. Copy and paste this code into the RapidMiner XML view (remove the previous xml code), click the green check symbol and switch back to the normal Process (diagram) view.

<?xml version="1.0" encoding="UTF-8" standalone="no"?>

<process version="6.5.002">

<context>

<input/>

<output/>

<macros>

<macro>

<key>basepath</key>

<value>C:\Users\Martin\.RapidMiner\repositories\MIMIC II\</value>

</macro>

</macros>

</context>

<operator activated="true" class="process" compatibility="6.5.002" expanded="true" name="Process">

<parameter key="resultfile" value="/Users/svenvanpoucke/Documents/BigData2015/ArtikelMaud/MIMICplateletsMartin.res"/>

<process expanded="true">

<operator activated="true" class="read_csv" compatibility="6.5.002" expanded="true" height="60" name="Read ICUdetail" width="90" x="45" y="120">

<parameter key="csv_file" value="/Users/svenvanpoucke/Documents/BigData2015/ArtikelMaud/icudetail"/>

<parameter key="first_row_as_names" value="false"/>

<list key="annotations">

<parameter key="0" value="Name"/>

</list>

<parameter key="encoding" value="US-ASCII"/>

<list key="data_set_meta_data_information">

<parameter key="0" value="icustay_id.true.integer.attribute"/>

<parameter key="1" value="subject_id.true.integer.id"/>

<parameter key="2" value="gender.true.polynominal.attribute"/>

<parameter key="3" value="dob.true.polynominal.attribute"/>

<parameter key="4" value="dod.true.polynominal.attribute"/>

<parameter key="5" value="expire_flg.true.polynominal.attribute"/>

<parameter key="6" value="subject_icustay_total_num.true.integer.attribute"/>

<parameter key="7" value="subject_icustay_seq.true.integer.attribute"/>

<parameter key="8" value="hadm_id.true.integer.attribute"/>

<parameter key="9" value="hospital_total_num.true.integer.attribute"/>

<parameter key="10" value="hospital_seq.true.integer.attribute"/>

<parameter key="11" value="hospital_first_flg.true.polynominal.attribute"/>

<parameter key="12" value="hospital_last_flg.true.polynominal.attribute"/>

<parameter key="13" value="hospital_admit_dt.true.polynominal.attribute"/>

<parameter key="14" value="hospital_disch_dt.true.polynominal.attribute"/>

<parameter key="15" value="hospital_los.true.integer.attribute"/>

<parameter key="16" value="hospital_expire_flg.true.polynominal.attribute"/>

<parameter key="17" value="icustay_total_num.true.integer.attribute"/>

<parameter key="18" value="icustay_seq.true.integer.attribute"/>

<parameter key="19" value="icustay_first_flg.true.polynominal.attribute"/>

<parameter key="20" value="icustay_last_flg.true.polynominal.attribute"/>

<parameter key="21" value="icustay_intime.true.polynominal.attribute"/>

<parameter key="22" value="icustay_outtime.true.polynominal.attribute"/>

<parameter key="23" value="icustay_admit_age.true.real.attribute"/>

<parameter key="24" value="icustay_age_group.true.polynominal.attribute"/>

<parameter key="25" value="icustay_los.true.integer.attribute"/>

<parameter key="26" value="icustay_expire_flg.true.polynominal.label"/>

<parameter key="27" value="icustay_first_careunit.true.polynominal.attribute"/>

<parameter key="28" value="icustay_last_careunit.true.polynominal.attribute"/>

<parameter key="29" value="icustay_first_service.true.polynominal.attribute"/>

<parameter key="30" value="icustay_last_service.true.polynominal.attribute"/>

<parameter key="31" value="height.true.real.attribute"/>

<parameter key="32" value="weight_first.true.real.attribute"/>

<parameter key="33" value="weight_min.true.real.attribute"/>

<parameter key="34" value="weight_max.true.real.attribute"/>

<parameter key="35" value="sapsi_first.true.integer.attribute"/>

<parameter key="36" value="sapsi_min.true.integer.attribute"/>

<parameter key="37" value="sapsi_max.true.integer.attribute"/>

<parameter key="38" value="sofa_first.true.integer.attribute"/>

<parameter key="39" value="sofa_min.true.integer.attribute"/>

<parameter key="40" value="sofa_max.true.integer.attribute"/>

</list>

</operator>

<operator activated="true" class="read_csv" compatibility="6.5.002" expanded="true" height="60" name="Platelet count" width="90" x="45" y="300">

<parameter key="csv_file" value="/Users/svenvanpoucke/Documents/BigData2015/ArtikelMaud/Plateletmeanmaxmin0.csv"/>

<parameter key="date_format" value="YYYY-MM-DD hh:mm"/>

<parameter key="first_row_as_names" value="false"/>

<list key="annotations">

<parameter key="0" value="Name"/>

</list>

<parameter key="encoding" value="US-ASCII"/>

<list key="data_set_meta_data_information">

<parameter key="0" value="Subject_ID.true.integer.attribute"/>

<parameter key="1" value="Pcmean.true.integer.attribute"/>

<parameter key="2" value="Pcmin.true.integer.attribute"/>

<parameter key="3" value="Pcmax.true.integer.attribute"/>

<parameter key="4" value="PcT0.true.integer.attribute"/>

</list>

</operator>

<operator activated="true" class="read_csv" compatibility="6.5.002" expanded="true" height="60" name="Comorbidity CSV " width="90" x="45" y="210">

<parameter key="csv_file" value="/Users/svenvanpoucke/Documents/BigData2015/ArtikelMaud/comorbidity"/>

<parameter key="first_row_as_names" value="false"/>

<list key="annotations">

<parameter key="0" value="Name"/>

</list>

<parameter key="encoding" value="US-ASCII"/>

<list key="data_set_meta_data_information">

<parameter key="0" value="subject_id.true.integer.id"/>

<parameter key="1" value="hadm_id.true.integer.attribute"/>

<parameter key="2" value="category.true.polynominal.attribute"/>

<parameter key="3" value="congestive_heart_failure.true.binominal.attribute"/>

<parameter key="4" value="cardiac_arrhythmias.true.binominal.attribute"/>

<parameter key="5" value="valvular_disease.true.binominal.attribute"/>

<parameter key="6" value="pulmonary_circulation.true.binominal.attribute"/>

<parameter key="7" value="peripheral_vascular.true.binominal.attribute"/>

<parameter key="8" value="hypertension.true.binominal.attribute"/>

<parameter key="9" value="paralysis.true.binominal.attribute"/>

<parameter key="10" value="other_neurological.true.binominal.attribute"/>

<parameter key="11" value="chronic_pulmonary.true.binominal.attribute"/>

<parameter key="12" value="diabetes_uncomplicated.true.binominal.attribute"/>

<parameter key="13" value="diabetes_complicated.true.binominal.attribute"/>

<parameter key="14" value="hypothyroidism.true.binominal.attribute"/>

<parameter key="15" value="renal_failure.true.binominal.attribute"/>

<parameter key="16" value="liver_disease.true.binominal.attribute"/>

<parameter key="17" value="peptic_ulcer.true.binominal.attribute"/>

<parameter key="18" value="aids.true.binominal.attribute"/>

<parameter key="19" value="lymphoma.true.binominal.attribute"/>

<parameter key="20" value="metastatic_cancer.true.binominal.attribute"/>

<parameter key="21" value="solid_tumor.true.binominal.attribute"/>

<parameter key="22" value="rheumatoid_arthritis.true.binominal.attribute"/>

<parameter key="23" value="coagulopathy.true.binominal.attribute"/>

<parameter key="24" value="obesity.true.binominal.attribute"/>

<parameter key="25" value="weight_loss.true.binominal.attribute"/>

<parameter key="26" value="fluid_electrolyte.true.binominal.attribute"/>

<parameter key="27" value="blood_loss_anemia.true.binominal.attribute"/>

<parameter key="28" value="deficiency_anemias.true.binominal.attribute"/>

<parameter key="29" value="alcohol_abuse.true.binominal.attribute"/>

<parameter key="30" value="drug_abuse.true.binominal.attribute"/>

<parameter key="31" value="psychoses.true.binominal.attribute"/>

<parameter key="32" value="depression.true.binominal.attribute"/>

</list>

</operator>

<operator activated="true" class="subprocess" compatibility="6.5.002" expanded="true" height="112" name="Data preparation" width="90" x="246" y="165">

<parameter key="parallelize_nested_process" value="true"/>

<process expanded="true">

<operator activated="true" class="join" compatibility="6.5.002" expanded="true" height="76" name="Join" width="90" x="45" y="300">

<list key="key_attributes"/>

</operator>

<operator activated="true" class="subprocess" compatibility="6.5.002" expanded="true" height="76" name="Age (Dod-Dob)" width="90" x="178" y="300">

<process expanded="true">

<operator activated="true" class="set_role" compatibility="6.5.002" expanded="true" height="76" name="Set Role" width="90" x="45" y="120">

<parameter key="attribute_name" value="icustay_id"/>

<parameter key="target_role" value="id"/>

<list key="set_additional_roles">

<parameter key="icustay_expire_flg" value="label"/>

</list>

</operator>

<operator activated="true" class="guess_types" compatibility="6.5.002" expanded="true" height="76" name="Guess Types" width="90" x="179" y="120">

<parameter key="attribute_filter_type" value="subset"/>

<parameter key="attributes" value="dob|dod|hospital_disch_dt|hospital_admit_dt|icustay_intime|icustay_outtime"/>

</operator>

<operator activated="true" class="nominal_to_date" compatibility="6.5.002" expanded="true" height="76" name="Nominal to Date" width="90" x="313" y="120">

<parameter key="attribute_name" value="dob"/>

<parameter key="date_format" value="YYYY-MM-DD HH:MM:SS"/>

</operator>

<operator activated="true" class="nominal_to_date" compatibility="6.5.002" expanded="true" height="76" name="Nominal to Date (2)" width="90" x="447" y="120">

<parameter key="attribute_name" value="dod"/>

<parameter key="date_format" value="YYYY-MM-DD HH:MM:SS"/>

</operator>

<operator activated="true" class="generate_attributes" compatibility="6.4.000" expanded="true" height="76" name="Generate Attributes" width="90" x="581" y="120">

<list key="function_descriptions">

<parameter key="Age" value="date_diff(dob,dod)/31556952000"/>

</list>

</operator>

<connect from_port="in 1" to_op="Set Role" to_port="example set input"/>

<connect from_op="Set Role" from_port="example set output" to_op="Guess Types" to_port="example set input"/>

<connect from_op="Guess Types" from_port="example set output" to_op="Nominal to Date" to_port="example set input"/>

<connect from_op="Nominal to Date" from_port="example set output" to_op="Nominal to Date (2)" to_port="example set input"/>

<connect from_op="Nominal to Date (2)" from_port="example set output" to_op="Generate Attributes" to_port="example set input"/>

<connect from_op="Generate Attributes" from_port="example set output" to_port="out 1"/>

<portSpacing port="source_in 1" spacing="0"/>

<portSpacing port="source_in 2" spacing="0"/>

<portSpacing port="sink_out 1" spacing="0"/>

<portSpacing port="sink_out 2" spacing="0"/>

</process>

</operator>

<operator activated="true" class="filter_examples" compatibility="6.4.000" expanded="true" height="94" name="Filter Adults" width="90" x="318" y="300">

<list key="filters_list">

<parameter key="filters_entry_key" value="icustay_age_group.equals.adult"/>

</list>

</operator>

<operator activated="true" class="guess_types" compatibility="6.5.002" expanded="true" height="76" name="Guess Types (2)" width="90" x="44" y="435">

<parameter key="include_special_attributes" value="true"/>

</operator>

<operator activated="true" class="join" compatibility="6.5.002" expanded="true" height="76" name="Join All" width="90" x="447" y="300">

<parameter key="join_type" value="left"/>

<parameter key="use_id_attribute_as_key" value="false"/>

<list key="key_attributes">

<parameter key="subject_id" value="Subject_ID"/>

</list>

</operator>

<operator activated="true" class="select_attributes" compatibility="6.5.002" expanded="true" height="76" name="Select Attributes" width="90" x="581" y="300">

<parameter key="attribute_filter_type" value="subset"/>

<parameter key="attributes" value="category|dob|dod|expire_flg|hadm_id|height|hospital_admit_dt|hospital_disch_dt|hospital_expire_flg|hospital_first_flg|hospital_last_flg|hospital_los|hospital_seq|hospital_total_num|icustay_age_group|icustay_first_careunit|icustay_first_flg|icustay_intime|icustay_last_careunit|icustay_last_flg|icustay_last_service|icustay_los|icustay_outtime|icustay_seq|icustay_total_num|subject_icustay_seq|subject_icustay_total_num|subject_id|Age|icustay_admit_age|icustay_id"/>

<parameter key="invert_selection" value="true"/>

<parameter key="include_special_attributes" value="true"/>

</operator>

<operator activated="true" class="replace_missing_values" compatibility="6.5.002" expanded="true" height="94" name="Replace Missing Values" width="90" x="715" y="300">

<parameter key="include_special_attributes" value="true"/>

<list key="columns"/>

</operator>

<operator activated="true" class="nominal_to_numerical" compatibility="6.5.002" expanded="true" height="94" name="Nominal to Numerical (3)" width="90" x="830" y="301">

<parameter key="attribute_filter_type" value="value_type"/>

<parameter key="value_type" value="polynominal"/>

<list key="comparison_groups"/>

</operator>

<operator activated="true" class="nominal_to_numerical" compatibility="6.5.002" expanded="true" height="94" name="Nominal to Numerical (4)" width="90" x="953" y="299">

<parameter key="attribute_filter_type" value="value_type"/>

<parameter key="value_type" value="binominal"/>

<parameter key="coding_type" value="unique integers"/>

<list key="comparison_groups"/>

</operator>

<operator activated="true" class="nominal_to_binominal" compatibility="6.5.002" expanded="true" height="94" name="Nominal to Binominal" width="90" x="1088" y="297">

<parameter key="attribute_filter_type" value="single"/>

<parameter key="attribute" value="icustay_expire_flg"/>

<parameter key="include_special_attributes" value="true"/>

</operator>

<operator activated="true" class="remap_binominals" compatibility="6.5.002" expanded="true" height="76" name="Remap Binominals" width="90" x="1230" y="299">

<parameter key="attribute_filter_type" value="single"/>

<parameter key="attribute" value="icustay_expire_flg"/>

<parameter key="include_special_attributes" value="true"/>

<parameter key="negative_value" value="N"/>

<parameter key="positive_value" value="Y"/>

</operator>

<operator activated="true" class="split_data" compatibility="6.5.002" expanded="true" height="94" name="Split Data (3)" width="90" x="1367" y="298">

<enumeration key="partitions">

<parameter key="ratio" value="0.7"/>

<parameter key="ratio" value="0.3"/>

</enumeration>

<parameter key="sampling_type" value="stratified sampling"/>

<parameter key="use_local_random_seed" value="true"/>

</operator>

<connect from_port="in 1" to_op="Join" to_port="left"/>

<connect from_port="in 2" to_op="Join" to_port="right"/>

<connect from_port="in 3" to_op="Guess Types (2)" to_port="example set input"/>

<connect from_op="Join" from_port="join" to_op="Age (Dod-Dob)" to_port="in 1"/>

<connect from_op="Age (Dod-Dob)" from_port="out 1" to_op="Filter Adults" to_port="example set input"/>

<connect from_op="Filter Adults" from_port="example set output" to_op="Join All" to_port="left"/>

<connect from_op="Guess Types (2)" from_port="example set output" to_op="Join All" to_port="right"/>

<connect from_op="Join All" from_port="join" to_op="Select Attributes" to_port="example set input"/>

<connect from_op="Select Attributes" from_port="example set output" to_op="Replace Missing Values" to_port="example set input"/>

<connect from_op="Replace Missing Values" from_port="example set output" to_op="Nominal to Numerical (3)" to_port="example set input"/>

<connect from_op="Nominal to Numerical (3)" from_port="example set output" to_op="Nominal to Numerical (4)" to_port="example set input"/>

<connect from_op="Nominal to Numerical (4)" from_port="example set output" to_op="Nominal to Binominal" to_port="example set input"/>

<connect from_op="Nominal to Binominal" from_port="example set output" to_op="Remap Binominals" to_port="example set input"/>

<connect from_op="Remap Binominals" from_port="example set output" to_op="Split Data (3)" to_port="example set"/>

<connect from_op="Split Data (3)" from_port="partition 1" to_port="out 1"/>

<connect from_op="Split Data (3)" from_port="partition 2" to_port="out 2"/>

<portSpacing port="source_in 1" spacing="0"/>

<portSpacing port="source_in 2" spacing="0"/>

<portSpacing port="source_in 3" spacing="0"/>

<portSpacing port="source_in 4" spacing="0"/>

<portSpacing port="sink_out 1" spacing="0"/>

<portSpacing port="sink_out 2" spacing="0"/>

<portSpacing port="sink_out 3" spacing="0"/>

</process>

</operator>

<operator activated="true" class="loop" compatibility="6.5.002" expanded="true" height="94" name="Loop (2)" width="90" x="447" y="165">

<parameter key="set_iteration_macro" value="true"/>

<parameter key="macro_name" value="iterationWeights"/>

<parameter key="iterations" value="6"/>

<parameter key="parallelize_iteration" value="true"/>

<process expanded="true">

<operator activated="true" class="loop" compatibility="6.5.002" expanded="true" height="94" name="Loop" width="90" x="313" y="30">

<parameter key="set_iteration_macro" value="true"/>

<parameter key="macro_start_value" value="16"/>

<parameter key="parallelize_iteration" value="true"/>

<process expanded="true">

<operator activated="false" class="loop_parameters" compatibility="6.5.002" expanded="true" height="112" name="Loop Parameters" width="90" x="313" y="390">

<list key="parameters">

<parameter key="Select by Weights (3).k" value="[5;75;14;linear]"/>

</list>

<parameter key="parallelize_subprocess" value="true"/>

<process expanded="true">

<operator activated="true" class="select_by_weights" compatibility="6.5.002" expanded="true" height="94" name="Select by Weights (3)" width="90" x="112" y="165">

<parameter key="weight_relation" value="top k"/>

<parameter key="k" value="55"/>

</operator>

<operator activated="true" class="store" compatibility="6.5.002" expanded="true" height="60" name="Store (2)" width="90" x="246" y="345">

<parameter key="repository_entry" value="FilterWeights/weights%{iterationWeights}"/>

</operator>

<operator activated="true" class="select_subprocess" compatibility="6.5.002" expanded="true" height="76" name="Algorithms" width="90" x="447" y="165">

<parameter key="select_which" value="%{iteration}"/>

<parameter key="parallelize_selection_1" value="true"/>

<parameter key="parallelize_selection_2" value="true"/>

<process expanded="true">

<operator activated="true" class="weka:W-DecisionStump" compatibility="5.3.001" expanded="true" height="76" name="W-DecisionStump" width="90" x="45" y="75"/>

<connect from_port="input 1" to_op="W-DecisionStump" to_port="training set"/>

<connect from_op="W-DecisionStump" from_port="model" to_port="output 1"/>

<portSpacing port="source_input 1" spacing="0"/>

<portSpacing port="source_input 2" spacing="0"/>

<portSpacing port="sink_output 1" spacing="0"/>

<portSpacing port="sink_output 2" spacing="0"/>

</process>

<process expanded="true">

<operator activated="true" class="decision_stump" compatibility="6.5.002" expanded="true" height="76" name="Decision Stump" width="90" x="45" y="30"/>

<connect from_port="input 1" to_op="Decision Stump" to_port="training set"/>

<connect from_op="Decision Stump" from_port="model" to_port="output 1"/>

<portSpacing port="source_input 1" spacing="0"/>

<portSpacing port="source_input 2" spacing="0"/>

<portSpacing port="sink_output 1" spacing="0"/>

<portSpacing port="sink_output 2" spacing="0"/>

</process>

<process expanded="true">

<operator activated="true" class="weka:W-J48" compatibility="5.3.001" expanded="true" height="76" name="W-J48" width="90" x="112" y="30"/>

<connect from_port="input 1" to_op="W-J48" to_port="training set"/>

<connect from_op="W-J48" from_port="model" to_port="output 1"/>

<portSpacing port="source_input 1" spacing="0"/>

<portSpacing port="source_input 2" spacing="0"/>

<portSpacing port="sink_output 1" spacing="0"/>

<portSpacing port="sink_output 2" spacing="0"/>

</process>

<process expanded="true">

<operator activated="true" class="naive_bayes" compatibility="6.5.002" expanded="true" height="76" name="Naive Bayes (6)" width="90" x="112" y="30"/>

<connect from_port="input 1" to_op="Naive Bayes (6)" to_port="training set"/>

<connect from_op="Naive Bayes (6)" from_port="model" to_port="output 1"/>

<portSpacing port="source_input 1" spacing="0"/>

<portSpacing port="source_input 2" spacing="0"/>

<portSpacing port="sink_output 1" spacing="0"/>

<portSpacing port="sink_output 2" spacing="0"/>

</process>

<process expanded="true">

<operator activated="true" class="weka:W-Logistic" compatibility="5.3.001" expanded="true" height="76" name="W-Logistic" width="90" x="45" y="30"/>

<connect from_port="input 1" to_op="W-Logistic" to_port="training set"/>

<connect from_op="W-Logistic" from_port="model" to_port="output 1"/>

<portSpacing port="source_input 1" spacing="0"/>

<portSpacing port="source_input 2" spacing="0"/>

<portSpacing port="sink_output 1" spacing="0"/>

<portSpacing port="sink_output 2" spacing="0"/>

</process>

<process expanded="true">

<operator activated="true" class="parallel_random_forest" compatibility="6.5.002" expanded="true" height="76" name="RF - Rapid" width="90" x="45" y="30">

<parameter key="number_of_trees" value="30"/>

</operator>

<connect from_port="input 1" to_op="RF - Rapid" to_port="training set"/>

<connect from_op="RF - Rapid" from_port="model" to_port="output 1"/>

<portSpacing port="source_input 1" spacing="0"/>

<portSpacing port="source_input 2" spacing="0"/>

<portSpacing port="sink_output 1" spacing="0"/>

<portSpacing port="sink_output 2" spacing="0"/>

</process>

<process expanded="true">

<operator activated="true" class="weka:W-RandomForest" compatibility="5.3.001" expanded="true" height="76" name="RF - Weka" width="90" x="45" y="30"/>

<connect from_port="input 1" to_op="RF - Weka" to_port="training set"/>

<connect from_op="RF - Weka" from_port="model" to_port="output 1"/>

<portSpacing port="source_input 1" spacing="0"/>

<portSpacing port="source_input 2" spacing="0"/>

<portSpacing port="sink_output 1" spacing="0"/>

<portSpacing port="sink_output 2" spacing="0"/>

</process>

<process expanded="true">

<operator activated="true" class="adaboost" compatibility="6.5.002" expanded="true" height="76" name="AdaBoost (DS)" width="90" x="45" y="30">

<parameter key="parallelize_learning_process" value="true"/>

<process expanded="true">

<operator activated="true" class="decision_stump" compatibility="6.5.002" expanded="true" height="76" name="Decision Stump (2)" width="90" x="112" y="75"/>

<connect from_port="training set" to_op="Decision Stump (2)" to_port="training set"/>

<connect from_op="Decision Stump (2)" from_port="model" to_port="model"/>

<portSpacing port="source_training set" spacing="0"/>

<portSpacing port="sink_model" spacing="0"/>

</process>

</operator>

<connect from_port="input 1" to_op="AdaBoost (DS)" to_port="training set"/>

<connect from_op="AdaBoost (DS)" from_port="model" to_port="output 1"/>

<portSpacing port="source_input 1" spacing="0"/>

<portSpacing port="source_input 2" spacing="0"/>

<portSpacing port="sink_output 1" spacing="0"/>

<portSpacing port="sink_output 2" spacing="0"/>

</process>

<process expanded="true">

<operator activated="true" class="adaboost" compatibility="6.5.002" expanded="true" height="76" name="AdaBoost (J4.8)" width="90" x="45" y="30">

<parameter key="parallelize_learning_process" value="true"/>

<process expanded="true">

<operator activated="true" class="weka:W-J48" compatibility="5.3.001" expanded="true" height="76" name="W-J48 (2)" width="90" x="380" y="75"/>

<connect from_port="training set" to_op="W-J48 (2)" to_port="training set"/>

<connect from_op="W-J48 (2)" from_port="model" to_port="model"/>

<portSpacing port="source_training set" spacing="0"/>

<portSpacing port="sink_model" spacing="0"/>

</process>

</operator>

<connect from_port="input 1" to_op="AdaBoost (J4.8)" to_port="training set"/>

<connect from_op="AdaBoost (J4.8)" from_port="model" to_port="output 1"/>

<portSpacing port="source_input 1" spacing="0"/>

<portSpacing port="source_input 2" spacing="0"/>

<portSpacing port="sink_output 1" spacing="0"/>

<portSpacing port="sink_output 2" spacing="0"/>

</process>

<process expanded="true">

<operator activated="true" class="adaboost" compatibility="6.5.002" expanded="true" height="76" name="AdaBoost (NB)" width="90" x="45" y="30">

<parameter key="parallelize_learning_process" value="true"/>

<process expanded="true">

<operator activated="true" class="naive_bayes" compatibility="6.5.002" expanded="true" height="76" name="Naive Bayes (8)" width="90" x="313" y="75"/>

<connect from_port="training set" to_op="Naive Bayes (8)" to_port="training set"/>

<connect from_op="Naive Bayes (8)" from_port="model" to_port="model"/>

<portSpacing port="source_training set" spacing="0"/>

<portSpacing port="sink_model" spacing="0"/>

</process>

</operator>

<connect from_port="input 1" to_op="AdaBoost (NB)" to_port="training set"/>

<connect from_op="AdaBoost (NB)" from_port="model" to_port="output 1"/>

<portSpacing port="source_input 1" spacing="0"/>

<portSpacing port="source_input 2" spacing="0"/>

<portSpacing port="sink_output 1" spacing="0"/>

<portSpacing port="sink_output 2" spacing="0"/>

</process>

<process expanded="true">

<operator activated="true" class="adaboost" compatibility="6.5.002" expanded="true" height="76" name="AdaBoost (LR)" width="90" x="45" y="30">

<parameter key="parallelize_learning_process" value="true"/>

<process expanded="true">

<operator activated="true" class="weka:W-Logistic" compatibility="5.3.001" expanded="true" height="76" name="W-Logistic (5)" width="90" x="313" y="75"/>

<connect from_port="training set" to_op="W-Logistic (5)" to_port="training set"/>

<connect from_op="W-Logistic (5)" from_port="model" to_port="model"/>

<portSpacing port="source_training set" spacing="0"/>

<portSpacing port="sink_model" spacing="0"/>

</process>

</operator>

<connect from_port="input 1" to_op="AdaBoost (LR)" to_port="training set"/>

<connect from_op="AdaBoost (LR)" from_port="model" to_port="output 1"/>

<portSpacing port="source_input 1" spacing="0"/>

<portSpacing port="source_input 2" spacing="0"/>

<portSpacing port="sink_output 1" spacing="0"/>

<portSpacing port="sink_output 2" spacing="0"/>

</process>

<process expanded="true">

<operator activated="true" class="bagging" compatibility="6.5.002" expanded="true" height="76" name="Bagging (DS)" width="90" x="112" y="30">

<parameter key="sample_ratio" value="0.7"/>

<parameter key="use_local_random_seed" value="true"/>

<parameter key="parallelize_learning_process" value="true"/>

<process expanded="true">

<operator activated="true" class="decision_stump" compatibility="6.5.002" expanded="true" height="76" name="Decision Stump (3)" width="90" x="313" y="75"/>

<connect from_port="training set" to_op="Decision Stump (3)" to_port="training set"/>

<connect from_op="Decision Stump (3)" from_port="model" to_port="model"/>

<portSpacing port="source_training set" spacing="0"/>

<portSpacing port="sink_model" spacing="0"/>

</process>

</operator>

<connect from_port="input 1" to_op="Bagging (DS)" to_port="training set"/>

<connect from_op="Bagging (DS)" from_port="model" to_port="output 1"/>

<portSpacing port="source_input 1" spacing="0"/>

<portSpacing port="source_input 2" spacing="0"/>

<portSpacing port="sink_output 1" spacing="0"/>

<portSpacing port="sink_output 2" spacing="0"/>

</process>

<process expanded="true">

<operator activated="true" class="bagging" compatibility="6.5.002" expanded="true" height="76" name="Bagging (J4.8)" width="90" x="45" y="30">

<parameter key="sample_ratio" value="0.7"/>

<parameter key="use_local_random_seed" value="true"/>

<parameter key="parallelize_learning_process" value="true"/>

<process expanded="true">

<operator activated="true" class="weka:W-J48" compatibility="5.3.001" expanded="true" height="76" name="W-J48 (4)" width="90" x="246" y="75"/>

<connect from_port="training set" to_op="W-J48 (4)" to_port="training set"/>

<connect from_op="W-J48 (4)" from_port="model" to_port="model"/>

<portSpacing port="source_training set" spacing="0"/>

<portSpacing port="sink_model" spacing="0"/>

</process>

</operator>

<connect from_port="input 1" to_op="Bagging (J4.8)" to_port="training set"/>

<connect from_op="Bagging (J4.8)" from_port="model" to_port="output 1"/>

<portSpacing port="source_input 1" spacing="0"/>

<portSpacing port="source_input 2" spacing="0"/>

<portSpacing port="sink_output 1" spacing="0"/>

<portSpacing port="sink_output 2" spacing="0"/>

</process>

<process expanded="true">

<operator activated="true" class="bagging" compatibility="6.5.002" expanded="true" height="76" name="Bagging (NB)" width="90" x="85" y="30">

<parameter key="sample_ratio" value="0.7"/>

<process expanded="true">

<operator activated="true" class="naive_bayes" compatibility="6.5.002" expanded="true" height="76" name="Naive Bayes (9)" width="90" x="380" y="75"/>

<connect from_port="training set" to_op="Naive Bayes (9)" to_port="training set"/>

<connect from_op="Naive Bayes (9)" from_port="model" to_port="model"/>

<portSpacing port="source_training set" spacing="0"/>

<portSpacing port="sink_model" spacing="0"/>

</process>

</operator>

<connect from_port="input 1" to_op="Bagging (NB)" to_port="training set"/>

<connect from_op="Bagging (NB)" from_port="model" to_port="output 1"/>

<portSpacing port="source_input 1" spacing="0"/>

<portSpacing port="source_input 2" spacing="0"/>

<portSpacing port="sink_output 1" spacing="0"/>

<portSpacing port="sink_output 2" spacing="0"/>

</process>

<process expanded="true">

<operator activated="true" class="bagging" compatibility="6.5.002" expanded="true" height="76" name="Bagging (LR)" width="90" x="45" y="30">

<parameter key="sample_ratio" value="0.7"/>

<parameter key="use_local_random_seed" value="true"/>

<parameter key="parallelize_learning_process" value="true"/>

<process expanded="true">

<operator activated="true" class="weka:W-Logistic" compatibility="5.3.001" expanded="true" height="76" name="W-Logistic (4)" width="90" x="313" y="75"/>

<connect from_port="training set" to_op="W-Logistic (4)" to_port="training set"/>

<connect from_op="W-Logistic (4)" from_port="model" to_port="model"/>

<portSpacing port="source_training set" spacing="0"/>

<portSpacing port="sink_model" spacing="0"/>

</process>

</operator>

<connect from_port="input 1" to_op="Bagging (LR)" to_port="training set"/>

<connect from_op="Bagging (LR)" from_port="model" to_port="output 1"/>

<portSpacing port="source_input 1" spacing="0"/>

<portSpacing port="source_input 2" spacing="0"/>

<portSpacing port="sink_output 1" spacing="0"/>

<portSpacing port="sink_output 2" spacing="0"/>

</process>

<process expanded="true">

<operator activated="true" class="stacking" compatibility="6.5.002" expanded="true" height="60" name="Stacking (NB J4.8 LR)" width="90" x="45" y="30">

<parameter key="parallelize_base_learner" value="true"/>

<parameter key="parallelize_stacking_model_learner" value="true"/>

<process expanded="true">

<operator activated="true" class="naive_bayes" compatibility="6.5.002" expanded="true" height="76" name="Naive Bayes (7)" width="90" x="112" y="75"/>

<operator activated="true" class="weka:W-Logistic" compatibility="5.3.001" expanded="true" height="76" name="W-Logistic (2)" width="90" x="112" y="210"/>

<operator activated="true" class="weka:W-J48" compatibility="5.3.001" expanded="true" height="76" name="W-J48 (3)" width="90" x="112" y="300"/>

<connect from_port="training set 1" to_op="Naive Bayes (7)" to_port="training set"/>

<connect from_port="training set 2" to_op="W-Logistic (2)" to_port="training set"/>

<connect from_port="training set 3" to_op="W-J48 (3)" to_port="training set"/>

<connect from_op="Naive Bayes (7)" from_port="model" to_port="base model 1"/>

<connect from_op="W-Logistic (2)" from_port="model" to_port="base model 2"/>

<connect from_op="W-J48 (3)" from_port="model" to_port="base model 3"/>

<portSpacing port="source_training set 1" spacing="0"/>

<portSpacing port="source_training set 2" spacing="0"/>

<portSpacing port="source_training set 3" spacing="0"/>

<portSpacing port="source_training set 4" spacing="0"/>

<portSpacing port="sink_base model 1" spacing="0"/>

<portSpacing port="sink_base model 2" spacing="0"/>

<portSpacing port="sink_base model 3" spacing="0"/>

<portSpacing port="sink_base model 4" spacing="0"/>

</process>

<process expanded="true">

<operator activated="true" class="weka:W-J48" compatibility="5.3.001" expanded="true" height="76" name="W-J48 (5)" width="90" x="112" y="75"/>

<connect from_port="stacking examples" to_op="W-J48 (5)" to_port="training set"/>

<connect from_op="W-J48 (5)" from_port="model" to_port="stacking model"/>

<portSpacing port="source_stacking examples" spacing="0"/>

<portSpacing port="sink_stacking model" spacing="0"/>

</process>

</operator>

<connect from_port="input 1" to_op="Stacking (NB J4.8 LR)" to_port="training set"/>

<connect from_op="Stacking (NB J4.8 LR)" from_port="model" to_port="output 1"/>

<portSpacing port="source_input 1" spacing="0"/>

<portSpacing port="source_input 2" spacing="0"/>

<portSpacing port="sink_output 1" spacing="0"/>

<portSpacing port="sink_output 2" spacing="0"/>

</process>

<process expanded="true">

<operator activated="true" class="multiply" compatibility="6.5.002" expanded="true" height="94" name="Multiply (5)" width="90" x="45" y="165"/>

<operator activated="true" class="optimize_parameters_evolutionary" compatibility="6.5.002" expanded="true" height="112" name="Optimize SVM" width="90" x="112" y="30">

<list key="parameters">

<parameter key="SVM (Linear).C" value="[0.000000001;100000]"/>

</list>

<parameter key="max_generations" value="10"/>

<parameter key="use_early_stopping" value="true"/>

<parameter key="population_size" value="10"/>

<parameter key="use_local_random_seed" value="true"/>

<parameter key="parallelize_optimization_process" value="true"/>

<process expanded="true">

<operator activated="true" class="parallel:x_validation_parallel" compatibility="5.3.000" expanded="true" height="112" name="Validation (5)" width="90" x="246" y="120">

<parameter key="number_of_validations" value="5"/>

<parameter key="use_local_random_seed" value="true"/>

<parameter key="parallelize_training" value="true"/>

<parameter key="parallelize_testing" value="true"/>

<process expanded="true">

<operator activated="true" class="support_vector_machine_linear" compatibility="6.5.002" expanded="true" height="76" name="SVM (Linear)" width="90" x="179" y="75">

<parameter key="C" value="6120.417050152911"/>

</operator>

<connect from_port="training" to_op="SVM (Linear)" to_port="training set"/>

<connect from_op="SVM (Linear)" from_port="model" to_port="model"/>

<portSpacing port="source_training" spacing="0"/>

<portSpacing port="sink_model" spacing="0"/>

<portSpacing port="sink_through 1" spacing="0"/>

</process>

<process expanded="true">

<operator activated="true" class="apply_model" compatibility="6.5.002" expanded="true" height="76" name="Apply Model (7)" width="90" x="112" y="120">

<list key="application_parameters"/>

</operator>

<operator activated="true" class="subprocess" compatibility="6.5.002" expanded="true" height="76" name="AUPRC Optim param I" width="90" x="246" y="120">

<process expanded="true">

<operator activated="true" class="rename" compatibility="6.5.002" expanded="true" height="76" name="Rename (2)" width="90" x="45" y="30">

<parameter key="old_name" value="confidence(N)"/>

<parameter key="new_name" value="noconf"/>

<list key="rename_additional_attributes">

<parameter key="confidence(Y)" value="yesconf"/>

</list>

</operator>

<operator activated="true" class="r_scripting:execute_r" compatibility="6.5.000" expanded="true" height="76" name="Execute R (2)" width="90" x="179" y="30">

<parameter key="script" value="# rm_main is a mandatory function, &#10;# the number of arguments has to be the number of input ports (can be none)&#10;rm_main = function(data)&#10;{&#10; library(PRROC)&#10; data$class[data$icustay_expire_flg== &quot;Y&quot;]&lt;-1 &#10; data$class[data$icustay_expire_flg== &quot;N&quot;]&lt;-0&#10; data$class &lt;- as.numeric(data$class)&#10; x=pr.curve(scores.class0 = data$yesconf, weights.class0 = data$class)&#10; #y=roc.curve(scores.class0 = data$yesconf, weights.class0 = data$class)&#10; &#10; #a&lt;-x$auc.integral&#10; data$AUPRC&lt;- x$auc.integral&#10; #data$AUC &lt;- y$auc.integral&#10; metaData$data$AUPRC &lt;&lt;- list(type=&quot;numeric&quot;, role=&quot;performance&quot;)&#10; #metaData$data$AUC &lt;&lt;- list(type=&quot;numeric&quot;, role=&quot;performance&quot;)&#10; return(data)&#10;}&#10;"/>

</operator>

<operator activated="true" class="extract_performance" compatibility="6.5.002" expanded="true" height="76" name="AUPRC (2)" width="90" x="313" y="30">

<parameter key="performance_type" value="data_value"/>

<parameter key="attribute_name" value="AUPRC"/>

<parameter key="example_index" value="1"/>

</operator>

<connect from_port="in 1" to_op="Rename (2)" to_port="example set input"/>

<connect from_op="Rename (2)" from_port="example set output" to_op="Execute R (2)" to_port="input 1"/>

<connect from_op="Execute R (2)" from_port="output 1" to_op="AUPRC (2)" to_port="example set"/>

<connect from_op="AUPRC (2)" from_port="performance" to_port="out 1"/>

<portSpacing port="source_in 1" spacing="0"/>

<portSpacing port="source_in 2" spacing="0"/>

<portSpacing port="sink_out 1" spacing="0"/>

<portSpacing port="sink_out 2" spacing="0"/>

</process>

</operator>

<operator activated="false" class="performance_binominal_classification" compatibility="6.5.002" expanded="true" height="76" name="Performance (6)" width="90" x="246" y="300">

<parameter key="accuracy" value="false"/>

<parameter key="f_measure" value="true"/>

</operator>

<connect from_port="model" to_op="Apply Model (7)" to_port="model"/>

<connect from_port="test set" to_op="Apply Model (7)" to_port="unlabelled data"/>

<connect from_op="Apply Model (7)" from_port="labelled data" to_op="AUPRC Optim param I" to_port="in 1"/>

<connect from_op="AUPRC Optim param I" from_port="out 1" to_port="averagable 1"/>

<portSpacing port="source_model" spacing="0"/>

<portSpacing port="source_test set" spacing="0"/>

<portSpacing port="source_through 1" spacing="0"/>

<portSpacing port="sink_averagable 1" spacing="0"/>

<portSpacing port="sink_averagable 2" spacing="0"/>

</process>

</operator>

<connect from_port="input 1" to_op="Validation (5)" to_port="training"/>

<connect from_op="Validation (5)" from_port="model" to_port="result 1"/>

<connect from_op="Validation (5)" from_port="averagable 1" to_port="performance"/>

<portSpacing port="source_input 1" spacing="0"/>

<portSpacing port="source_input 2" spacing="0"/>

<portSpacing port="sink_performance" spacing="0"/>

<portSpacing port="sink_result 1" spacing="0"/>

<portSpacing port="sink_result 2" spacing="0"/>

</process>

</operator>

<operator activated="true" class="set_parameters" compatibility="6.5.002" expanded="true" height="76" name="Set Parameters" width="90" x="246" y="165">

<list key="name_map">

<parameter key="SVM (Linear)" value=" Optimal SVM Linear"/>

</list>

</operator>

<operator activated="true" class="support_vector_machine_linear" compatibility="6.5.002" expanded="true" height="76" name="Optimal SVM Linear" width="90" x="179" y="300"/>

<connect from_port="input 1" to_op="Multiply (5)" to_port="input"/>

<connect from_op="Multiply (5)" from_port="output 1" to_op="Optimize SVM" to_port="input 1"/>

<connect from_op="Multiply (5)" from_port="output 2" to_op="Optimal SVM Linear" to_port="training set"/>

<connect from_op="Optimize SVM" from_port="parameter" to_op="Set Parameters" to_port="parameter set"/>

<connect from_op="Optimal SVM Linear" from_port="model" to_port="output 1"/>

<portSpacing port="source_input 1" spacing="0"/>

<portSpacing port="source_input 2" spacing="0"/>

<portSpacing port="sink_output 1" spacing="0"/>

<portSpacing port="sink_output 2" spacing="0"/>

</process>

<process expanded="true">

<operator activated="true" class="multiply" compatibility="6.5.002" expanded="true" height="94" name="Multiply (6)" width="90" x="45" y="165"/>

<operator activated="true" class="support_vector_machine_libsvm" compatibility="6.5.002" expanded="true" height="76" name="Optimal SVM rbf" width="90" x="246" y="300">

<parameter key="C" value="68146.13250384372"/>

<parameter key="nu" value="0.12334713683253792"/>

<list key="class_weights"/>

</operator>

<operator activated="true" class="optimize_parameters_evolutionary" compatibility="6.5.002" expanded="true" height="112" name="Optimize Parameters (5)" width="90" x="112" y="30">

<list key="parameters">

<parameter key="SVM (rbf).C" value="[0.000000001;100000]"/>

<parameter key="SVM (rbf).nu" value="[0.001;2]"/>

</list>

<parameter key="max_generations" value="10"/>

<parameter key="use_early_stopping" value="true"/>

<parameter key="population_size" value="10"/>

<parameter key="use_local_random_seed" value="true"/>

<parameter key="parallelize_optimization_process" value="true"/>

<process expanded="true">

<operator activated="true" class="parallel:x_validation_parallel" compatibility="5.3.000" expanded="true" height="112" name="Validation (7)" width="90" x="380" y="120">

<parameter key="number_of_validations" value="5"/>

<parameter key="use_local_random_seed" value="true"/>

<parameter key="parallelize_training" value="true"/>

<parameter key="parallelize_testing" value="true"/>

<process expanded="true">

<operator activated="true" class="support_vector_machine_libsvm" compatibility="6.5.002" expanded="true" height="76" name="SVM (rbf)" width="90" x="179" y="75">

<parameter key="C" value="68146.13250384372"/>

<parameter key="nu" value="0.12334713683253792"/>

<list key="class_weights"/>

</operator>

<connect from_port="training" to_op="SVM (rbf)" to_port="training set"/>

<connect from_op="SVM (rbf)" from_port="model" to_port="model"/>

<portSpacing port="source_training" spacing="0"/>

<portSpacing port="sink_model" spacing="0"/>

<portSpacing port="sink_through 1" spacing="0"/>

</process>

<process expanded="true">

<operator activated="true" class="apply_model" compatibility="6.5.002" expanded="true" height="76" name="Apply Model (9)" width="90" x="112" y="120">

<list key="application_parameters"/>

</operator>

<operator activated="true" class="subprocess" compatibility="6.5.002" expanded="true" height="76" name="AUPRC Param Optim II" width="90" x="246" y="120">

<process expanded="true">

<operator activated="true" class="rename" compatibility="6.5.002" expanded="true" height="76" name="Rename (3)" width="90" x="45" y="30">

<parameter key="old_name" value="confidence(N)"/>

<parameter key="new_name" value="noconf"/>

<list key="rename_additional_attributes">

<parameter key="confidence(Y)" value="yesconf"/>

</list>

</operator>

<operator activated="true" class="r_scripting:execute_r" compatibility="6.5.000" expanded="true" height="76" name="Execute R (4)" width="90" x="179" y="30">

<parameter key="script" value="# rm_main is a mandatory function, &#10;# the number of arguments has to be the number of input ports (can be none)&#10;rm_main = function(data)&#10;{&#10; library(PRROC)&#10; data$class[data$icustay_expire_flg== &quot;Y&quot;]&lt;-1 &#10; data$class[data$icustay_expire_flg== &quot;N&quot;]&lt;-0&#10; data$class &lt;- as.numeric(data$class)&#10; x=pr.curve(scores.class0 = data$yesconf, weights.class0 = data$class)&#10; #y=roc.curve(scores.class0 = data$yesconf, weights.class0 = data$class)&#10; &#10; #a&lt;-x$auc.integral&#10; data$AUPRC&lt;- x$auc.integral&#10; #data$AUC &lt;- y$auc.integral&#10; metaData$data$AUPRC &lt;&lt;- list(type=&quot;numeric&quot;, role=&quot;performance&quot;)&#10; #metaData$data$AUC &lt;&lt;- list(type=&quot;numeric&quot;, role=&quot;performance&quot;)&#10; return(data)&#10;}&#10;"/>

</operator>

<operator activated="true" class="extract_performance" compatibility="6.5.002" expanded="true" height="76" name="AUPRC (3)" width="90" x="313" y="30">

<parameter key="performance_type" value="data_value"/>

<parameter key="attribute_name" value="AUPRC"/>

<parameter key="example_index" value="1"/>

</operator>

<connect from_port="in 1" to_op="Rename (3)" to_port="example set input"/>

<connect from_op="Rename (3)" from_port="example set output" to_op="Execute R (4)" to_port="input 1"/>

<connect from_op="Execute R (4)" from_port="output 1" to_op="AUPRC (3)" to_port="example set"/>

<connect from_op="AUPRC (3)" from_port="performance" to_port="out 1"/>

<portSpacing port="source_in 1" spacing="0"/>

<portSpacing port="source_in 2" spacing="0"/>

<portSpacing port="sink_out 1" spacing="0"/>

<portSpacing port="sink_out 2" spacing="0"/>

</process>

</operator>

<operator activated="false" class="performance_binominal_classification" compatibility="6.5.002" expanded="true" height="76" name="Performance (8)" width="90" x="246" y="345">

<parameter key="accuracy" value="false"/>

<parameter key="f_measure" value="true"/>

</operator>

<connect from_port="model" to_op="Apply Model (9)" to_port="model"/>

<connect from_port="test set" to_op="Apply Model (9)" to_port="unlabelled data"/>

<connect from_op="Apply Model (9)" from_port="labelled data" to_op="AUPRC Param Optim II" to_port="in 1"/>

<connect from_op="AUPRC Param Optim II" from_port="out 1" to_port="averagable 1"/>

<portSpacing port="source_model" spacing="0"/>

<portSpacing port="source_test set" spacing="0"/>

<portSpacing port="source_through 1" spacing="0"/>

<portSpacing port="sink_averagable 1" spacing="0"/>

<portSpacing port="sink_averagable 2" spacing="0"/>

</process>

</operator>

<connect from_port="input 1" to_op="Validation (7)" to_port="training"/>

<connect from_op="Validation (7)" from_port="model" to_port="result 1"/>

<connect from_op="Validation (7)" from_port="averagable 1" to_port="performance"/>

<portSpacing port="source_input 1" spacing="0"/>

<portSpacing port="source_input 2" spacing="0"/>

<portSpacing port="sink_performance" spacing="0"/>

<portSpacing port="sink_result 1" spacing="0"/>

<portSpacing port="sink_result 2" spacing="0"/>

</process>

</operator>

<operator activated="true" class="set_parameters" compatibility="6.5.002" expanded="true" height="76" name="Set Parameters (2)" width="90" x="246" y="120">

<list key="name_map">

<parameter key="SVM (rbf)" value="Optimal SVM rbf"/>

</list>

</operator>

<connect from_port="input 1" to_op="Multiply (6)" to_port="input"/>

<connect from_op="Multiply (6)" from_port="output 1" to_op="Optimize Parameters (5)" to_port="input 1"/>

<connect from_op="Multiply (6)" from_port="output 2" to_op="Optimal SVM rbf" to_port="training set"/>

<connect from_op="Optimal SVM rbf" from_port="model" to_port="output 1"/>

<connect from_op="Optimize Parameters (5)" from_port="parameter" to_op="Set Parameters (2)" to_port="parameter set"/>

<portSpacing port="source_input 1" spacing="0"/>

<portSpacing port="source_input 2" spacing="0"/>

<portSpacing port="sink_output 1" spacing="0"/>

<portSpacing port="sink_output 2" spacing="0"/>

</process>

<process expanded="true">

<operator activated="true" class="weka:W-SMO" compatibility="5.3.001" expanded="true" height="76" name="W-SMO" width="90" x="45" y="120"/>

<connect from_port="input 1" to_op="W-SMO" to_port="training set"/>

<connect from_op="W-SMO" from_port="model" to_port="output 1"/>

<portSpacing port="source_input 1" spacing="0"/>

<portSpacing port="source_input 2" spacing="0"/>

<portSpacing port="sink_output 1" spacing="0"/>

<portSpacing port="sink_output 2" spacing="0"/>

</process>

</operator>

<operator activated="true" class="apply_model" compatibility="6.5.002" expanded="true" height="76" name="Apply Model (6)" width="90" x="648" y="255">

<list key="application_parameters"/>

</operator>

<operator activated="true" class="subprocess" compatibility="6.5.002" expanded="true" height="76" name="Subprocess (2)" width="90" x="782" y="255">

<parameter key="parallelize_nested_process" value="true"/>

<process expanded="true">

<operator activated="true" class="rename" compatibility="6.5.002" expanded="true" height="76" name="Rename" width="90" x="45" y="30">

<parameter key="old_name" value="confidence(N)"/>

<parameter key="new_name" value="noconf"/>

<list key="rename_additional_attributes">

<parameter key="confidence(Y)" value="yesconf"/>

</list>

</operator>

<operator activated="true" class="r_scripting:execute_r" compatibility="6.5.000" expanded="true" height="76" name="Execute R (3)" width="90" x="179" y="30">

<parameter key="script" value="# rm_main is a mandatory function, &#10;# the number of arguments has to be the number of input ports (can be none)&#10;rm_main = function(data)&#10;{&#10; library(PRROC)&#10; data$class[data$icustay_expire_flg== &quot;Y&quot;]&lt;-1 &#10; data$class[data$icustay_expire_flg== &quot;N&quot;]&lt;-0&#10; data$class &lt;- as.numeric(data$class)&#10; x=pr.curve(scores.class0 = data$yesconf, weights.class0 = data$class)&#10; #y=roc.curve(scores.class0 = data$yesconf, weights.class0 = data$class)&#10; &#10; #a&lt;-x$auc.integral&#10; data$AUPRC&lt;- x$auc.integral&#10; #data$AUC &lt;- y$auc.integral&#10; metaData$data$AUPRC &lt;&lt;- list(type=&quot;numeric&quot;, role=&quot;performance&quot;)&#10; #metaData$data$AUC &lt;&lt;- list(type=&quot;numeric&quot;, role=&quot;performance&quot;)&#10; return(data)&#10;}&#10;"/>

</operator>

<operator activated="true" class="extract_performance" compatibility="6.5.002" expanded="true" height="76" name="AUPRC" width="90" x="313" y="30">

<parameter key="performance_type" value="data_value"/>

<parameter key="attribute_name" value="AUPRC"/>

<parameter key="example_index" value="1"/>

</operator>

<connect from_port="in 1" to_op="Rename" to_port="example set input"/>

<connect from_op="Rename" from_port="example set output" to_op="Execute R (3)" to_port="input 1"/>

<connect from_op="Execute R (3)" from_port="output 1" to_op="AUPRC" to_port="example set"/>

<connect from_op="AUPRC" from_port="performance" to_port="out 1"/>

<portSpacing port="source_in 1" spacing="0"/>

<portSpacing port="source_in 2" spacing="0"/>

<portSpacing port="sink_out 1" spacing="0"/>

<portSpacing port="sink_out 2" spacing="0"/>

</process>

</operator>

<operator activated="true" class="log" compatibility="6.5.002" expanded="true" height="76" name="Log (2)" width="90" x="916" y="255">

<parameter key="filename" value="C:\Users\Milan Vukicevic\Dropbox\SCOPES\Papers\Sven\MIMIC II\results\LogLastWeights.log"/>

<list key="log">

<parameter key="AUC" value="operator.Performance (3).value.AUC"/>

<parameter key="F measure" value="operator.Performance (3).value.f_measure"/>

<parameter key="Recall" value="operator.Performance (3).value.recall"/>

<parameter key="Precision" value="operator.Performance (3).value.precision"/>

<parameter key="Sensitivity" value="operator.Performance (3).value.sensitivity"/>

<parameter key="Specificity" value="operator.Performance (3).value.specificity"/>

<parameter key="Algorithm" value="operator.Loop.value.iteration"/>

<parameter key="FS method" value="operator.Loop (2).value.iteration"/>

<parameter key="Parameters" value="operator.Loop Parameters.value.iteration"/>

</list>

<parameter key="persistent" value="true"/>

</operator>

<connect from_port="input 1" to_op="Select by Weights (3)" to_port="example set input"/>

<connect from_port="input 2" to_op="Select by Weights (3)" to_port="weights"/>

<connect from_port="input 3" to_op="Apply Model (6)" to_port="unlabelled data"/>

<connect from_op="Select by Weights (3)" from_port="example set output" to_op="Algorithms" to_port="input 1"/>

<connect from_op="Select by Weights (3)" from_port="weights" to_op="Store (2)" to_port="input"/>

<connect from_op="Algorithms" from_port="output 1" to_op="Apply Model (6)" to_port="model"/>

<connect from_op="Apply Model (6)" from_port="labelled data" to_op="Subprocess (2)" to_port="in 1"/>

<connect from_op="Subprocess (2)" from_port="out 1" to_op="Log (2)" to_port="through 1"/>

<connect from_op="Log (2)" from_port="through 1" to_port="performance"/>

<portSpacing port="source_input 1" spacing="0"/>

<portSpacing port="source_input 2" spacing="0"/>

<portSpacing port="source_input 3" spacing="0"/>

<portSpacing port="source_input 4" spacing="0"/>

<portSpacing port="sink_performance" spacing="0"/>

<portSpacing port="sink_result 1" spacing="0"/>

</process>

</operator>

<operator activated="true" class="select_subprocess" compatibility="6.5.002" expanded="true" height="112" name="Select Subprocess (2)" width="90" x="45" y="120">

<parameter key="select_which" value="%{iterationWeights}"/>

<parameter key="parallelize_selection_1" value="true"/>

<parameter key="parallelize_selection_2" value="true"/>

<process expanded="true">

<operator activated="true" class="weight_by_information_gain_ratio" compatibility="6.5.002" expanded="true" height="76" name="Weight by Information Gain Ratio (2)" width="90" x="45" y="30">

<parameter key="normalize_weights" value="true"/>

<parameter key="sort_direction" value="descending"/>

</operator>

<connect from_port="input 1" to_op="Weight by Information Gain Ratio (2)" to_port="example set"/>

<connect from_port="input 2" to_port="output 3"/>

<connect from_op="Weight by Information Gain Ratio (2)" from_port="weights" to_port="output 2"/>

<connect from_op="Weight by Information Gain Ratio (2)" from_port="example set" to_port="output 1"/>

<portSpacing port="source_input 1" spacing="0"/>

<portSpacing port="source_input 2" spacing="0"/>

<portSpacing port="source_input 3" spacing="0"/>

<portSpacing port="sink_output 1" spacing="0"/>

<portSpacing port="sink_output 2" spacing="0"/>

<portSpacing port="sink_output 3" spacing="0"/>

<portSpacing port="sink_output 4" spacing="0"/>

</process>

<process expanded="true">

<operator activated="true" class="weka:W-ReliefFAttributeEval" compatibility="5.3.001" expanded="true" height="76" name="W-ReliefFAttributeEval (2)" width="90" x="112" y="30">

<parameter key="normalize_weights" value="true"/>

<parameter key="sort_direction" value="descending"/>

</operator>

<connect from_port="input 1" to_op="W-ReliefFAttributeEval (2)" to_port="example set"/>

<connect from_port="input 2" to_port="output 3"/>

<connect from_op="W-ReliefFAttributeEval (2)" from_port="weights" to_port="output 2"/>

<connect from_op="W-ReliefFAttributeEval (2)" from_port="example set" to_port="output 1"/>

<portSpacing port="source_input 1" spacing="0"/>

<portSpacing port="source_input 2" spacing="0"/>

<portSpacing port="source_input 3" spacing="0"/>

<portSpacing port="sink_output 1" spacing="0"/>

<portSpacing port="sink_output 2" spacing="0"/>

<portSpacing port="sink_output 3" spacing="0"/>

<portSpacing port="sink_output 4" spacing="0"/>

</process>

<process expanded="true">

<operator activated="true" class="featselext:mrmr_feature_selection" compatibility="1.1.004" expanded="true" height="76" name="MRMR-FS (2)" width="90" x="112" y="30">

<parameter key="normalize_weights" value="true"/>

<parameter key="sort_direction" value="descending"/>

</operator>

<connect from_port="input 1" to_op="MRMR-FS (2)" to_port="example set"/>

<connect from_port="input 2" to_port="output 3"/>

<connect from_op="MRMR-FS (2)" from_port="weights" to_port="output 2"/>

<connect from_op="MRMR-FS (2)" from_port="example set" to_port="output 1"/>

<portSpacing port="source_input 1" spacing="0"/>

<portSpacing port="source_input 2" spacing="0"/>

<portSpacing port="source_input 3" spacing="0"/>

<portSpacing port="sink_output 1" spacing="0"/>

<portSpacing port="sink_output 2" spacing="0"/>

<portSpacing port="sink_output 3" spacing="0"/>

<portSpacing port="sink_output 4" spacing="0"/>

</process>

<process expanded="true">

<operator activated="true" class="weight_by_correlation" compatibility="6.5.002" expanded="true" height="76" name="Weight by Correlation (2)" width="90" x="45" y="30">

<parameter key="normalize_weights" value="true"/>

<parameter key="sort_direction" value="descending"/>

</operator>

<connect from_port="input 1" to_op="Weight by Correlation (2)" to_port="example set"/>

<connect from_port="input 2" to_port="output 3"/>

<connect from_op="Weight by Correlation (2)" from_port="weights" to_port="output 2"/>

<connect from_op="Weight by Correlation (2)" from_port="example set" to_port="output 1"/>

<portSpacing port="source_input 1" spacing="0"/>

<portSpacing port="source_input 2" spacing="0"/>

<portSpacing port="source_input 3" spacing="0"/>

<portSpacing port="sink_output 1" spacing="0"/>

<portSpacing port="sink_output 2" spacing="0"/>

<portSpacing port="sink_output 3" spacing="0"/>

<portSpacing port="sink_output 4" spacing="0"/>

</process>

<process expanded="true">

<operator activated="true" class="weight_by_gini_index" compatibility="6.5.002" expanded="true" height="76" name="Weight by Gini Index (2)" width="90" x="45" y="30">

<parameter key="normalize_weights" value="true"/>

<parameter key="sort_direction" value="descending"/>

</operator>

<connect from_port="input 1" to_op="Weight by Gini Index (2)" to_port="example set"/>

<connect from_port="input 2" to_port="output 3"/>

<connect from_op="Weight by Gini Index (2)" from_port="weights" to_port="output 2"/>

<connect from_op="Weight by Gini Index (2)" from_port="example set" to_port="output 1"/>

<portSpacing port="source_input 1" spacing="0"/>

<portSpacing port="source_input 2" spacing="0"/>

<portSpacing port="source_input 3" spacing="0"/>

<portSpacing port="sink_output 1" spacing="0"/>

<portSpacing port="sink_output 2" spacing="0"/>

<portSpacing port="sink_output 3" spacing="0"/>

<portSpacing port="sink_output 4" spacing="0"/>

</process>

<process expanded="true">

<operator activated="true" class="featselext:t_test" compatibility="1.1.004" expanded="true" height="76" name="t-Test (2)" width="90" x="45" y="30"/>

<connect from_port="input 1" to_op="t-Test (2)" to_port="example set"/>

<connect from_port="input 2" to_port="output 3"/>

<connect from_op="t-Test (2)" from_port="weights" to_port="output 2"/>

<connect from_op="t-Test (2)" from_port="example set" to_port="output 1"/>

<portSpacing port="source_input 1" spacing="0"/>

<portSpacing port="source_input 2" spacing="0"/>

<portSpacing port="source_input 3" spacing="0"/>

<portSpacing port="sink_output 1" spacing="0"/>

<portSpacing port="sink_output 2" spacing="0"/>

<portSpacing port="sink_output 3" spacing="0"/>

<portSpacing port="sink_output 4" spacing="0"/>

</process>

</operator>

<operator activated="true" class="store" compatibility="6.5.002" expanded="true" height="60" name="Store (3)" width="90" x="179" y="300">

<parameter key="repository_entry" value="FilterResults/Weights/weights%{iterationWeights}"/>

</operator>

<operator activated="true" class="optimize_parameters_grid" compatibility="6.5.002" expanded="true" height="94" name="Optimize Parameters (3)" width="90" x="246" y="120">

<list key="parameters">

<parameter key="OptimizeK.k" value="[5;75;14;linear]"/>

</list>

<parameter key="parallelize_optimization_process" value="true"/>

<process expanded="true">

<operator activated="true" class="x_validation" compatibility="6.5.002" expanded="true" height="112" name="Validation (4)" width="90" x="246" y="75">

<parameter key="number_of_validations" value="5"/>

<parameter key="use_local_random_seed" value="true"/>

<parameter key="parallelize_training" value="true"/>

<parameter key="parallelize_testing" value="true"/>

<process expanded="true">

<operator activated="true" class="retrieve" compatibility="6.5.002" expanded="true" height="60" name="Retrieve" width="90" x="45" y="165">

<parameter key="repository_entry" value="FilterResults/Weights/weights%{iterationWeights}"/>

</operator>

<operator activated="true" class="select_by_weights" compatibility="6.5.002" expanded="true" height="94" name="OptimizeK" width="90" x="179" y="75">

<parameter key="weight_relation" value="top k"/>

<parameter key="k" value="5"/>

</operator>

<operator activated="true" class="select_subprocess" compatibility="6.5.002" expanded="true" height="76" name="Algorithms (2)" width="90" x="313" y="75">

<parameter key="select_which" value="%{iteration}"/>

<parameter key="parallelize_selection_1" value="true"/>

<parameter key="parallelize_selection_2" value="true"/>

<process expanded="true">

<operator activated="true" class="weka:W-DecisionStump" compatibility="5.3.001" expanded="true" height="76" name="W-DecisionStump (2)" width="90" x="45" y="30"/>

<connect from_port="input 1" to_op="W-DecisionStump (2)" to_port="training set"/>

<connect from_op="W-DecisionStump (2)" from_port="model" to_port="output 1"/>

<portSpacing port="source_input 1" spacing="0"/>

<portSpacing port="source_input 2" spacing="0"/>

<portSpacing port="sink_output 1" spacing="0"/>

<portSpacing port="sink_output 2" spacing="0"/>

</process>

<process expanded="true">

<operator activated="true" class="decision_stump" compatibility="6.5.002" expanded="true" height="76" name="Decision Stump (4)" width="90" x="45" y="30"/>

<connect from_port="input 1" to_op="Decision Stump (4)" to_port="training set"/>

<connect from_op="Decision Stump (4)" from_port="model" to_port="output 1"/>

<portSpacing port="source_input 1" spacing="0"/>

<portSpacing port="source_input 2" spacing="0"/>

<portSpacing port="sink_output 1" spacing="0"/>

<portSpacing port="sink_output 2" spacing="0"/>

</process>

<process expanded="true">

<operator activated="true" class="weka:W-J48" compatibility="5.3.001" expanded="true" height="76" name="W-J48 (6)" width="90" x="112" y="30"/>

<connect from_port="input 1" to_op="W-J48 (6)" to_port="training set"/>

<connect from_op="W-J48 (6)" from_port="model" to_port="output 1"/>

<portSpacing port="source_input 1" spacing="0"/>

<portSpacing port="source_input 2" spacing="0"/>

<portSpacing port="sink_output 1" spacing="0"/>

<portSpacing port="sink_output 2" spacing="0"/>

</process>

<process expanded="true">

<operator activated="true" class="naive_bayes" compatibility="6.5.002" expanded="true" height="76" name="Naive Bayes (5)" width="90" x="112" y="30"/>

<connect from_port="input 1" to_op="Naive Bayes (5)" to_port="training set"/>

<connect from_op="Naive Bayes (5)" from_port="model" to_port="output 1"/>

<portSpacing port="source_input 1" spacing="0"/>

<portSpacing port="source_input 2" spacing="0"/>

<portSpacing port="sink_output 1" spacing="0"/>

<portSpacing port="sink_output 2" spacing="0"/>

</process>

<process expanded="true">

<operator activated="true" class="weka:W-Logistic" compatibility="5.3.001" expanded="true" height="76" name="W-Logistic (3)" width="90" x="45" y="30"/>

<connect from_port="input 1" to_op="W-Logistic (3)" to_port="training set"/>

<connect from_op="W-Logistic (3)" from_port="model" to_port="output 1"/>

<portSpacing port="source_input 1" spacing="0"/>

<portSpacing port="source_input 2" spacing="0"/>

<portSpacing port="sink_output 1" spacing="0"/>

<portSpacing port="sink_output 2" spacing="0"/>

</process>

<process expanded="true">

<operator activated="true" class="parallel_random_forest" compatibility="6.5.002" expanded="true" height="76" name="RF - Rapid (2)" width="90" x="45" y="30">

<parameter key="number_of_trees" value="30"/>

</operator>

<connect from_port="input 1" to_op="RF - Rapid (2)" to_port="training set"/>

<connect from_op="RF - Rapid (2)" from_port="model" to_port="output 1"/>

<portSpacing port="source_input 1" spacing="0"/>

<portSpacing port="source_input 2" spacing="0"/>

<portSpacing port="sink_output 1" spacing="0"/>

<portSpacing port="sink_output 2" spacing="0"/>

</process>

<process expanded="true">

<operator activated="true" class="weka:W-RandomForest" compatibility="5.3.001" expanded="true" height="76" name="RF - Weka (2)" width="90" x="45" y="30"/>

<connect from_port="input 1" to_op="RF - Weka (2)" to_port="training set"/>

<connect from_op="RF - Weka (2)" from_port="model" to_port="output 1"/>

<portSpacing port="source_input 1" spacing="0"/>

<portSpacing port="source_input 2" spacing="0"/>

<portSpacing port="sink_output 1" spacing="0"/>

<portSpacing port="sink_output 2" spacing="0"/>

</process>

<process expanded="true">

<operator activated="true" class="adaboost" compatibility="6.5.002" expanded="true" height="76" name="AdaBoost (2)" width="90" x="45" y="30">

<parameter key="parallelize_learning_process" value="true"/>

<process expanded="true">

<operator activated="true" class="decision_stump" compatibility="6.5.002" expanded="true" height="76" name="Decision Stump (5)" width="90" x="112" y="75"/>

<connect from_port="training set" to_op="Decision Stump (5)" to_port="training set"/>

<connect from_op="Decision Stump (5)" from_port="model" to_port="model"/>

<portSpacing port="source_training set" spacing="0"/>

<portSpacing port="sink_model" spacing="0"/>

</process>

</operator>

<connect from_port="input 1" to_op="AdaBoost (2)" to_port="training set"/>

<connect from_op="AdaBoost (2)" from_port="model" to_port="output 1"/>

<portSpacing port="source_input 1" spacing="0"/>

<portSpacing port="source_input 2" spacing="0"/>

<portSpacing port="sink_output 1" spacing="0"/>

<portSpacing port="sink_output 2" spacing="0"/>

</process>

<process expanded="true">

<operator activated="true" class="adaboost" compatibility="6.5.002" expanded="true" height="76" name="AdaBoost (3)" width="90" x="45" y="30">

<parameter key="parallelize_learning_process" value="true"/>

<process expanded="true">

<operator activated="true" class="weka:W-J48" compatibility="5.3.001" expanded="true" height="76" name="W-J48 (7)" width="90" x="380" y="75"/>

<connect from_port="training set" to_op="W-J48 (7)" to_port="training set"/>

<connect from_op="W-J48 (7)" from_port="model" to_port="model"/>

<portSpacing port="source_training set" spacing="0"/>

<portSpacing port="sink_model" spacing="0"/>

</process>

</operator>

<connect from_port="input 1" to_op="AdaBoost (3)" to_port="training set"/>

<connect from_op="AdaBoost (3)" from_port="model" to_port="output 1"/>

<portSpacing port="source_input 1" spacing="0"/>

<portSpacing port="source_input 2" spacing="0"/>

<portSpacing port="sink_output 1" spacing="0"/>

<portSpacing port="sink_output 2" spacing="0"/>

</process>

<process expanded="true">

<operator activated="true" class="adaboost" compatibility="6.5.002" expanded="true" height="76" name="AdaBoost (4)" width="90" x="45" y="30">

<parameter key="parallelize_learning_process" value="true"/>

<process expanded="true">

<operator activated="true" class="naive_bayes" compatibility="6.5.002" expanded="true" height="76" name="Naive Bayes (10)" width="90" x="313" y="75"/>

<connect from_port="training set" to_op="Naive Bayes (10)" to_port="training set"/>

<connect from_op="Naive Bayes (10)" from_port="model" to_port="model"/>

<portSpacing port="source_training set" spacing="0"/>

<portSpacing port="sink_model" spacing="0"/>

</process>

</operator>

<connect from_port="input 1" to_op="AdaBoost (4)" to_port="training set"/>

<connect from_op="AdaBoost (4)" from_port="model" to_port="output 1"/>

<portSpacing port="source_input 1" spacing="0"/>

<portSpacing port="source_input 2" spacing="0"/>

<portSpacing port="sink_output 1" spacing="0"/>

<portSpacing port="sink_output 2" spacing="0"/>

</process>

<process expanded="true">

<operator activated="true" class="adaboost" compatibility="6.5.002" expanded="true" height="76" name="AdaBoost (5)" width="90" x="45" y="30">

<parameter key="parallelize_learning_process" value="true"/>

<process expanded="true">

<operator activated="true" class="weka:W-Logistic" compatibility="5.3.001" expanded="true" height="76" name="W-Logistic (6)" width="90" x="313" y="75"/>

<connect from_port="training set" to_op="W-Logistic (6)" to_port="training set"/>

<connect from_op="W-Logistic (6)" from_port="model" to_port="model"/>

<portSpacing port="source_training set" spacing="0"/>

<portSpacing port="sink_model" spacing="0"/>

</process>

</operator>

<connect from_port="input 1" to_op="AdaBoost (5)" to_port="training set"/>

<connect from_op="AdaBoost (5)" from_port="model" to_port="output 1"/>

<portSpacing port="source_input 1" spacing="0"/>

<portSpacing port="source_input 2" spacing="0"/>

<portSpacing port="sink_output 1" spacing="0"/>

<portSpacing port="sink_output 2" spacing="0"/>

</process>

<process expanded="true">

<operator activated="true" class="bagging" compatibility="6.5.002" expanded="true" height="76" name="Bagging (2)" width="90" x="112" y="30">

<parameter key="sample_ratio" value="0.7"/>

<parameter key="use_local_random_seed" value="true"/>

<parameter key="parallelize_learning_process" value="true"/>

<process expanded="true">

<operator activated="true" class="decision_stump" compatibility="6.5.002" expanded="true" height="76" name="Decision Stump (6)" width="90" x="313" y="75"/>

<connect from_port="training set" to_op="Decision Stump (6)" to_port="training set"/>

<connect from_op="Decision Stump (6)" from_port="model" to_port="model"/>

<portSpacing port="source_training set" spacing="0"/>

<portSpacing port="sink_model" spacing="0"/>

</process>

</operator>

<connect from_port="input 1" to_op="Bagging (2)" to_port="training set"/>

<connect from_op="Bagging (2)" from_port="model" to_port="output 1"/>

<portSpacing port="source_input 1" spacing="0"/>

<portSpacing port="source_input 2" spacing="0"/>

<portSpacing port="sink_output 1" spacing="0"/>

<portSpacing port="sink_output 2" spacing="0"/>

</process>

<process expanded="true">

<operator activated="true" class="bagging" compatibility="6.5.002" expanded="true" height="76" name="Bagging (3)" width="90" x="45" y="30">

<parameter key="sample_ratio" value="0.7"/>

<parameter key="use_local_random_seed" value="true"/>

<parameter key="parallelize_learning_process" value="true"/>

<process expanded="true">

<operator activated="true" class="weka:W-J48" compatibility="5.3.001" expanded="true" height="76" name="W-J48 (8)" width="90" x="246" y="75"/>

<connect from_port="training set" to_op="W-J48 (8)" to_port="training set"/>

<connect from_op="W-J48 (8)" from_port="model" to_port="model"/>

<portSpacing port="source_training set" spacing="0"/>

<portSpacing port="sink_model" spacing="0"/>

</process>

</operator>

<connect from_port="input 1" to_op="Bagging (3)" to_port="training set"/>

<connect from_op="Bagging (3)" from_port="model" to_port="output 1"/>

<portSpacing port="source_input 1" spacing="0"/>

<portSpacing port="source_input 2" spacing="0"/>

<portSpacing port="sink_output 1" spacing="0"/>

<portSpacing port="sink_output 2" spacing="0"/>

</process>

<process expanded="true">

<operator activated="true" class="bagging" compatibility="6.5.002" expanded="true" height="76" name="Bagging (4)" width="90" x="85" y="30">

<parameter key="sample_ratio" value="0.7"/>

<process expanded="true">

<operator activated="true" class="naive_bayes" compatibility="6.5.002" expanded="true" height="76" name="Naive Bayes (11)" width="90" x="380" y="75"/>

<connect from_port="training set" to_op="Naive Bayes (11)" to_port="training set"/>

<connect from_op="Naive Bayes (11)" from_port="model" to_port="model"/>

<portSpacing port="source_training set" spacing="0"/>

<portSpacing port="sink_model" spacing="0"/>

</process>

</operator>

<connect from_port="input 1" to_op="Bagging (4)" to_port="training set"/>

<connect from_op="Bagging (4)" from_port="model" to_port="output 1"/>

<portSpacing port="source_input 1" spacing="0"/>

<portSpacing port="source_input 2" spacing="0"/>

<portSpacing port="sink_output 1" spacing="0"/>

<portSpacing port="sink_output 2" spacing="0"/>

</process>

<process expanded="true">

<operator activated="true" class="bagging" compatibility="6.5.002" expanded="true" height="76" name="Bagging (5)" width="90" x="45" y="30">

<parameter key="sample_ratio" value="0.7"/>

<parameter key="use_local_random_seed" value="true"/>

<parameter key="parallelize_learning_process" value="true"/>

<process expanded="true">

<operator activated="true" class="weka:W-Logistic" compatibility="5.3.001" expanded="true" height="76" name="W-Logistic (7)" width="90" x="313" y="75"/>

<connect from_port="training set" to_op="W-Logistic (7)" to_port="training set"/>

<connect from_op="W-Logistic (7)" from_port="model" to_port="model"/>

<portSpacing port="source_training set" spacing="0"/>

<portSpacing port="sink_model" spacing="0"/>

</process>

</operator>

<connect from_port="input 1" to_op="Bagging (5)" to_port="training set"/>

<connect from_op="Bagging (5)" from_port="model" to_port="output 1"/>

<portSpacing port="source_input 1" spacing="0"/>

<portSpacing port="source_input 2" spacing="0"/>

<portSpacing port="sink_output 1" spacing="0"/>

<portSpacing port="sink_output 2" spacing="0"/>

</process>

<process expanded="true">

<operator activated="true" class="stacking" compatibility="6.5.002" expanded="true" height="60" name="Stacking (2)" width="90" x="45" y="30">

<parameter key="parallelize_base_learner" value="true"/>

<parameter key="parallelize_stacking_model_learner" value="true"/>

<process expanded="true">

<operator activated="true" class="naive_bayes" compatibility="6.5.002" expanded="true" height="76" name="Naive Bayes (12)" width="90" x="112" y="75"/>

<operator activated="true" class="weka:W-Logistic" compatibility="5.3.001" expanded="true" height="76" name="W-Logistic (8)" width="90" x="112" y="165"/>

<operator activated="true" class="weka:W-J48" compatibility="5.3.001" expanded="true" height="76" name="W-J48 (9)" width="90" x="112" y="255"/>

<connect from_port="training set 1" to_op="Naive Bayes (12)" to_port="training set"/>

<connect from_port="training set 2" to_op="W-Logistic (8)" to_port="training set"/>

<connect from_port="training set 3" to_op="W-J48 (9)" to_port="training set"/>

<connect from_op="Naive Bayes (12)" from_port="model" to_port="base model 1"/>

<connect from_op="W-Logistic (8)" from_port="model" to_port="base model 2"/>

<connect from_op="W-J48 (9)" from_port="model" to_port="base model 3"/>

<portSpacing port="source_training set 1" spacing="0"/>

<portSpacing port="source_training set 2" spacing="0"/>

<portSpacing port="source_training set 3" spacing="0"/>

<portSpacing port="source_training set 4" spacing="0"/>

<portSpacing port="sink_base model 1" spacing="0"/>

<portSpacing port="sink_base model 2" spacing="0"/>

<portSpacing port="sink_base model 3" spacing="0"/>

<portSpacing port="sink_base model 4" spacing="0"/>

</process>

<process expanded="true">

<operator activated="true" class="weka:W-J48" compatibility="5.3.001" expanded="true" height="76" name="W-J48 (10)" width="90" x="112" y="75"/>

<connect from_port="stacking examples" to_op="W-J48 (10)" to_port="training set"/>

<connect from_op="W-J48 (10)" from_port="model" to_port="stacking model"/>

<portSpacing port="source_stacking examples" spacing="0"/>

<portSpacing port="sink_stacking model" spacing="0"/>

</process>

</operator>

<connect from_port="input 1" to_op="Stacking (2)" to_port="training set"/>

<connect from_op="Stacking (2)" from_port="model" to_port="output 1"/>

<portSpacing port="source_input 1" spacing="0"/>

<portSpacing port="source_input 2" spacing="0"/>

<portSpacing port="sink_output 1" spacing="0"/>

<portSpacing port="sink_output 2" spacing="0"/>

</process>

<process expanded="true">

<operator activated="true" class="multiply" compatibility="6.5.002" expanded="true" height="94" name="Multiply (7)" width="90" x="45" y="165"/>

<operator activated="true" class="optimize_parameters_evolutionary" compatibility="6.5.002" expanded="true" height="112" name="Optimize SVM (2)" width="90" x="112" y="30">

<list key="parameters">

<parameter key="SVM (Linear).C" value="[0.000000001;100000]"/>

</list>

<parameter key="max_generations" value="10"/>

<parameter key="use_early_stopping" value="true"/>

<parameter key="population_size" value="10"/>

<parameter key="use_local_random_seed" value="true"/>

<parameter key="parallelize_optimization_process" value="true"/>

<process expanded="true">

<operator activated="true" class="parallel:x_validation_parallel" compatibility="5.3.000" expanded="true" height="112" name="Validation (6)" width="90" x="246" y="120">

<parameter key="number_of_validations" value="5"/>

<parameter key="use_local_random_seed" value="true"/>

<parameter key="parallelize_training" value="true"/>

<parameter key="parallelize_testing" value="true"/>

<process expanded="true">

<operator activated="true" class="support_vector_machine_linear" compatibility="6.5.002" expanded="true" height="76" name="SVM (2)" width="90" x="179" y="75">

<parameter key="C" value="6120.417050152911"/>

</operator>

<connect from_port="training" to_op="SVM (2)" to_port="training set"/>

<connect from_op="SVM (2)" from_port="model" to_port="model"/>

<portSpacing port="source_training" spacing="0"/>

<portSpacing port="sink_model" spacing="0"/>

<portSpacing port="sink_through 1" spacing="0"/>

</process>

<process expanded="true">

<operator activated="true" class="apply_model" compatibility="6.5.002" expanded="true" height="76" name="Apply Model (8)" width="90" x="112" y="120">

<list key="application_parameters"/>

</operator>

<operator activated="true" class="subprocess" compatibility="6.5.002" expanded="true" height="76" name="AUPRC Optim param I (2)" width="90" x="246" y="120">

<process expanded="true">

<operator activated="true" class="rename" compatibility="6.5.002" expanded="true" height="76" name="Rename (4)" width="90" x="45" y="30">

<parameter key="old_name" value="confidence(N)"/>

<parameter key="new_name" value="noconf"/>

<list key="rename_additional_attributes">

<parameter key="confidence(Y)" value="yesconf"/>

</list>

</operator>

<operator activated="true" class="r_scripting:execute_r" compatibility="6.5.000" expanded="true" height="76" name="Execute R (5)" width="90" x="179" y="30">

<parameter key="script" value="# rm_main is a mandatory function, &#10;# the number of arguments has to be the number of input ports (can be none)&#10;rm_main = function(data)&#10;{&#10; library(PRROC)&#10; data$class[data$icustay_expire_flg== &quot;Y&quot;]&lt;-1 &#10; data$class[data$icustay_expire_flg== &quot;N&quot;]&lt;-0&#10; data$class &lt;- as.numeric(data$class)&#10; x=pr.curve(scores.class0 = data$yesconf, weights.class0 = data$class)&#10; #y=roc.curve(scores.class0 = data$yesconf, weights.class0 = data$class)&#10; &#10; #a&lt;-x$auc.integral&#10; data$AUPRC&lt;- x$auc.integral&#10; #data$AUC &lt;- y$auc.integral&#10; metaData$data$AUPRC &lt;&lt;- list(type=&quot;numeric&quot;, role=&quot;performance&quot;)&#10; #metaData$data$AUC &lt;&lt;- list(type=&quot;numeric&quot;, role=&quot;performance&quot;)&#10; return(data)&#10;}&#10;"/>

</operator>

<operator activated="true" class="extract_performance" compatibility="6.5.002" expanded="true" height="76" name="AUPRC (4)" width="90" x="313" y="30">

<parameter key="performance_type" value="data_value"/>

<parameter key="attribute_name" value="AUPRC"/>

<parameter key="example_index" value="1"/>

</operator>

<connect from_port="in 1" to_op="Rename (4)" to_port="example set input"/>

<connect from_op="Rename (4)" from_port="example set output" to_op="Execute R (5)" to_port="input 1"/>

<connect from_op="Execute R (5)" from_port="output 1" to_op="AUPRC (4)" to_port="example set"/>

<connect from_op="AUPRC (4)" from_port="performance" to_port="out 1"/>

<portSpacing port="source_in 1" spacing="0"/>

<portSpacing port="source_in 2" spacing="0"/>

<portSpacing port="sink_out 1" spacing="0"/>

<portSpacing port="sink_out 2" spacing="0"/>

</process>

</operator>

<operator activated="false" class="performance_binominal_classification" compatibility="6.5.002" expanded="true" height="76" name="Performance (3)" width="90" x="246" y="300">

<parameter key="accuracy" value="false"/>

<parameter key="f_measure" value="true"/>

</operator>

<connect from_port="model" to_op="Apply Model (8)" to_port="model"/>

<connect from_port="test set" to_op="Apply Model (8)" to_port="unlabelled data"/>

<connect from_op="Apply Model (8)" from_port="labelled data" to_op="AUPRC Optim param I (2)" to_port="in 1"/>

<connect from_op="AUPRC Optim param I (2)" from_port="out 1" to_port="averagable 1"/>

<portSpacing port="source_model" spacing="0"/>

<portSpacing port="source_test set" spacing="0"/>

<portSpacing port="source_through 1" spacing="0"/>

<portSpacing port="sink_averagable 1" spacing="0"/>

<portSpacing port="sink_averagable 2" spacing="0"/>

</process>

</operator>

<connect from_port="input 1" to_op="Validation (6)" to_port="training"/>

<connect from_op="Validation (6)" from_port="model" to_port="result 1"/>

<connect from_op="Validation (6)" from_port="averagable 1" to_port="performance"/>

<portSpacing port="source_input 1" spacing="0"/>

<portSpacing port="source_input 2" spacing="0"/>

<portSpacing port="sink_performance" spacing="0"/>

<portSpacing port="sink_result 1" spacing="0"/>

<portSpacing port="sink_result 2" spacing="0"/>

</process>

</operator>

<operator activated="true" class="set_parameters" compatibility="6.5.002" expanded="true" height="76" name="Set Parameters (3)" width="90" x="246" y="165">

<list key="name_map">

<parameter key="SVM (Linear)" value=" Optimal SVM Linear"/>

</list>

</operator>

<operator activated="true" class="support_vector_machine_linear" compatibility="6.5.002" expanded="true" height="76" name="Optimal SVM Linear (2)" width="90" x="179" y="300"/>

<connect from_port="input 1" to_op="Multiply (7)" to_port="input"/>

<connect from_op="Multiply (7)" from_port="output 1" to_op="Optimize SVM (2)" to_port="input 1"/>

<connect from_op="Multiply (7)" from_port="output 2" to_op="Optimal SVM Linear (2)" to_port="training set"/>

<connect from_op="Optimize SVM (2)" from_port="parameter" to_op="Set Parameters (3)" to_port="parameter set"/>

<connect from_op="Optimal SVM Linear (2)" from_port="model" to_port="output 1"/>

<portSpacing port="source_input 1" spacing="0"/>

<portSpacing port="source_input 2" spacing="0"/>

<portSpacing port="sink_output 1" spacing="0"/>

<portSpacing port="sink_output 2" spacing="0"/>

</process>

<process expanded="true">

<operator activated="true" class="multiply" compatibility="6.5.002" expanded="true" height="94" name="Multiply (8)" width="90" x="45" y="165"/>

<operator activated="true" class="support_vector_machine_libsvm" compatibility="6.5.002" expanded="true" height="76" name="Optimal SVM rbf (2)" width="90" x="246" y="300">

<parameter key="C" value="68146.13250384372"/>

<parameter key="nu" value="0.12334713683253792"/>

<list key="class_weights"/>

</operator>

<operator activated="true" class="optimize_parameters_evolutionary" compatibility="6.5.002" expanded="true" height="112" name="Optimize Parameters (4)" width="90" x="112" y="30">

<list key="parameters">

<parameter key="SVM (rbf).C" value="[0.000000001;100000]"/>

<parameter key="SVM (rbf).nu" value="[0.001;2]"/>

</list>

<parameter key="max_generations" value="10"/>

<parameter key="use_early_stopping" value="true"/>

<parameter key="population_size" value="10"/>

<parameter key="use_local_random_seed" value="true"/>

<parameter key="parallelize_optimization_process" value="true"/>

<process expanded="true">

<operator activated="true" class="parallel:x_validation_parallel" compatibility="5.3.000" expanded="true" height="112" name="Validation (8)" width="90" x="380" y="120">

<parameter key="number_of_validations" value="5"/>

<parameter key="use_local_random_seed" value="true"/>

<parameter key="parallelize_training" value="true"/>

<parameter key="parallelize_testing" value="true"/>

<process expanded="true">

<operator activated="true" class="support_vector_machine_libsvm" compatibility="6.5.002" expanded="true" height="76" name="SVM (4)" width="90" x="179" y="75">

<parameter key="C" value="68146.13250384372"/>

<parameter key="nu" value="0.12334713683253792"/>

<list key="class_weights"/>

</operator>

<connect from_port="training" to_op="SVM (4)" to_port="training set"/>

<connect from_op="SVM (4)" from_port="model" to_port="model"/>

<portSpacing port="source_training" spacing="0"/>

<portSpacing port="sink_model" spacing="0"/>

<portSpacing port="sink_through 1" spacing="0"/>

</process>

<process expanded="true">

<operator activated="true" class="apply_model" compatibility="6.5.002" expanded="true" height="76" name="Apply Model (10)" width="90" x="112" y="120">

<list key="application_parameters"/>

</operator>

<operator activated="true" class="subprocess" compatibility="6.5.002" expanded="true" height="76" name="AUPRC Param Optim II (2)" width="90" x="246" y="120">

<process expanded="true">

<operator activated="true" class="rename" compatibility="6.5.002" expanded="true" height="76" name="Rename (5)" width="90" x="45" y="30">

<parameter key="old_name" value="confidence(N)"/>

<parameter key="new_name" value="noconf"/>

<list key="rename_additional_attributes">

<parameter key="confidence(Y)" value="yesconf"/>

</list>

</operator>

<operator activated="true" class="r_scripting:execute_r" compatibility="6.5.000" expanded="true" height="76" name="Execute R (6)" width="90" x="179" y="30">

<parameter key="script" value="# rm_main is a mandatory function, &#10;# the number of arguments has to be the number of input ports (can be none)&#10;rm_main = function(data)&#10;{&#10; library(PRROC)&#10; data$class[data$icustay_expire_flg== &quot;Y&quot;]&lt;-1 &#10; data$class[data$icustay_expire_flg== &quot;N&quot;]&lt;-0&#10; data$class &lt;- as.numeric(data$class)&#10; x=pr.curve(scores.class0 = data$yesconf, weights.class0 = data$class)&#10; #y=roc.curve(scores.class0 = data$yesconf, weights.class0 = data$class)&#10; &#10; #a&lt;-x$auc.integral&#10; data$AUPRC&lt;- x$auc.integral&#10; #data$AUC &lt;- y$auc.integral&#10; metaData$data$AUPRC &lt;&lt;- list(type=&quot;numeric&quot;, role=&quot;performance&quot;)&#10; #metaData$data$AUC &lt;&lt;- list(type=&quot;numeric&quot;, role=&quot;performance&quot;)&#10; return(data)&#10;}&#10;"/>

</operator>

<operator activated="true" class="extract_performance" compatibility="6.5.002" expanded="true" height="76" name="AUPRC (5)" width="90" x="313" y="30">

<parameter key="performance_type" value="data_value"/>

<parameter key="attribute_name" value="AUPRC"/>

<parameter key="example_index" value="1"/>

</operator>

<connect from_port="in 1" to_op="Rename (5)" to_port="example set input"/>

<connect from_op="Rename (5)" from_port="example set output" to_op="Execute R (6)" to_port="input 1"/>

<connect from_op="Execute R (6)" from_port="output 1" to_op="AUPRC (5)" to_port="example set"/>

<connect from_op="AUPRC (5)" from_port="performance" to_port="out 1"/>

<portSpacing port="source_in 1" spacing="0"/>

<portSpacing port="source_in 2" spacing="0"/>

<portSpacing port="sink_out 1" spacing="0"/>

<portSpacing port="sink_out 2" spacing="0"/>

</process>

</operator>

<operator activated="false" class="performance_binominal_classification" compatibility="6.5.002" expanded="true" height="76" name="Performance (7)" width="90" x="246" y="345">

<parameter key="accuracy" value="false"/>

<parameter key="f_measure" value="true"/>

</operator>

<connect from_port="model" to_op="Apply Model (10)" to_port="model"/>

<connect from_port="test set" to_op="Apply Model (10)" to_port="unlabelled data"/>

<connect from_op="Apply Model (10)" from_port="labelled data" to_op="AUPRC Param Optim II (2)" to_port="in 1"/>

<connect from_op="AUPRC Param Optim II (2)" from_port="out 1" to_port="averagable 1"/>

<portSpacing port="source_model" spacing="0"/>

<portSpacing port="source_test set" spacing="0"/>

<portSpacing port="source_through 1" spacing="0"/>

<portSpacing port="sink_averagable 1" spacing="0"/>

<portSpacing port="sink_averagable 2" spacing="0"/>

</process>

</operator>

<connect from_port="input 1" to_op="Validation (8)" to_port="training"/>

<connect from_op="Validation (8)" from_port="model" to_port="result 1"/>

<connect from_op="Validation (8)" from_port="averagable 1" to_port="performance"/>

<portSpacing port="source_input 1" spacing="0"/>

<portSpacing port="source_input 2" spacing="0"/>

<portSpacing port="sink_performance" spacing="0"/>

<portSpacing port="sink_result 1" spacing="0"/>

<portSpacing port="sink_result 2" spacing="0"/>

</process>

</operator>

<operator activated="true" class="set_parameters" compatibility="6.5.002" expanded="true" height="76" name="Set Parameters (4)" width="90" x="246" y="120">

<list key="name_map">

<parameter key="SVM (rbf)" value="Optimal SVM rbf"/>

</list>

</operator>

<connect from_port="input 1" to_op="Multiply (8)" to_port="input"/>

<connect from_op="Multiply (8)" from_port="output 1" to_op="Optimize Parameters (4)" to_port="input 1"/>

<connect from_op="Multiply (8)" from_port="output 2" to_op="Optimal SVM rbf (2)" to_port="training set"/>

<connect from_op="Optimal SVM rbf (2)" from_port="model" to_port="output 1"/>

<connect from_op="Optimize Parameters (4)" from_port="parameter" to_op="Set Parameters (4)" to_port="parameter set"/>

<portSpacing port="source_input 1" spacing="0"/>

<portSpacing port="source_input 2" spacing="0"/>

<portSpacing port="sink_output 1" spacing="0"/>

<portSpacing port="sink_output 2" spacing="0"/>

</process>

<process expanded="true">

<operator activated="true" class="weka:W-SMO" compatibility="5.3.001" expanded="true" height="76" name="W-SMO (2)" width="90" x="45" y="120"/>

<connect from_port="input 1" to_op="W-SMO (2)" to_port="training set"/>

<connect from_op="W-SMO (2)" from_port="model" to_port="output 1"/>

<portSpacing port="source_input 1" spacing="0"/>

<portSpacing port="source_input 2" spacing="0"/>

<portSpacing port="sink_output 1" spacing="0"/>

<portSpacing port="sink_output 2" spacing="0"/>

</process>

</operator>

<connect from_port="training" to_op="OptimizeK" to_port="example set input"/>

<connect from_op="Retrieve" from_port="output" to_op="OptimizeK" to_port="weights"/>

<connect from_op="OptimizeK" from_port="example set output" to_op="Algorithms (2)" to_port="input 1"/>

<connect from_op="Algorithms (2)" from_port="output 1" to_port="model"/>

<portSpacing port="source_training" spacing="0"/>

<portSpacing port="sink_model" spacing="0"/>

<portSpacing port="sink_through 1" spacing="0"/>

</process>

<process expanded="true">

<operator activated="true" class="apply_model" compatibility="6.5.002" expanded="true" height="76" name="Apply Model (11)" width="90" x="112" y="120">

<list key="application_parameters"/>

</operator>

<operator activated="true" class="subprocess" compatibility="6.5.002" expanded="true" height="76" name="Subprocess (3)" width="90" x="246" y="120">

<parameter key="parallelize_nested_process" value="true"/>

<process expanded="true">

<operator activated="true" class="rename" compatibility="6.5.002" expanded="true" height="76" name="Rename (6)" width="90" x="45" y="30">

<parameter key="old_name" value="confidence(N)"/>

<parameter key="new_name" value="noconf"/>

<list key="rename_additional_attributes">

<parameter key="confidence(Y)" value="yesconf"/>

</list>

</operator>

<operator activated="true" class="r_scripting:execute_r" compatibility="6.5.000" expanded="true" height="76" name="Execute R (7)" width="90" x="179" y="30">

<parameter key="script" value="# rm_main is a mandatory function, &#10;# the number of arguments has to be the number of input ports (can be none)&#10;rm_main = function(data)&#10;{&#10; library(PRROC)&#10; data$class[data$icustay_expire_flg== &quot;Y&quot;]&lt;-1 &#10; data$class[data$icustay_expire_flg== &quot;N&quot;]&lt;-0&#10; data$class &lt;- as.numeric(data$class)&#10; x=pr.curve(scores.class0 = data$yesconf, weights.class0 = data$class)&#10; #y=roc.curve(scores.class0 = data$yesconf, weights.class0 = data$class)&#10; &#10; #a&lt;-x$auc.integral&#10; data$AUPRC&lt;- x$auc.integral&#10; #data$AUC &lt;- y$auc.integral&#10; metaData$data$AUPRC &lt;&lt;- list(type=&quot;numeric&quot;, role=&quot;performance&quot;)&#10; #metaData$data$AUC &lt;&lt;- list(type=&quot;numeric&quot;, role=&quot;performance&quot;)&#10; return(data)&#10;}&#10;"/>

</operator>

<operator activated="true" class="extract_performance" compatibility="6.5.002" expanded="true" height="76" name="AUPRC (6)" width="90" x="313" y="30">

<parameter key="performance_type" value="data_value"/>

<parameter key="attribute_name" value="AUPRC"/>

<parameter key="example_index" value="1"/>

</operator>

<connect from_port="in 1" to_op="Rename (6)" to_port="example set input"/>

<connect from_op="Rename (6)" from_port="example set output" to_op="Execute R (7)" to_port="input 1"/>

<connect from_op="Execute R (7)" from_port="output 1" to_op="AUPRC (6)" to_port="example set"/>

<connect from_op="AUPRC (6)" from_port="performance" to_port="out 1"/>

<portSpacing port="source_in 1" spacing="0"/>

<portSpacing port="source_in 2" spacing="0"/>

<portSpacing port="sink_out 1" spacing="0"/>

<portSpacing port="sink_out 2" spacing="0"/>

</process>

</operator>

<connect from_port="model" to_op="Apply Model (11)" to_port="model"/>

<connect from_port="test set" to_op="Apply Model (11)" to_port="unlabelled data"/>

<connect from_op="Apply Model (11)" from_port="labelled data" to_op="Subprocess (3)" to_port="in 1"/>

<connect from_op="Subprocess (3)" from_port="out 1" to_port="averagable 1"/>

<portSpacing port="source_model" spacing="0"/>

<portSpacing port="source_test set" spacing="0"/>

<portSpacing port="source_through 1" spacing="0"/>

<portSpacing port="sink_averagable 1" spacing="0"/>

<portSpacing port="sink_averagable 2" spacing="0"/>

</process>

</operator>

<connect from_port="input 1" to_op="Validation (4)" to_port="training"/>

<connect from_op="Validation (4)" from_port="averagable 1" to_port="performance"/>

<portSpacing port="source_input 1" spacing="0"/>

<portSpacing port="source_input 2" spacing="0"/>

<portSpacing port="sink_performance" spacing="0"/>

<portSpacing port="sink_result 1" spacing="0"/>

</process>

</operator>

<operator activated="true" class="set_parameters" compatibility="6.5.002" expanded="true" height="76" name="Set Parameters (5)" width="90" x="447" y="255">

<list key="name_map">

<parameter key="OptimizeK" value="OptimalWeights"/>

</list>

</operator>

<operator activated="true" class="select_by_weights" compatibility="6.5.002" expanded="true" height="94" name="OptimalWeights" width="90" x="447" y="30">

<parameter key="weight_relation" value="top k"/>

<parameter key="k" value="45"/>

</operator>

<operator activated="true" class="multiply" compatibility="6.5.002" expanded="true" height="94" name="Multiply" width="90" x="581" y="165"/>

<operator activated="true" class="select_subprocess" compatibility="6.5.002" expanded="true" height="94" name="Algorithms (3)" width="90" x="648" y="30">

<parameter key="select_which" value="%{iteration}"/>

<parameter key="parallelize_selection_1" value="true"/>

<parameter key="parallelize_selection_2" value="true"/>

<process expanded="true">

<operator activated="true" class="weka:W-DecisionStump" compatibility="5.3.001" expanded="true" height="76" name="W-DecisionStump (3)" width="90" x="45" y="30"/>

<connect from_port="input 1" to_op="W-DecisionStump (3)" to_port="training set"/>

<connect from_op="W-DecisionStump (3)" from_port="model" to_port="output 1"/>

<connect from_op="W-DecisionStump (3)" from_port="exampleSet" to_port="output 2"/>

<portSpacing port="source_input 1" spacing="0"/>

<portSpacing port="source_input 2" spacing="0"/>

<portSpacing port="source_input 3" spacing="0"/>

<portSpacing port="sink_output 1" spacing="0"/>

<portSpacing port="sink_output 2" spacing="0"/>

<portSpacing port="sink_output 3" spacing="0"/>

</process>

<process expanded="true">

<operator activated="true" class="decision_stump" compatibility="6.5.002" expanded="true" height="76" name="Decision Stump (7)" width="90" x="45" y="30"/>

<connect from_port="input 1" to_op="Decision Stump (7)" to_port="training set"/>

<connect from_op="Decision Stump (7)" from_port="model" to_port="output 1"/>

<connect from_op="Decision Stump (7)" from_port="exampleSet" to_port="output 2"/>

<portSpacing port="source_input 1" spacing="0"/>

<portSpacing port="source_input 2" spacing="0"/>

<portSpacing port="source_input 3" spacing="0"/>

<portSpacing port="sink_output 1" spacing="0"/>

<portSpacing port="sink_output 2" spacing="0"/>

<portSpacing port="sink_output 3" spacing="0"/>

</process>

<process expanded="true">

<operator activated="true" class="weka:W-J48" compatibility="5.3.001" expanded="true" height="76" name="W-J48 (11)" width="90" x="112" y="30"/>

<connect from_port="input 1" to_op="W-J48 (11)" to_port="training set"/>

<connect from_op="W-J48 (11)" from_port="model" to_port="output 1"/>

<connect from_op="W-J48 (11)" from_port="exampleSet" to_port="output 2"/>

<portSpacing port="source_input 1" spacing="0"/>

<portSpacing port="source_input 2" spacing="0"/>

<portSpacing port="source_input 3" spacing="0"/>

<portSpacing port="sink_output 1" spacing="0"/>

<portSpacing port="sink_output 2" spacing="0"/>

<portSpacing port="sink_output 3" spacing="0"/>

</process>

<process expanded="true">

<operator activated="true" class="naive_bayes" compatibility="6.5.002" expanded="true" height="76" name="Naive Bayes (13)" width="90" x="112" y="30"/>

<connect from_port="input 1" to_op="Naive Bayes (13)" to_port="training set"/>

<connect from_op="Naive Bayes (13)" from_port="model" to_port="output 1"/>

<connect from_op="Naive Bayes (13)" from_port="exampleSet" to_port="output 2"/>

<portSpacing port="source_input 1" spacing="0"/>

<portSpacing port="source_input 2" spacing="0"/>

<portSpacing port="source_input 3" spacing="0"/>

<portSpacing port="sink_output 1" spacing="0"/>

<portSpacing port="sink_output 2" spacing="0"/>

<portSpacing port="sink_output 3" spacing="0"/>

</process>

<process expanded="true">

<operator activated="true" class="weka:W-Logistic" compatibility="5.3.001" expanded="true" height="76" name="W-Logistic (9)" width="90" x="45" y="30"/>

<connect from_port="input 1" to_op="W-Logistic (9)" to_port="training set"/>

<connect from_op="W-Logistic (9)" from_port="model" to_port="output 1"/>

<connect from_op="W-Logistic (9)" from_port="exampleSet" to_port="output 2"/>

<portSpacing port="source_input 1" spacing="0"/>

<portSpacing port="source_input 2" spacing="0"/>

<portSpacing port="source_input 3" spacing="0"/>

<portSpacing port="sink_output 1" spacing="0"/>

<portSpacing port="sink_output 2" spacing="0"/>

<portSpacing port="sink_output 3" spacing="0"/>

</process>

<process expanded="true">

<operator activated="true" class="parallel_random_forest" compatibility="6.5.002" expanded="true" height="76" name="RF - Rapid (3)" width="90" x="45" y="30">

<parameter key="number_of_trees" value="30"/>

</operator>

<connect from_port="input 1" to_op="RF - Rapid (3)" to_port="training set"/>

<connect from_op="RF - Rapid (3)" from_port="model" to_port="output 1"/>

<connect from_op="RF - Rapid (3)" from_port="exampleSet" to_port="output 2"/>

<portSpacing port="source_input 1" spacing="0"/>

<portSpacing port="source_input 2" spacing="0"/>

<portSpacing port="source_input 3" spacing="0"/>

<portSpacing port="sink_output 1" spacing="0"/>

<portSpacing port="sink_output 2" spacing="0"/>

<portSpacing port="sink_output 3" spacing="0"/>

</process>

<process expanded="true">

<operator activated="true" class="weka:W-RandomForest" compatibility="5.3.001" expanded="true" height="76" name="RF - Weka (3)" width="90" x="45" y="30"/>

<connect from_port="input 1" to_op="RF - Weka (3)" to_port="training set"/>

<connect from_op="RF - Weka (3)" from_port="model" to_port="output 1"/>

<connect from_op="RF - Weka (3)" from_port="exampleSet" to_port="output 2"/>

<portSpacing port="source_input 1" spacing="0"/>

<portSpacing port="source_input 2" spacing="0"/>

<portSpacing port="source_input 3" spacing="0"/>

<portSpacing port="sink_output 1" spacing="0"/>

<portSpacing port="sink_output 2" spacing="0"/>

<portSpacing port="sink_output 3" spacing="0"/>

</process>

<process expanded="true">

<operator activated="true" class="adaboost" compatibility="6.5.002" expanded="true" height="76" name="AdaBoost (6)" width="90" x="45" y="30">

<parameter key="parallelize_learning_process" value="true"/>

<process expanded="true">

<operator activated="true" class="decision_stump" compatibility="6.5.002" expanded="true" height="76" name="Decision Stump (8)" width="90" x="112" y="75"/>

<connect from_port="training set" to_op="Decision Stump (8)" to_port="training set"/>

<connect from_op="Decision Stump (8)" from_port="model" to_port="model"/>

<portSpacing port="source_training set" spacing="0"/>

<portSpacing port="sink_model" spacing="0"/>

</process>

</operator>

<connect from_port="input 1" to_op="AdaBoost (6)" to_port="training set"/>

<connect from_op="AdaBoost (6)" from_port="model" to_port="output 1"/>

<connect from_op="AdaBoost (6)" from_port="example set" to_port="output 2"/>

<portSpacing port="source_input 1" spacing="0"/>

<portSpacing port="source_input 2" spacing="0"/>

<portSpacing port="source_input 3" spacing="0"/>

<portSpacing port="sink_output 1" spacing="0"/>

<portSpacing port="sink_output 2" spacing="0"/>

<portSpacing port="sink_output 3" spacing="0"/>

</process>

<process expanded="true">

<operator activated="true" class="adaboost" compatibility="6.5.002" expanded="true" height="76" name="AdaBoost (7)" width="90" x="45" y="30">

<parameter key="parallelize_learning_process" value="true"/>

<process expanded="true">

<operator activated="true" class="weka:W-J48" compatibility="5.3.001" expanded="true" height="76" name="W-J48 (12)" width="90" x="380" y="75"/>

<connect from_port="training set" to_op="W-J48 (12)" to_port="training set"/>

<connect from_op="W-J48 (12)" from_port="model" to_port="model"/>

<portSpacing port="source_training set" spacing="0"/>

<portSpacing port="sink_model" spacing="0"/>

</process>

</operator>

<connect from_port="input 1" to_op="AdaBoost (7)" to_port="training set"/>

<connect from_op="AdaBoost (7)" from_port="model" to_port="output 1"/>

<connect from_op="AdaBoost (7)" from_port="example set" to_port="output 2"/>

<portSpacing port="source_input 1" spacing="0"/>

<portSpacing port="source_input 2" spacing="0"/>

<portSpacing port="source_input 3" spacing="0"/>

<portSpacing port="sink_output 1" spacing="0"/>

<portSpacing port="sink_output 2" spacing="0"/>

<portSpacing port="sink_output 3" spacing="0"/>

</process>

<process expanded="true">

<operator activated="true" class="adaboost" compatibility="6.5.002" expanded="true" height="76" name="AdaBoost (8)" width="90" x="45" y="30">

<parameter key="parallelize_learning_process" value="true"/>

<process expanded="true">

<operator activated="true" class="naive_bayes" compatibility="6.5.002" expanded="true" height="76" name="Naive Bayes (14)" width="90" x="313" y="75"/>

<connect from_port="training set" to_op="Naive Bayes (14)" to_port="training set"/>

<connect from_op="Naive Bayes (14)" from_port="model" to_port="model"/>

<portSpacing port="source_training set" spacing="0"/>

<portSpacing port="sink_model" spacing="0"/>

</process>

</operator>

<connect from_port="input 1" to_op="AdaBoost (8)" to_port="training set"/>

<connect from_op="AdaBoost (8)" from_port="model" to_port="output 1"/>

<connect from_op="AdaBoost (8)" from_port="example set" to_port="output 2"/>

<portSpacing port="source_input 1" spacing="0"/>

<portSpacing port="source_input 2" spacing="0"/>

<portSpacing port="source_input 3" spacing="0"/>

<portSpacing port="sink_output 1" spacing="0"/>

<portSpacing port="sink_output 2" spacing="0"/>

<portSpacing port="sink_output 3" spacing="0"/>

</process>

<process expanded="true">

<operator activated="true" class="adaboost" compatibility="6.5.002" expanded="true" height="76" name="AdaBoost (9)" width="90" x="45" y="30">

<parameter key="parallelize_learning_process" value="true"/>

<process expanded="true">

<operator activated="true" class="weka:W-Logistic" compatibility="5.3.001" expanded="true" height="76" name="W-Logistic (10)" width="90" x="313" y="75"/>

<connect from_port="training set" to_op="W-Logistic (10)" to_port="training set"/>

<connect from_op="W-Logistic (10)" from_port="model" to_port="model"/>

<portSpacing port="source_training set" spacing="0"/>

<portSpacing port="sink_model" spacing="0"/>

</process>

</operator>

<connect from_port="input 1" to_op="AdaBoost (9)" to_port="training set"/>

<connect from_op="AdaBoost (9)" from_port="model" to_port="output 1"/>

<connect from_op="AdaBoost (9)" from_port="example set" to_port="output 2"/>

<portSpacing port="source_input 1" spacing="0"/>

<portSpacing port="source_input 2" spacing="0"/>

<portSpacing port="source_input 3" spacing="0"/>

<portSpacing port="sink_output 1" spacing="0"/>

<portSpacing port="sink_output 2" spacing="0"/>

<portSpacing port="sink_output 3" spacing="0"/>

</process>

<process expanded="true">

<operator activated="true" class="bagging" compatibility="6.5.002" expanded="true" height="76" name="Bagging (6)" width="90" x="112" y="30">

<parameter key="sample_ratio" value="0.7"/>

<parameter key="use_local_random_seed" value="true"/>

<parameter key="parallelize_learning_process" value="true"/>

<process expanded="true">

<operator activated="true" class="decision_stump" compatibility="6.5.002" expanded="true" height="76" name="Decision Stump (9)" width="90" x="313" y="75"/>

<connect from_port="training set" to_op="Decision Stump (9)" to_port="training set"/>

<connect from_op="Decision Stump (9)" from_port="model" to_port="model"/>

<portSpacing port="source_training set" spacing="0"/>

<portSpacing port="sink_model" spacing="0"/>

</process>

</operator>

<connect from_port="input 1" to_op="Bagging (6)" to_port="training set"/>

<connect from_op="Bagging (6)" from_port="model" to_port="output 1"/>

<connect from_op="Bagging (6)" from_port="example set" to_port="output 2"/>

<portSpacing port="source_input 1" spacing="0"/>

<portSpacing port="source_input 2" spacing="0"/>

<portSpacing port="source_input 3" spacing="0"/>

<portSpacing port="sink_output 1" spacing="0"/>

<portSpacing port="sink_output 2" spacing="0"/>

<portSpacing port="sink_output 3" spacing="0"/>

</process>

<process expanded="true">

<operator activated="true" class="bagging" compatibility="6.5.002" expanded="true" height="76" name="Bagging (7)" width="90" x="45" y="30">

<parameter key="sample_ratio" value="0.7"/>

<parameter key="use_local_random_seed" value="true"/>

<parameter key="parallelize_learning_process" value="true"/>

<process expanded="true">

<operator activated="true" class="weka:W-J48" compatibility="5.3.001" expanded="true" height="76" name="W-J48 (13)" width="90" x="246" y="75"/>

<connect from_port="training set" to_op="W-J48 (13)" to_port="training set"/>

<connect from_op="W-J48 (13)" from_port="model" to_port="model"/>

<portSpacing port="source_training set" spacing="0"/>

<portSpacing port="sink_model" spacing="0"/>

</process>

</operator>

<connect from_port="input 1" to_op="Bagging (7)" to_port="training set"/>

<connect from_op="Bagging (7)" from_port="model" to_port="output 1"/>

<connect from_op="Bagging (7)" from_port="example set" to_port="output 2"/>

<portSpacing port="source_input 1" spacing="0"/>

<portSpacing port="source_input 2" spacing="0"/>

<portSpacing port="source_input 3" spacing="0"/>

<portSpacing port="sink_output 1" spacing="0"/>

<portSpacing port="sink_output 2" spacing="0"/>

<portSpacing port="sink_output 3" spacing="0"/>

</process>

<process expanded="true">

<operator activated="true" class="bagging" compatibility="6.5.002" expanded="true" height="76" name="Bagging (8)" width="90" x="85" y="30">

<parameter key="sample_ratio" value="0.7"/>

<process expanded="true">

<operator activated="true" class="naive_bayes" compatibility="6.5.002" expanded="true" height="76" name="Naive Bayes (15)" width="90" x="380" y="75"/>

<connect from_port="training set" to_op="Naive Bayes (15)" to_port="training set"/>

<connect from_op="Naive Bayes (15)" from_port="model" to_port="model"/>

<portSpacing port="source_training set" spacing="0"/>

<portSpacing port="sink_model" spacing="0"/>

</process>

</operator>

<connect from_port="input 1" to_op="Bagging (8)" to_port="training set"/>

<connect from_op="Bagging (8)" from_port="model" to_port="output 1"/>

<connect from_op="Bagging (8)" from_port="example set" to_port="output 2"/>

<portSpacing port="source_input 1" spacing="0"/>

<portSpacing port="source_input 2" spacing="0"/>

<portSpacing port="source_input 3" spacing="0"/>

<portSpacing port="sink_output 1" spacing="0"/>

<portSpacing port="sink_output 2" spacing="0"/>

<portSpacing port="sink_output 3" spacing="0"/>

</process>

<process expanded="true">

<operator activated="true" class="bagging" compatibility="6.5.002" expanded="true" height="76" name="Bagging (9)" width="90" x="45" y="30">

<parameter key="sample_ratio" value="0.7"/>

<parameter key="use_local_random_seed" value="true"/>

<parameter key="parallelize_learning_process" value="true"/>

<process expanded="true">

<operator activated="true" class="weka:W-Logistic" compatibility="5.3.001" expanded="true" height="76" name="W-Logistic (11)" width="90" x="313" y="75"/>

<connect from_port="training set" to_op="W-Logistic (11)" to_port="training set"/>

<connect from_op="W-Logistic (11)" from_port="model" to_port="model"/>

<portSpacing port="source_training set" spacing="0"/>

<portSpacing port="sink_model" spacing="0"/>

</process>

</operator>

<connect from_port="input 1" to_op="Bagging (9)" to_port="training set"/>

<connect from_op="Bagging (9)" from_port="model" to_port="output 1"/>

<connect from_op="Bagging (9)" from_port="example set" to_port="output 2"/>

<portSpacing port="source_input 1" spacing="0"/>

<portSpacing port="source_input 2" spacing="0"/>

<portSpacing port="source_input 3" spacing="0"/>

<portSpacing port="sink_output 1" spacing="0"/>

<portSpacing port="sink_output 2" spacing="0"/>

<portSpacing port="sink_output 3" spacing="0"/>

</process>

<process expanded="true">

<operator activated="true" class="stacking" compatibility="6.5.002" expanded="true" height="60" name="Stacking (3)" width="90" x="45" y="30">

<parameter key="parallelize_base_learner" value="true"/>

<parameter key="parallelize_stacking_model_learner" value="true"/>

<process expanded="true">

<operator activated="true" class="naive_bayes" compatibility="6.5.002" expanded="true" height="76" name="Naive Bayes (16)" width="90" x="112" y="75"/>

<operator activated="true" class="weka:W-Logistic" compatibility="5.3.001" expanded="true" height="76" name="W-Logistic (12)" width="90" x="112" y="210"/>

<operator activated="true" class="weka:W-J48" compatibility="5.3.001" expanded="true" height="76" name="W-J48 (14)" width="90" x="112" y="300"/>

<connect from_port="training set 1" to_op="Naive Bayes (16)" to_port="training set"/>

<connect from_port="training set 2" to_op="W-Logistic (12)" to_port="training set"/>

<connect from_port="training set 3" to_op="W-J48 (14)" to_port="training set"/>

<connect from_op="Naive Bayes (16)" from_port="model" to_port="base model 1"/>

<connect from_op="W-Logistic (12)" from_port="model" to_port="base model 2"/>

<connect from_op="W-J48 (14)" from_port="model" to_port="base model 3"/>

<portSpacing port="source_training set 1" spacing="0"/>

<portSpacing port="source_training set 2" spacing="0"/>

<portSpacing port="source_training set 3" spacing="0"/>

<portSpacing port="source_training set 4" spacing="0"/>

<portSpacing port="sink_base model 1" spacing="0"/>

<portSpacing port="sink_base model 2" spacing="0"/>

<portSpacing port="sink_base model 3" spacing="0"/>

<portSpacing port="sink_base model 4" spacing="0"/>

</process>

<process expanded="true">

<operator activated="true" class="weka:W-J48" compatibility="5.3.001" expanded="true" height="76" name="W-J48 (15)" width="90" x="112" y="75"/>

<connect from_port="stacking examples" to_op="W-J48 (15)" to_port="training set"/>

<connect from_op="W-J48 (15)" from_port="model" to_port="stacking model"/>

<portSpacing port="source_stacking examples" spacing="0"/>

<portSpacing port="sink_stacking model" spacing="0"/>

</process>

</operator>

<connect from_port="input 1" to_op="Stacking (3)" to_port="training set"/>

<connect from_port="input 2" to_port="output 2"/>

<connect from_op="Stacking (3)" from_port="model" to_port="output 1"/>

<portSpacing port="source_input 1" spacing="0"/>

<portSpacing port="source_input 2" spacing="0"/>

<portSpacing port="source_input 3" spacing="0"/>

<portSpacing port="sink_output 1" spacing="0"/>

<portSpacing port="sink_output 2" spacing="0"/>

<portSpacing port="sink_output 3" spacing="0"/>

</process>

<process expanded="true">

<operator activated="true" class="multiply" compatibility="6.5.002" expanded="true" height="94" name="Multiply (9)" width="90" x="45" y="165"/>

<operator activated="true" class="optimize_parameters_evolutionary" compatibility="6.5.002" expanded="true" height="112" name="Optimize SVM (3)" width="90" x="112" y="30">

<list key="parameters">

<parameter key="SVM (Linear).C" value="[0.000000001;100000]"/>

</list>

<parameter key="max_generations" value="10"/>

<parameter key="use_early_stopping" value="true"/>

<parameter key="population_size" value="10"/>

<parameter key="use_local_random_seed" value="true"/>

<parameter key="parallelize_optimization_process" value="true"/>

<process expanded="true">

<operator activated="true" class="parallel:x_validation_parallel" compatibility="5.3.000" expanded="true" height="112" name="Validation (9)" width="90" x="246" y="120">

<parameter key="number_of_validations" value="5"/>

<parameter key="use_local_random_seed" value="true"/>

<parameter key="parallelize_training" value="true"/>

<parameter key="parallelize_testing" value="true"/>

<process expanded="true">

<operator activated="true" class="support_vector_machine_linear" compatibility="6.5.002" expanded="true" height="76" name="SVM (5)" width="90" x="179" y="75">

<parameter key="C" value="6120.417050152911"/>

</operator>

<connect from_port="training" to_op="SVM (5)" to_port="training set"/>

<connect from_op="SVM (5)" from_port="model" to_port="model"/>

<portSpacing port="source_training" spacing="0"/>

<portSpacing port="sink_model" spacing="0"/>

<portSpacing port="sink_through 1" spacing="0"/>

</process>

<process expanded="true">

<operator activated="true" class="apply_model" compatibility="6.5.002" expanded="true" height="76" name="Apply Model (12)" width="90" x="112" y="120">

<list key="application_parameters"/>

</operator>

<operator activated="true" class="subprocess" compatibility="6.5.002" expanded="true" height="76" name="AUPRC Optim param I (3)" width="90" x="246" y="120">

<process expanded="true">

<operator activated="true" class="rename" compatibility="6.5.002" expanded="true" height="76" name="Rename (7)" width="90" x="45" y="30">

<parameter key="old_name" value="confidence(N)"/>

<parameter key="new_name" value="noconf"/>

<list key="rename_additional_attributes">

<parameter key="confidence(Y)" value="yesconf"/>

</list>

</operator>

<operator activated="true" class="r_scripting:execute_r" compatibility="6.5.000" expanded="true" height="76" name="Execute R (8)" width="90" x="179" y="30">

<parameter key="script" value="# rm_main is a mandatory function, &#10;# the number of arguments has to be the number of input ports (can be none)&#10;rm_main = function(data)&#10;{&#10; library(PRROC)&#10; data$class[data$icustay_expire_flg== &quot;Y&quot;]&lt;-1 &#10; data$class[data$icustay_expire_flg== &quot;N&quot;]&lt;-0&#10; data$class &lt;- as.numeric(data$class)&#10; x=pr.curve(scores.class0 = data$yesconf, weights.class0 = data$class)&#10; #y=roc.curve(scores.class0 = data$yesconf, weights.class0 = data$class)&#10; &#10; #a&lt;-x$auc.integral&#10; data$AUPRC&lt;- x$auc.integral&#10; #data$AUC &lt;- y$auc.integral&#10; metaData$data$AUPRC &lt;&lt;- list(type=&quot;numeric&quot;, role=&quot;performance&quot;)&#10; #metaData$data$AUC &lt;&lt;- list(type=&quot;numeric&quot;, role=&quot;performance&quot;)&#10; return(data)&#10;}&#10;"/>

</operator>

<operator activated="true" class="extract_performance" compatibility="6.5.002" expanded="true" height="76" name="AUPRC (7)" width="90" x="313" y="30">

<parameter key="performance_type" value="data_value"/>

<parameter key="attribute_name" value="AUPRC"/>

<parameter key="example_index" value="1"/>

</operator>

<connect from_port="in 1" to_op="Rename (7)" to_port="example set input"/>

<connect from_op="Rename (7)" from_port="example set output" to_op="Execute R (8)" to_port="input 1"/>

<connect from_op="Execute R (8)" from_port="output 1" to_op="AUPRC (7)" to_port="example set"/>

<connect from_op="AUPRC (7)" from_port="performance" to_port="out 1"/>

<portSpacing port="source_in 1" spacing="0"/>

<portSpacing port="source_in 2" spacing="0"/>

<portSpacing port="sink_out 1" spacing="0"/>

<portSpacing port="sink_out 2" spacing="0"/>

</process>

</operator>

<operator activated="false" class="performance_binominal_classification" compatibility="6.5.002" expanded="true" height="76" name="Performance (9)" width="90" x="246" y="300">

<parameter key="accuracy" value="false"/>

<parameter key="f_measure" value="true"/>

</operator>

<connect from_port="model" to_op="Apply Model (12)" to_port="model"/>

<connect from_port="test set" to_op="Apply Model (12)" to_port="unlabelled data"/>

<connect from_op="Apply Model (12)" from_port="labelled data" to_op="AUPRC Optim param I (3)" to_port="in 1"/>

<connect from_op="AUPRC Optim param I (3)" from_port="out 1" to_port="averagable 1"/>

<portSpacing port="source_model" spacing="0"/>

<portSpacing port="source_test set" spacing="0"/>

<portSpacing port="source_through 1" spacing="0"/>

<portSpacing port="sink_averagable 1" spacing="0"/>

<portSpacing port="sink_averagable 2" spacing="0"/>

</process>

</operator>

<connect from_port="input 1" to_op="Validation (9)" to_port="training"/>

<connect from_op="Validation (9)" from_port="model" to_port="result 1"/>

<connect from_op="Validation (9)" from_port="averagable 1" to_port="performance"/>

<portSpacing port="source_input 1" spacing="0"/>

<portSpacing port="source_input 2" spacing="0"/>

<portSpacing port="sink_performance" spacing="0"/>

<portSpacing port="sink_result 1" spacing="0"/>

<portSpacing port="sink_result 2" spacing="0"/>

</process>

</operator>

<operator activated="true" class="set_parameters" compatibility="6.5.002" expanded="true" height="76" name="Set Parameters (6)" width="90" x="246" y="165">

<list key="name_map">

<parameter key="SVM (Linear)" value=" Optimal SVM Linear"/>

</list>

</operator>

<operator activated="true" class="support_vector_machine_linear" compatibility="6.5.002" expanded="true" height="76" name="Optimal SVM Linear (3)" width="90" x="179" y="300"/>

<connect from_port="input 1" to_op="Multiply (9)" to_port="input"/>

<connect from_port="input 2" to_port="output 2"/>

<connect from_op="Multiply (9)" from_port="output 1" to_op="Optimize SVM (3)" to_port="input 1"/>

<connect from_op="Multiply (9)" from_port="output 2" to_op="Optimal SVM Linear (3)" to_port="training set"/>

<connect from_op="Optimize SVM (3)" from_port="parameter" to_op="Set Parameters (6)" to_port="parameter set"/>

<connect from_op="Optimal SVM Linear (3)" from_port="model" to_port="output 1"/>

<portSpacing port="source_input 1" spacing="0"/>

<portSpacing port="source_input 2" spacing="0"/>

<portSpacing port="source_input 3" spacing="0"/>

<portSpacing port="sink_output 1" spacing="0"/>

<portSpacing port="sink_output 2" spacing="0"/>

<portSpacing port="sink_output 3" spacing="0"/>

</process>

<process expanded="true">

<operator activated="true" class="multiply" compatibility="6.5.002" expanded="true" height="94" name="Multiply (10)" width="90" x="45" y="165"/>

<operator activated="true" class="support_vector_machine_libsvm" compatibility="6.5.002" expanded="true" height="76" name="Optimal SVM rbf (3)" width="90" x="246" y="300">

<parameter key="C" value="68146.13250384372"/>

<parameter key="nu" value="0.12334713683253792"/>

<list key="class_weights"/>

</operator>

<operator activated="true" class="optimize_parameters_evolutionary" compatibility="6.5.002" expanded="true" height="112" name="Optimize Parameters (6)" width="90" x="112" y="30">

<list key="parameters">

<parameter key="SVM (rbf).C" value="[0.000000001;100000]"/>

<parameter key="SVM (rbf).nu" value="[0.001;2]"/>

</list>

<parameter key="max_generations" value="10"/>

<parameter key="use_early_stopping" value="true"/>

<parameter key="population_size" value="10"/>

<parameter key="use_local_random_seed" value="true"/>

<parameter key="parallelize_optimization_process" value="true"/>

<process expanded="true">

<operator activated="true" class="parallel:x_validation_parallel" compatibility="5.3.000" expanded="true" height="112" name="Validation (10)" width="90" x="380" y="120">

<parameter key="number_of_validations" value="5"/>

<parameter key="use_local_random_seed" value="true"/>

<parameter key="parallelize_training" value="true"/>

<parameter key="parallelize_testing" value="true"/>

<process expanded="true">

<operator activated="true" class="support_vector_machine_libsvm" compatibility="6.5.002" expanded="true" height="76" name="SVM (6)" width="90" x="179" y="75">

<parameter key="C" value="68146.13250384372"/>

<parameter key="nu" value="0.12334713683253792"/>

<list key="class_weights"/>

</operator>

<connect from_port="training" to_op="SVM (6)" to_port="training set"/>

<connect from_op="SVM (6)" from_port="model" to_port="model"/>

<portSpacing port="source_training" spacing="0"/>

<portSpacing port="sink_model" spacing="0"/>

<portSpacing port="sink_through 1" spacing="0"/>

</process>

<process expanded="true">

<operator activated="true" class="apply_model" compatibility="6.5.002" expanded="true" height="76" name="Apply Model (13)" width="90" x="112" y="120">

<list key="application_parameters"/>

</operator>

<operator activated="true" class="subprocess" compatibility="6.5.002" expanded="true" height="76" name="AUPRC Param Optim II (3)" width="90" x="246" y="120">

<process expanded="true">

<operator activated="true" class="rename" compatibility="6.5.002" expanded="true" height="76" name="Rename (8)" width="90" x="45" y="30">

<parameter key="old_name" value="confidence(N)"/>

<parameter key="new_name" value="noconf"/>

<list key="rename_additional_attributes">

<parameter key="confidence(Y)" value="yesconf"/>

</list>

</operator>

<operator activated="true" class="r_scripting:execute_r" compatibility="6.5.000" expanded="true" height="76" name="Execute R (9)" width="90" x="179" y="30">

<parameter key="script" value="# rm_main is a mandatory function, &#10;# the number of arguments has to be the number of input ports (can be none)&#10;rm_main = function(data)&#10;{&#10; library(PRROC)&#10; data$class[data$icustay_expire_flg== &quot;Y&quot;]&lt;-1 &#10; data$class[data$icustay_expire_flg== &quot;N&quot;]&lt;-0&#10; data$class &lt;- as.numeric(data$class)&#10; x=pr.curve(scores.class0 = data$yesconf, weights.class0 = data$class)&#10; #y=roc.curve(scores.class0 = data$yesconf, weights.class0 = data$class)&#10; &#10; #a&lt;-x$auc.integral&#10; data$AUPRC&lt;- x$auc.integral&#10; #data$AUC &lt;- y$auc.integral&#10; metaData$data$AUPRC &lt;&lt;- list(type=&quot;numeric&quot;, role=&quot;performance&quot;)&#10; #metaData$data$AUC &lt;&lt;- list(type=&quot;numeric&quot;, role=&quot;performance&quot;)&#10; return(data)&#10;}&#10;"/>

</operator>

<operator activated="true" class="extract_performance" compatibility="6.5.002" expanded="true" height="76" name="AUPRC (8)" width="90" x="313" y="30">

<parameter key="performance_type" value="data_value"/>

<parameter key="attribute_name" value="AUPRC"/>

<parameter key="example_index" value="1"/>

</operator>

<connect from_port="in 1" to_op="Rename (8)" to_port="example set input"/>

<connect from_op="Rename (8)" from_port="example set output" to_op="Execute R (9)" to_port="input 1"/>

<connect from_op="Execute R (9)" from_port="output 1" to_op="AUPRC (8)" to_port="example set"/>

<connect from_op="AUPRC (8)" from_port="performance" to_port="out 1"/>

<portSpacing port="source_in 1" spacing="0"/>

<portSpacing port="source_in 2" spacing="0"/>

<portSpacing port="sink_out 1" spacing="0"/>

<portSpacing port="sink_out 2" spacing="0"/>

</process>

</operator>

<operator activated="false" class="performance_binominal_classification" compatibility="6.5.002" expanded="true" height="76" name="Performance (10)" width="90" x="246" y="345">

<parameter key="accuracy" value="false"/>

<parameter key="f_measure" value="true"/>

</operator>

<connect from_port="model" to_op="Apply Model (13)" to_port="model"/>

<connect from_port="test set" to_op="Apply Model (13)" to_port="unlabelled data"/>

<connect from_op="Apply Model (13)" from_port="labelled data" to_op="AUPRC Param Optim II (3)" to_port="in 1"/>

<connect from_op="AUPRC Param Optim II (3)" from_port="out 1" to_port="averagable 1"/>

<portSpacing port="source_model" spacing="0"/>

<portSpacing port="source_test set" spacing="0"/>

<portSpacing port="source_through 1" spacing="0"/>

<portSpacing port="sink_averagable 1" spacing="0"/>

<portSpacing port="sink_averagable 2" spacing="0"/>

</process>

</operator>

<connect from_port="input 1" to_op="Validation (10)" to_port="training"/>

<connect from_op="Validation (10)" from_port="model" to_port="result 1"/>

<connect from_op="Validation (10)" from_port="averagable 1" to_port="performance"/>

<portSpacing port="source_input 1" spacing="0"/>

<portSpacing port="source_input 2" spacing="0"/>

<portSpacing port="sink_performance" spacing="0"/>

<portSpacing port="sink_result 1" spacing="0"/>

<portSpacing port="sink_result 2" spacing="0"/>

</process>

</operator>

<operator activated="true" class="set_parameters" compatibility="6.5.002" expanded="true" height="76" name="Set Parameters (7)" width="90" x="246" y="120">

<list key="name_map">

<parameter key="SVM (rbf)" value="Optimal SVM rbf"/>

</list>

</operator>

<connect from_port="input 1" to_op="Multiply (10)" to_port="input"/>

<connect from_port="input 2" to_port="output 2"/>

<connect from_op="Multiply (10)" from_port="output 1" to_op="Optimize Parameters (6)" to_port="input 1"/>

<connect from_op="Multiply (10)" from_port="output 2" to_op="Optimal SVM rbf (3)" to_port="training set"/>

<connect from_op="Optimal SVM rbf (3)" from_port="model" to_port="output 1"/>

<connect from_op="Optimize Parameters (6)" from_port="parameter" to_op="Set Parameters (7)" to_port="parameter set"/>

<portSpacing port="source_input 1" spacing="0"/>

<portSpacing port="source_input 2" spacing="0"/>

<portSpacing port="source_input 3" spacing="0"/>

<portSpacing port="sink_output 1" spacing="0"/>

<portSpacing port="sink_output 2" spacing="0"/>

<portSpacing port="sink_output 3" spacing="0"/>

</process>

<process expanded="true">

<operator activated="true" class="weka:W-SMO" compatibility="5.3.001" expanded="true" height="76" name="W-SMO (3)" width="90" x="45" y="120"/>

<connect from_port="input 1" to_op="W-SMO (3)" to_port="training set"/>

<connect from_port="input 2" to_port="output 2"/>

<connect from_op="W-SMO (3)" from_port="model" to_port="output 1"/>

<portSpacing port="source_input 1" spacing="0"/>

<portSpacing port="source_input 2" spacing="0"/>

<portSpacing port="source_input 3" spacing="0"/>

<portSpacing port="sink_output 1" spacing="0"/>

<portSpacing port="sink_output 2" spacing="0"/>

<portSpacing port="sink_output 3" spacing="0"/>

</process>

</operator>

<operator activated="true" class="apply_model" compatibility="6.5.002" expanded="true" height="76" name="Apply Model (14)" width="90" x="715" y="165">

<list key="application_parameters"/>

</operator>

<operator activated="true" class="store" compatibility="6.5.002" expanded="true" height="60" name="Store (4)" width="90" x="648" y="300">

<parameter key="repository_entry" value="FilterResults/Models/model%{iterationWeights}%{iteration}"/>

</operator>

<operator activated="true" class="subprocess" compatibility="6.5.002" expanded="true" height="76" name="Subprocess (4)" width="90" x="782" y="345">

<parameter key="parallelize_nested_process" value="true"/>

<process expanded="true">

<operator activated="true" class="rename" compatibility="6.5.002" expanded="true" height="76" name="Rename (9)" width="90" x="45" y="30">

<parameter key="old_name" value="confidence(N)"/>

<parameter key="new_name" value="noconf"/>

<list key="rename_additional_attributes">

<parameter key="confidence(Y)" value="yesconf"/>

</list>

</operator>

<operator activated="true" class="r_scripting:execute_r" compatibility="6.5.000" expanded="true" height="76" name="Execute R (10)" width="90" x="179" y="30">

<parameter key="script" value="# rm_main is a mandatory function, &#10;# the number of arguments has to be the number of input ports (can be none)&#10;rm_main = function(data)&#10;{&#10; library(PRROC)&#10; data$class[data$icustay_expire_flg== &quot;Y&quot;]&lt;-1 &#10; data$class[data$icustay_expire_flg== &quot;N&quot;]&lt;-0&#10; data$class &lt;- as.numeric(data$class)&#10; x=pr.curve(scores.class0 = data$yesconf, weights.class0 = data$class)&#10; #y=roc.curve(scores.class0 = data$yesconf, weights.class0 = data$class)&#10; &#10; #a&lt;-x$auc.integral&#10; data$AUPRC&lt;- x$auc.integral&#10; #data$AUC &lt;- y$auc.integral&#10; metaData$data$AUPRC &lt;&lt;- list(type=&quot;numeric&quot;, role=&quot;performance&quot;)&#10; #metaData$data$AUC &lt;&lt;- list(type=&quot;numeric&quot;, role=&quot;performance&quot;)&#10; return(data)&#10;}&#10;"/>

</operator>

<operator activated="true" class="extract_performance" compatibility="6.5.002" expanded="true" height="76" name="AUPRC (9)" width="90" x="334" y="30">

<parameter key="performance_type" value="data_value"/>

<parameter key="attribute_name" value="AUPRC"/>

<parameter key="example_index" value="1"/>

</operator>

<operator activated="true" class="log" compatibility="6.5.002" expanded="true" height="76" name="Log (3)" width="90" x="514" y="30">

<parameter key="filename" value="C:\Users\Milan\Papers\PLOS I\FilterResults\LogFilterWeights.log"/>

<list key="log">

<parameter key="AUPRC" value="operator.AUPRC (9).value.performance"/>

<parameter key="FeatureSelection" value="operator.Loop (2).value.iteration"/>

<parameter key="Algorithm" value="operator.Loop.value.iteration"/>

<parameter key="k" value="operator.OptimalWeights.parameter.k"/>

</list>

<parameter key="persistent" value="true"/>

</operator>

<connect from_port="in 1" to_op="Rename (9)" to_port="example set input"/>

<connect from_op="Rename (9)" from_port="example set output" to_op="Execute R (10)" to_port="input 1"/>

<connect from_op="Execute R (10)" from_port="output 1" to_op="AUPRC (9)" to_port="example set"/>

<connect from_op="AUPRC (9)" from_port="performance" to_op="Log (3)" to_port="through 1"/>

<connect from_op="Log (3)" from_port="through 1" to_port="out 1"/>

<portSpacing port="source_in 1" spacing="0"/>

<portSpacing port="source_in 2" spacing="0"/>

<portSpacing port="sink_out 1" spacing="0"/>

<portSpacing port="sink_out 2" spacing="0"/>

</process>

</operator>

<connect from_port="input 1" to_op="Select Subprocess (2)" to_port="input 1"/>

<connect from_port="input 2" to_op="OptimalWeights" to_port="example set input"/>

<connect from_op="Select Subprocess (2)" from_port="output 1" to_op="Optimize Parameters (3)" to_port="input 1"/>

<connect from_op="Select Subprocess (2)" from_port="output 2" to_op="Store (3)" to_port="input"/>

<connect from_op="Store (3)" from_port="through" to_op="OptimalWeights" to_port="weights"/>

<connect from_op="Optimize Parameters (3)" from_port="parameter" to_op="Set Parameters (5)" to_port="parameter set"/>

<connect from_op="OptimalWeights" from_port="example set output" to_op="Multiply" to_port="input"/>

<connect from_op="Multiply" from_port="output 1" to_op="Algorithms (3)" to_port="input 1"/>

<connect from_op="Multiply" from_port="output 2" to_op="Apply Model (14)" to_port="unlabelled data"/>

<connect from_op="Algorithms (3)" from_port="output 1" to_op="Apply Model (14)" to_port="model"/>

<connect from_op="Apply Model (14)" from_port="labelled data" to_op="Subprocess (4)" to_port="in 1"/>

<connect from_op="Apply Model (14)" from_port="model" to_op="Store (4)" to_port="input"/>

<connect from_op="Subprocess (4)" from_port="out 1" to_port="output 1"/>

<portSpacing port="source_input 1" spacing="0"/>

<portSpacing port="source_input 2" spacing="0"/>

<portSpacing port="source_input 3" spacing="0"/>

<portSpacing port="sink_output 1" spacing="0"/>

<portSpacing port="sink_output 2" spacing="0"/>

</process>

</operator>

<connect from_port="input 1" to_op="Loop" to_port="input 1"/>

<connect from_port="input 2" to_op="Loop" to_port="input 2"/>

<connect from_op="Loop" from_port="output 1" to_port="output 1"/>

<portSpacing port="source_input 1" spacing="0"/>

<portSpacing port="source_input 2" spacing="0"/>

<portSpacing port="source_input 3" spacing="0"/>

<portSpacing port="sink_output 1" spacing="0"/>

<portSpacing port="sink_output 2" spacing="0"/>

</process>

</operator>

<connect from_op="Read ICUdetail" from_port="output" to_op="Data preparation" to_port="in 1"/>

<connect from_op="Platelet count" from_port="output" to_op="Data preparation" to_port="in 3"/>

<connect from_op="Comorbidity CSV " from_port="output" to_op="Data preparation" to_port="in 2"/>

<connect from_op="Data preparation" from_port="out 1" to_op="Loop (2)" to_port="input 1"/>

<connect from_op="Data preparation" from_port="out 2" to_op="Loop (2)" to_port="input 2"/>

<connect from_op="Loop (2)" from_port="output 1" to_port="result 1"/>

<portSpacing port="source_input 1" spacing="0"/>

<portSpacing port="sink_result 1" spacing="0"/>

<portSpacing port="sink_result 2" spacing="0"/>

</process>

</operator>

</process>
